# Bayesian probabilistic statements for communicating clinical trial results

**DOI:** 10.1186/s12916-026-05208-w

**Published:** 2026-10-08

**Authors:** Orestis Efthimiou, Konstantina Chalkou, George CM Siontis, Urs Fischer, Thomas Perneger, Georgia Salanti, Angèle Gayet-Ageron

**Affiliations:** 1 Institute of Social and Preventive Medicine (ISPM), University of Bern, Bern, Switzerland; 2 Institute of Primary Health Care (BIHAM), University of Bern, Bern, Switzerland; 3 Department of Cardiology, Bern University Hospital, Inselspital, University of Bern, Bern, Switzerland; 4 Department of Neurology, Inselspital, University Hospital Bern and University of Bern, Bern, Switzerland; 5 Faculty of Medicine, University of Geneva, Geneva, Switzerland; 6 Division of Clinical Epidemiology, University Hospitals of Geneva, Geneva, Switzerland

**Keywords:** Bayesian, Probabilistic statements, Posterior probabilities

## Abstract

**Background:**

Clinical trials are often analyzed using frequentist methods and results are interpreted according to their statistical significance. However, p-values and effect measures such as odds or hazard ratios are poorly understood by researchers and policymakers, and even less by patients and clinicians. Moreover, communicating uncertainty around treatment effects is challenging. Probabilistic statements from Bayesian analyses (e.g., the probability that a drug is more effective than placebo) offer an intuitive way to convey results and uncertainty, may provide insight, and support clinical decision-making. We aimed to (i) empirically evaluate the utility of probabilistic statements by re-analyzing data from previously published trials using Bayesian methods, and (ii) provide instructional examples of how probabilistic statements can be used to communicate and interpret results from clinical trials.

**Methods:**

We analyzed data on primary outcomes obtained from trials previously published in leading medical journals, using Bayesian methods. We included 169 primary outcomes from 130 negative trials (p-values > 0.05) across various medical fields, and 234 outcomes from 225 positive trials (p-value < 0.05) in oncology. We used uninformative priors for illustration, supplementing them with skeptical priors in sensitivity analyses. We estimated the probabilities that active treatments were more effective or safe than controls, for each outcome. We also estimated probabilities that the treatment effects were above specific thresholds for clinical significance. We described three of the included trials in more detail and additionally analyzed one trial in neurology as didactical case studies, to illustrate the use of probabilistic statements.

**Results:**

So-called negative studies may sometimes yield clinically relevant evidence of an effect: in 26/169 (15%) cases we found > 90% probability that the experimental treatment is better than control; for 12/169 (7%) of them, this probability was > 95%. Conversely, positive trials may not always show evidence of clinically meaningful effects: in 25/234 (11%) of the positive oncology trials, there was less than 50% probability that treatment reduces hazards by 20% or more. Our case studies illustrate how to use probabilistic statements in medical decision making in practice.

**Conclusions:**

Relying on statistical significance without considering clinical importance can mislead decision-making and potentially harm patients. Probabilistic statements from Bayesian analyses can complement traditional approaches to communicating clinical trial results.

**Supplementary Information:**

The online version contains supplementary material available at https://doi.org/10.1186/s12916-026-05208-w.

## Background

Randomized clinical trials (RCTs) are the cornerstone of evidence-based medicine. They are used for regulatory purposes, the development of clinical recommendations, and for health technology assessment bodies, when making reimbursement decisions. Results of relevant trials may also be integrated in discussions between patients and their doctors, for a shared clinical decision-making. RCTs are typically analyzed using frequentist statistical methods, in which p-values are used to assess the compatibility of the observed data with the null hypothesis, which usually assumes no treatment effect. In practice, p-values are often dichotomized using a conventional threshold for statistical significance, most commonly * p<0.05*.

Recently, however, there has been a plea to move away from this practice [1–3]; it has been argued that for interpreting findings researchers should instead examine the confidence interval (CI) of the estimated treatment effect [4, 5]. However, even then interpretation and communication of findings can be challenging. Consider an RCT where the new treatment reduced the risk of death, with an Odds Ratio (OR) = 0.56, 95% CI [0.29; 1.20], *p* = 0.09. Most clinicians may find it difficult to understand what an OR = 0.56 means, let alone communicating it to a patient [6]. Using the Risk Difference (RD) would improve understanding of the point estimate [7] – although a RD may not always be transportable across populations. However, communicating its uncertainty would still be challenging. Other approaches like likelihood ratios can provide valuable insights [8], though they may be less useful for communicating clinical trial results to a lay audience.

Presenting probabilistic statements about the results of a trial may help mitigate these problems and facilitate communication. Statements like “*There is a 90% probability that the new treatment is effective*”, are simpler, more intuitive and understandable by both clinicians and patients as compared to frequentist statements related to statistical significance. As Lee et al. [9] write “*A typical p-value […] quantifies how unlikely the data are if the treatment does not work […] changing the question of interest from “Does the treatment work?” to “Are the data unlikely if the treatment does not work […] By contrast*,* Bayes […] provides a direct answer to the question “Does the new drug or biologic work?” by computing the probability of treatment benefit”*. Such probabilistic statements can be obtained for any effect measure in a Bayesian framework (and when uninformative priors are used, they are closely related to results obtained via using confidence distributions, a method that bridges the gap between the frequentist and Bayesian statistics [10]). A Bayesian analysis offers additional advantages beyond its ability to yield probabilistic statements. A Bayesian 95% credible interval (CrI) has an intuitive interpretation: “*There is a 95% probability that the true effect lies within the interval*”. In contrast, a frequentist 95% CI should be interpreted as “*95% of such intervals would contain the true effect*,* if the study was repeated an infinite number of times*”; CIs are much harder to grasp, while people often misinterpret CIs as CrIs [11]. A Bayesian framework can further benefit analyses via the inclusion of prior beliefs or external evidence. Generally, a proper Bayesian analysis requires that prior distributions are thoroughly justified and that the impact of different choices should be examined through sensitivity analysis, as also noted in a recent recommendations document from the FDA [12]. 

Despite their well-documented merits and the abundance of pedagogical resources [13–15], most medical researchers remain unfamiliar with Bayesian methods [16]. At the same time, when presented with accurate interpretations, it has been shown that clinicians may find Bayesian results expressed in probabilistic statements and credible intervals to be more useful than p-values and confidence intervals from frequentist analyses [11].

In this didactical article we aim to illustrate the added value of interpreting the results of trials via Bayesian methods in general, and particularly using probabilistic statements. Using trial-level data from 356 RCTs, previously published in high impact-factor medical journals, we show how probabilistic statements may offer insight and help frame results in an easily understood manner. We illustrate how “negative” trials (i.e., p-value > 0.05 using a frequentist approach) may sometimes provide evidence of a treatment effect, while “positive” trials (*p* < 0.05) might offer little evidence of a clinically meaningful effect. Using concrete examples, we highlight the potentially harmful consequences of relying on statistical significance for clinical decision-making. To further clarify these points, we provide a detailed Bayesian re-analysis of a trial examining the optimal timing of anticoagulation therapy in stroke patients. There, we provide additional guidance on the use of informative prior distributions and illustrate how to elicit information from experts in practice. Finally, we provide a web-app, to facilitate the uptake of the methods.

## Methods

 The data and codes needed to replicate all results are provided in https://github.com/esm-ispm-unibe-ch-REPRODUCIBLE/Probabilistic.

### Empirical evaluation: a re-analysis of previously published negative and positive RCTs using Bayesian methods.

#### Data

We reused two datasets with trial-level data of previously published trials. The first dataset (deemed “negative trials” based on conventional statistical significance, i.e., p-value > 0.05) was collected by Perneger and Gayet-Ageron [8]. It includes estimates of treatment effects about primary outcomes from 130 clinical trials published in six high impact-factor general medical journals in 2021. In brief, 48% of them aimed to assess the effect of a new drug; 40% assessed nonpharmacological interventions; and 12% assessed a change in the delivery or dose. We included 169 primary outcomes, i.e., several trials had multiple primary outcomes; all outcomes were statistically non-significant. The second dataset (“positive trials”, based on statistical significance, i.e., p-value < 0.05) was collected by Perneger et al. [17]. It included 225 trials in oncology published between 2009 and 2019. The trials regarded various types of cancers, including blood (38/225, 17%), lung (31/225, 14%), and breast cancer (28/225, 12%). They reported 234 primary outcomes and provided estimates of hazard ratio (HR) for survival, where all estimates were statistically significant. More details are given in the Appendix, Sects. 1&2.

#### Analysis methods

We extracted the published treatment effect and corresponding standard error for each outcome. We present results according to whether the effect measure was a ratio (odd/risk/hazard/incidence rate ratio) or an absolute difference. To make results comparable across trials, we transformed effect measures so that a ratio < 1 or an absolute difference < 0 favored the experimental treatment. For each outcome we fitted a Bayesian model, where we combined a normal likelihood on the extracted data with an uninformative prior distribution. The resulting posterior distribution describes the uncertainty of the true treatment effect; we used it to derive probabilities of various statements, such as the probability that the experimental treatment is better than the control. For ratios, we calculated the probabilities that the effect was below 1, 0.95, 0.90, and 0.80, and we present them in histograms and tables. For negative trials, we additionally: (i) analysed only survival outcomes; (ii) analysed only composite outcomes including survival. For positive trials, aiming to illustrate how a statistically significant finding may not always correspond to a clinically relevant effect, we calculated the probability that the treatment effect exceeded an arbitrarily chosen smallest worthwhile effect (SWE) [18, 19]. Given the potential toxicity of oncologic treatments, we assumed that only a substantial survival benefit would be clinically meaningful, we set the SWE at HR = 0.80, and estimated the probability that the treatment effect reaches this threshold. An HR ≤ 0.8 was recommended in 2014 by the American Society of Clinical Oncology (ASCO) as the minimum effect of new therapies for metastatic solid tumors that can be considered to be clinically meaningful [20]. Although this is a largely arbitrary threshold for our analysis, we used it for illustrative purposes.

As mentioned above, our primary analyses used uninformative priors for illustration purposes, assuming large uncertainty about the effectiveness of the experimental treatment. In practice, however, when relevant information is already available (e.g., often the case in phase III clinical trials) it is recommended to use informative priors. For example, it may be the case there have been many failed trials for closely related drugs, meaning that we are skeptical about the effects of the new drug. Aiming to reflect this scenario, we performed a sensitivity analysis of all trials reporting ratios using a strong skeptical prior distribution, giving 95% prior probability that the true effect (ratio) is between 0.41 and 2.42. More details are provided in the Appendix.

Finally, we selected two negative trials (one for the treatment of acute lymphoblastic leukaemia and one for the treatment of left diaphragmatic hernia) and one positive trial (for the treatment of colorectal cancer), to illustrate the concepts in more detail and to provide examples of how probabilistic statements can be used for communicating and interpreting results from clinical trials. To this end, we generated statements such as: “*Given the trial results*,* there is XX% probability that the new drug reduces the risk of the outcome on average*,” or “*According to this study*,* there is XX% probability that the new drug reduces the average risk of the outcome by YY% or more*.” For the positive trials (all in oncology), we reported probabilistic statements such as: “*There is XX% probability that the treatment effect reaches the smallest clinically meaningful effect.”*

Details about all analyses are provided in Sect.  3 of the Appendix.

### A case study on early versus late anticoagulants for stroke patients with atrial fibrillation: the ELAN trial.

#### Data

What is the optimal timing of treatment with direct oral anticoagulants (DOACs) after an ischemic stroke is an important question; early treatment may reduce the risk of recurrent ischemic stroke but may increase the risk of intracranial hemorrhage. The ELAN trial aimed to investigate the safety and efficacy of administrating DOACs early, defined as treatment within 48 h after minor or moderate stroke and on days 6–7 after a major stroke [21]. The primary outcome was a composite of recurrent ischemic stroke, symptomatic intracranial hemorrhage, systemic embolism, major extracranial bleeding, or vascular death within 30 days. Secondary outcomes were the composite at 90 days, and the components of the composite outcome. ELAN was designed to estimate treatment effects, so no hypotheses were statistically tested. The trial found a reduced risk of the composite outcome with early intervention, with a $\:RD=-1.2\%,\:95\%CI\:[-2.8\%;\:0.5\%]$, and also reduced risk for most secondary outcomes, but with larger uncertainty.

#### Analysis methods

For the primary composite outcome and each of its components at 30 and 90 days, we fit a separate Bayesian model with a binomial likelihood for the probability of an event in each arm, coupled with a uniform $\:U\left(\mathrm{0,1}\right)$ prior. The resulting posterior distribution of the * RD* was used to make probabilistic statements about the effects of early versus late treatment. The uniform prior corresponds to complete ignorance about the risk of an event prior to the trial. In practice, we almost always have some prior belief or external information about the parameters of interest, e.g., from early-stage clinical trials; this information should be encoded into our prior distributions. We thus repeated the analysis for symptomatic intracranial hemorrhage using a skeptical (conservative) informative prior that reflected common concerns among clinicians before the trial, i.e., that early treatment with DOACs may increase the risk of bleeding compared to treating late [22, 23]. This prior assumed that early treatment increased the (absolute) risk of hemorrhage by 1%, with 95% of the probability mass being distributed between 0% and 2%. This corresponds to a prior for RD following a $\:\mathrm{N}(-0.01,{{\upsigma\:}}^{2}=1/30000)$ normal distribution. We also repeated the analysis using an ‘enthusiastic’ prior $\:\mathrm{N}(0.01,{{\upsigma\:}}^{2}=1/30000)$, i.e. one that favors early. As illustration, we also fit an analysis using a family of priors described by one varying parameter. Moreover, in the Appendix we outline methods for eliciting priors from experts.

An advantage of the probabilistic presentation of results is that they can estimate probabilities for complex statements that are of interest to patients; here we estimated the probability that early intervention reduces the absolute risk of recurring stroke by at least 0.5%, and *at the same time* it does not increase the probability of hemorrhage by more than 0.5% ($\:P(R{D}_{stroke}>0.5\%\:\&\:R{D}_{haemorrhage}>-0.5\%)$), assuming independence of the outcomes; these thresholds were chosen to reflect the clinical significance of the outcomes [21].

We additionally performed a simple Bayesian decision analysis to identify the best treatment, by considering the risk of each outcome under each treatment, the corresponding uncertainty, and the relative importance (severity weight) of each outcome. For simplicity, we considered only recurrent ischemic stroke and symptomatic intracranial hemorrhage, assigning equal severity weights to the outcomes. The estimated risks for each outcome under each treatment, combined with the outcome severity weights, provide the “utility” for late and early treatment. Using the utility’s posterior distribution, we identified the probability of each treatment being preferable. In sensitivity analyses we assigned double severity weight to hemorrhage. More details are in the Appendix, Sect. 4. In that section we also outline analyses that relax the assumption of independence between the outcomes, and analyses for posterior predictive checks [24]. 

### A web application to calculate probabilistic statements for the results of a trial

We developed a freely-available web application that uses a trial’s findings to estimate the probability of specific statements being true. For a dichotomous outcome, the user needs to input the number of events and sample size per arm, or the estimated treatment effect with a 95% Cr or CI. Users should note that for rare events or small sample sizes, these two approaches may yield different posterior summaries, as the impact of the prior distributions becomes larger. For continuous or time-to-event data, the user needs to input a (previously estimated) mean difference or hazard ratio respectively, with corresponding uncertainty intervals. For all analyses, the user needs to also provide a reference effect size; this can be the null effect or another value deemed relevant, such as the HR = 0.8 that we used in our evaluation of the positive studies. The app provides the posterior distribution of the true effect size and calculates the probability that this effect is larger than the reference. Users should also note that this app only implements uninformative priors; as discussed in this paper, however, the use of informative priors is highly recommended in practice, unless there is truly no knowledge about the key parameters prior to the trial (e.g., for early-stage clinical trials).

The app is available in https://oefthimiou.shinyapps.io/BayesianProbabilities/. Additional details are in the Appendix, Sect.  5.

## Results from negative and positive trials

### Negative trials

**Fig. 1:**
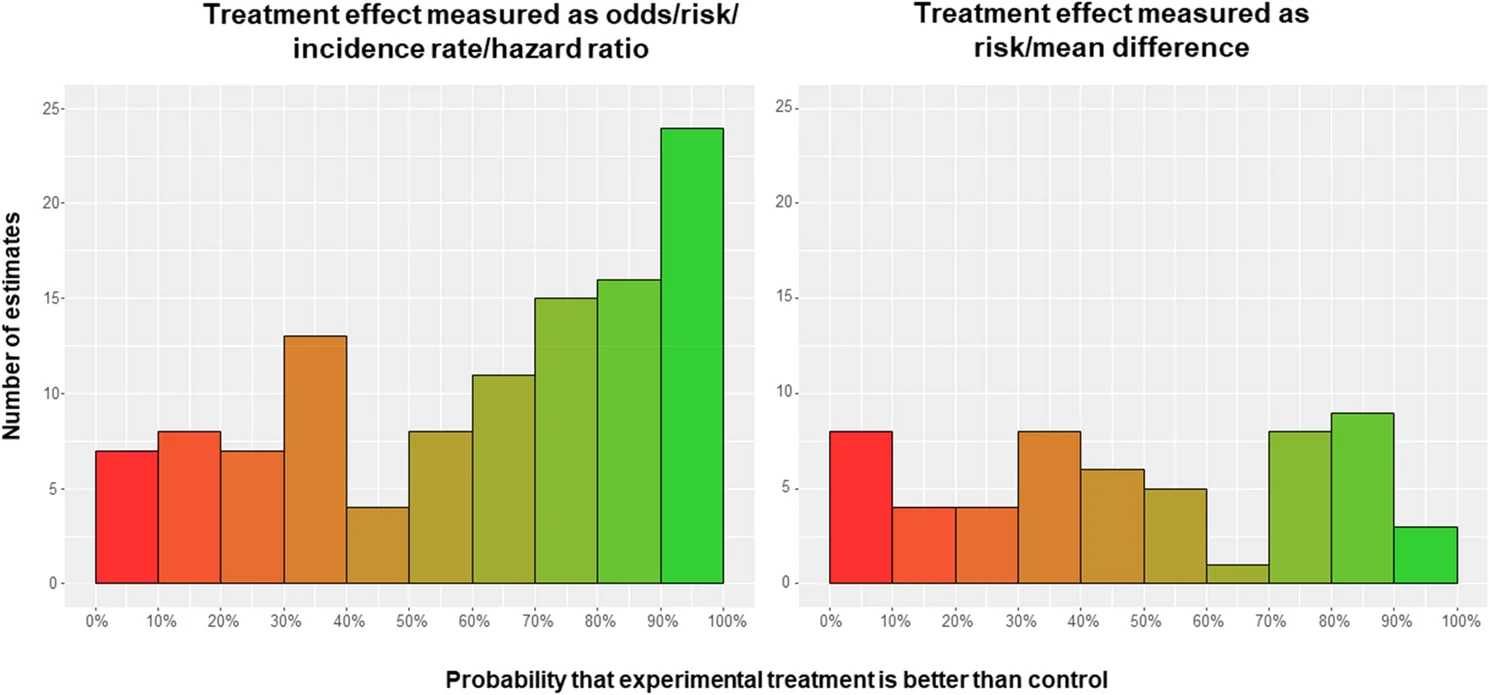
Probabilities that the experimental treatment is better than the control, obtained from primary outcomes of “negative” RCTs. The left (right) panel shows estimates on relative (absolute) scales. Bars colored in light green correspond to outcomes where the probability was > 90% (also shown in x-axis)

 Among the 169 primary outcomes included in the 130 negative trials, 113 (66.9%) reported the treatment effect using ratios (of which 18 with survival outcomes, 28 with composite outcomes), while 56 (33.1%) reported it as differences. The left panel of Fig. 1 shows results for ratios (*n* = 113) and the right panel for risk/mean differences (*n* = 56). Although all ratios were statistically “non-significant”, for 23/113 (20%) of the outcomes there was > 90% probability that the treatment is superior to control. For 10/113 (9%), this probability was > 95%. Even when using a heavily skeptical prior, for 20/113 of the outcomes the probability was > 90%, and for 8/113 it was > 95%.

In 3/56 (5%) of the differences, the probability that the experimental treatment is better than control was ≥ 90%. When limiting analyses to survival outcomes, we found that in 5/18 (28%) instances the estimated probability that the experimental treatment offered a benefit was ≥ 90%; in 3/18 (17%) cases the probability that treatment increased survival was ≥ 96%. For composite outcomes including survival, these numbers were 5/28 (18%) and 2/28 (7%) respectively. Detailed results are available in the Appendix, Sect. 6. In Boxes 1 and 2 we provide examples of two negative trials, and discuss how discarding evidence as “non-significant” may have harmful implications [25, 26].

Box 1: a negative trial in acute lymphoblastic leukemia A trial examined whether substituting 2-cycles Blinatumomab with multiagent chemotherapy in consolidation therapy would improve survival in children, adolescents, and young adults with high- and intermediate-risk first relapse of B-cell acute lymphoblastic leukemia [25]. The primary outcome was disease-free survival, with estimated HR=0.70 [95% CI 0.47; 1.03]. The authors concluded that “*Post reinduction treatment with blinatumomab compared with chemotherapy*,* followed by transplant*,* did not result in a statistically significant difference in disease-free survival*”. However, our re-analysis showed 96% probability that the treatment is better than chemotherapy, and 74% probability that treatment reduces the hazard of relapse by 20% or more (HR<0.80); a frequentist analysis based on confidence distributions gave similar results. Discarding such evidence as statistically non-significant may lead to inappropriate treatment decisions and deprive patients of potentially beneficial therapies.

Box 2: a negative trial in fetal surgery for moderate left diaphragmatic hernia A trial compared fetoscopic endoluminal tracheal occlusion (FETO) vs. control prenatal surgeries for moderate left diaphragmatic hernia [26]. Primary outcomes were (1) survival to discharge from a neonatal intensive care unit, and (2) survival without oxygen supplementation at 6 months. Results for both outcomes suggested lower risks in FETO but were statistically non-significant. The published relative risk was 0.79 [0.61;1.01] for the first outcome, 0.81 [0.61;1.08] for the second, where RR<1 favors FETO. The study also analyzed two secondary outcomes: preterm, prelabor rupture of membranes and preterm birth; both were statistically significant in favor of the control. The authors concluded that *“This trial […] did not show a significant benefit of FETO […] with respect to survival to discharge or the need for oxygen supplementation at 6 months. FETO increased the risks of preterm*,* prelabor rupture of membranes and preterm birth.”* After re-analyzing the data we estimated 97% probability that FETO increases survival at discharge compared to control, and 96% probability that it increases survival without oxygen at 6 months. We also found 86% and 84% probability that FETO increases absolute chances of survival by >5% at discharge and respectively at 6 months. Dismissing the evidence on increased survival with FETO as “statistically non-significant” and continuing to administer the control intervention because it leads to reduced risk of secondary outcomes is highly questionable. Dichotomizing the evidence according to an arbitrary threshold on p-values to guide clinical practice may lead to avoidable deaths.

**Figure:**
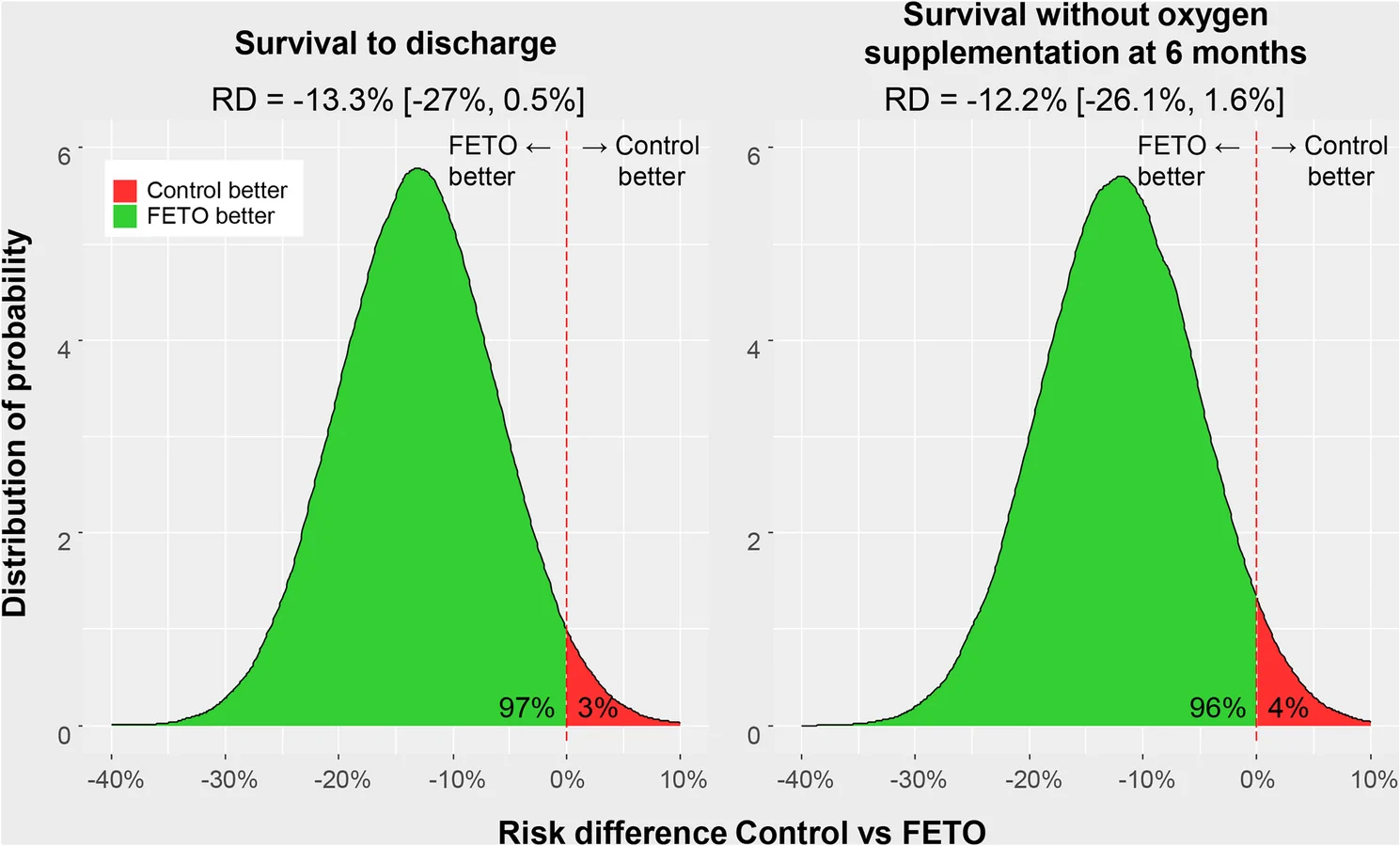

### Positive trials

**Fig. 2:**
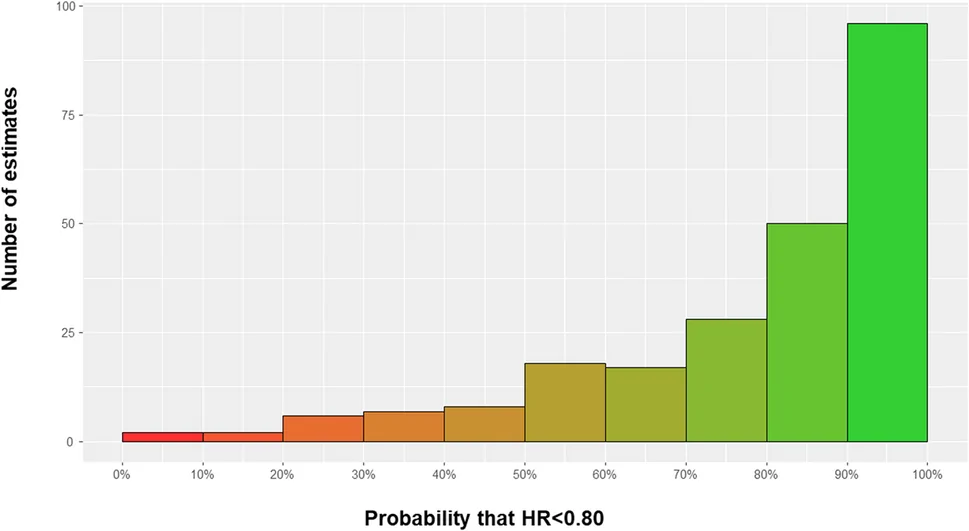
Results from 234 statistically significant estimates on survival from 225 trials in cancer, showing the posterior probability that the treatment effect (Hazard ratio, HR) is clinically meaningful, defined as ≤ 0.80 followingthe American Society of Clinical Oncology [20], where smaller values favor the experimental treatment over the control. The graph shows that although all estimates were significant, there were cases with no evidence that the effect is clinically meaningful. In 25/234 of the outcomes, it was more likely that the treatment effect did not reach the threshold of HR = 0.80. In these outcomes, the probability that the treatment is clinically meaningful was less than 50% (bars with red colors in the graph)

The Bayesian re-analysis of 234 outcomes yielded strong evidence that experimental treatments were superior to control, with corresponding posterior probability > 97% for all outcomes. Next, we estimated the posterior probability that the experimental treatment reached the (arbitrarily chosen) threshold of clinical significance (SWE), HR = 0.80. Figure 2 shows the distribution of the probabilities that the effect of the experimental treatment reaches the SWE, across all outcomes. We found that for 25/234 (11%) outcomes there was < 50% probability that the effect surpassed the SWE threshold. In these cases, it was more likely that the experimental treatment effect was not clinically meaningful, despite the benefit in survival. In the sensitivity analysis with a strong skeptical prior, this was even more pronounced, with 31/234 (13%) outcomes having a probability < 50%. In Box 3 we examine a positive trial in more detail [27]. Full results are shown in the Appendix, Sect. 7.

Box 3: a positive trial in metastatic colorectal cancer A trial compared a chemotherapy regimen, FOLFIRI, with and without the addition of cetuximab as first-line treatment for metastatic colorectal cancer [27]. The combination therapy resulted in a significant improvement in overall survival, with HR for death 0.88 (95% CI, [0.77; 1.00], p-value=0.04). A Bayesian analysis showed that this corresponds to 98% probability that the combination therapy is better than the control. The median survival time was 19.9 [18.5; 21.3] months for the combination therapy compared to 18.6 [16.7; 19.8] for FOLFIRI alone.

Combination therapy, however, was associated with higher risk of adverse events, including neutropenia and leukopenia, which increase susceptibility to infection. Common side effects included diarrhea, vomiting, rash, and dermatitis, which can negatively affect patients’ quality of life. The overall risk of experiencing any side effect was 15-20% higher with combination therapy compared to the control. Given this toxicity, a substantially greater benefit might be required for the combination therapy to be worthwhile, as the negative impact on quality of life may outweigh the modest survival advantage. If we (arbitrarily) set the threshold for smallest worthwhile effect (SWE) at HR=0.80, we find only 8% probability that the combination therapy is worthwhile.

**Figure:**
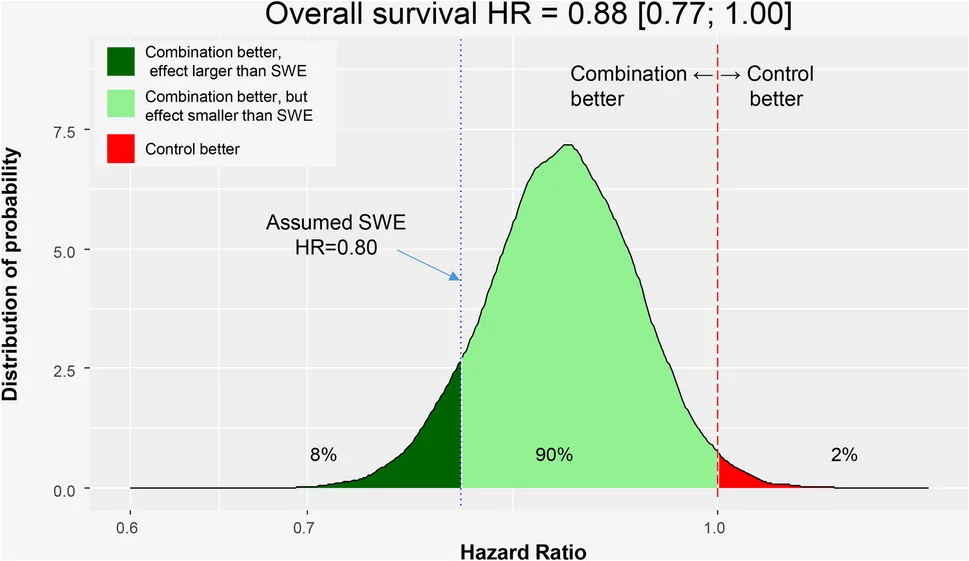

## Results from the study on early versus late anticoagulants

 In **eTable 3** we show the estimated probabilities that early intervention is safer than late for all outcomes at 30 and 90 days, following a Bayesian analysis of the data from the ELAN trial using uninformative priors. Results suggest that early administration of DOACs may reduce the risk of the composite outcome (93% probability that early is superior at 30 days, 97% at 90 days), recurrent ischemic stroke (96% probability that early is superior at both 30 and 90 days), and systemic embolism (91% and 94% at 30 and 90 days). For the remaining outcomes the evidence was weaker. There was no evidence that early treatment increases the risk of symptomatic intracranial hemorrhage, which was hypothesized before the trial.

We also estimated a 97.6% probability that early intervention does not increase the risk of symptomatic intracranial hemorrhage by more than 0.5% as compared to late. If we set the threshold at 1% risk difference, we find a 99.995% probability, i.e. a risk of only 1 in 20,000, that early intervention increases the risk of symptomatic intracranial hemorrhage by 1% or more as compared to late. When we used a strong informative prior in favor of late intervention for the hemorrhage outcome, we found only 7% posterior probability that an early intervention increases the risk by 0.5% or more. Thus, even if we start with a strong belief that an early intervention increases the risk of symptomatic intracranial hemorrhage, the updated evidence suggests that, even if there is an increased risk, it is highly unlikely that it is clinically relevant. When we used an enthusiastic prior, this probability was 0.2%. Further, we estimated $\:P\left(R{D}_{stroke}>0.5\%\:\&\:R{D}_{hemorrhage}>-0.5\%\right)=81\%$, which means there are 4 chances in 5 that early administration of DOACs reduces the risk of recurrent ischemic stroke by at least 0.5% without raising the risk of symptomatic intracranial hemorrhage by 0.5% or more.

In the Bayesian decision analysis we found that, if we equally want to avoid recurrent ischemic stroke and symptomatic intracranial hemorrhage, there is 95% probability that early treatment is preferable. Note that by assigning equal severity weights, given that recurrent ischemic stroke occurs more often than symptomatic intracranial hemorrhage, the utility function is more heavily impacted by the former, rather than the latter. In a sensitivity analysis we assumed a double (triple) severity weight for hemorrhage and found a 91% (87%) probability that early intervention is preferrable, from a decision-analytic perspective.

## Discussion

Statements regarding the probability of whether a new treatment is better than control may be easily understood by researchers, policy makers, clinicians, and even patients, potentially facilitating a shared clinical decision-making process. Such probabilistic statements can be readily obtained via Bayesian analyses. In this didactical paper we aimed to illustrate how to use such statements in the communication and interpretation of clinical trial results. To this end, we performed a small scale empirical study, where we re-analyzed a sample of previously published RCTs [8, 17]. We found that trials deemed conventionally “negative” (p-value > 0.05) may sometimes provide evidence of a clinically meaningful effect, despite the lack of statistical significance. Conversely, “positive” trials (p-value < 0.05) may sometimes fail to show a clinically meaningful effect, despite yielding statistically significant results. In their paper, Perneger et al. [17] showed that the uncertainty of results was only discussed in 12.4% of positive trials; in the current paper, we used specific examples to illustrate how probabilistic statements can be used to convey uncertainty, provide insight, and potentially change the interpretation of trial findings, and how focusing on statistical significance may lead to potentially inappropriate treatment decisions.

One limitation of our work is that we did not access individual patient data from the trials, and our analyses required some assumptions (e.g., normality of estimated effects), while we did not use the exact binomial likelihood for binary outcomes; however, given that our aims were purely didactical, trial-level data was sufficient. A second limitation of what we presented (which was based on study-level data), is that in such cases, statements about average treatment effects may be confused with statements about patient-level effects (e.g., to erroneously interpret the statement “*there is a 97% probability that the drug is more effective than placebo on average*” as if it means “*there is a 97% probability that the drug will benefit this specific patient*”, or “*there is 97% probability that the patient will have a positive outcome*,* if they receive the drug*”); this is especially problematic when there is large treatment effect heterogeneity, and care needs to be taken to communicate correctly. Including and stressing the “on average” part in the statement above may help avoid this confusion and clarify that the estimated effect refers to a population-level treatment benefit rather than the individual patient level. A related limitation is that in this work we did not prospectively assess the added benefit of using Bayesian statistics for communicating effects as was done e.g., by Bray et al. [11]; this was outside our scope, which was mainly didactical, aiming to motivate the use of the methods. A future trial may randomize participants (ideally both clinicians and patients) to different communication methods, in order to assess the relative benefits of the methods and whether indeed Bayesian statistics may benefit the shared decision-making process; a similar trial was previously conducted by Ostinelli et al. in a different but related context [28]. However, it should be noted that although we did not explicitly test the effectiveness of Bayesian methods in communication, it has been argued that presenting information in several formats may help avoiding framing biases in the perception of the message [29], and that visual aids may improve patients’ understanding of probabilistic information [30]; the methods we described in this paper go into this direction. Another limitation is that for analyses of the positive trials in oncology, for illustration purposes we assumed a hazard ratio of 0.80 as the SWE for all outcomes. Although this was grounded in a previous recommendation from the American Society of Clinical Oncology [20], we acknowledge that deciding on an SWE is context-specific, e.g., it should be selected based on the toxicities of the experimental and the control treatment, or it may be patient-specific [18]. Involving patient or public representatives in selecting the smallest worthwhile effect (SWE) threshold would have been challenging for this paper, given the diversity of cancers and treatments included in our reanalysis. Further, with the exception of the ELAN example, our primary analyses utilized “uninformative” priors. While incorporating prior domain knowledge is arguably a major advantage of Bayesian modeling, our focus was didactical and particularly focused on the use of probabilistic statements. Thus, we performed analyses using uninformative priors for illustration, supplementing these with sensitivity analyses using skeptical priors. This, however, should not be interpreted as a recommendation for practice; in practice the use of uninformative priors may rarely be optimal. Researchers almost never start from a state of total ignorance about the parameters of interest prior to their study. Uninformative priors disregard this information, potentially leading to worse estimation, particularly in cases where the new data is limited. Constructing informative distributions requires rigorous methodology; ideally, prior distributions should be elicited by multiple experts in the field, to create a “community of priors”; [14] methods and tools to elicit informative prior distributions have been described elsewhere [14, 31–33], and we briefly outline one approach in the Appendix. Of note, using uninformative priors is closely related to using “confidence distributions” [10], a frequentist method for constructing distributions that are similar to Bayesian posterior distributions. Another application of Bayesian methods in clinical trials, which we did not touch upon, regards the designing of new trials [13]. Bayesian statistics can also be used in meta-analysing results from multiple trials [34], and for almost any type of medical research, e.g., for diagnostic tests, estimating associations, prevalences, etc.

As we mentioned in the introduction, there have been calls to abandon “statistical significance” testing [1, 2]; with this work we do not advocate the replacement of a threshold on p-values with a threshold on posterior probabilities. Before deciding on whether to treat patients with a new treatment, ideally a decision (or cost-effectiveness) analysis should be implemented, considering multiple outcomes, patient preferences, costs, etc. We hereby provided an outline of a simple Bayesian decision analysis for the ELAN trial.

## Conclusions

We illustrated how Bayesian statistics, and particularly probabilistic statements, can be used to refine the presentation and potentially enhance the communication of scientific evidence, thus empowering a shared, evidence-based medical decision-making process. The involvement of patients and representatives of the public is needed to improve the communication of clinical trials’ findings in specific medical contexts, and probabilistic statements may facilitate this process. Such statements can be provided in addition to estimates of treatment effects and credible intervals, or even in parallel with results from frequentist analyses, such as estimated treatment effects and confidence intervals.

## Supplementary Information

Below is the link to the electronic supplementary material.

Supplementary Material 1

## Data Availability

All data used in this paper is available online in [https://github.com/esm-ispm-unibe-ch-REPRODUCIBLE/Probabilistic](https:/github.com/esm-ispm-unibe-ch-REPRODUCIBLE/Probabilistic) .

## Abbreviations

RCT: Randomized clinical trial
OR: Odds ratio
HR: Hazard ratio
RD: Risk difference
SWE: Smallest worthwhile effect
ELAN: Early versus Late initiation of direct oral Anticoagulants in post-ischaemic stroke patients with atrial fibrillation: an international, multicentre, randomised-controlled, two-arm, assessor-blinded trial
FETO: Fetoscopic endoluminal tracheal occlusion
