# Supplementary material for: Bayesian probabilistic statements for communicating clinical trial results
Source: BMC Med. 2026 Oct 8;24:554. doi: 10.1186/s12916-026-05208-w (PMC13651341; doi:10.1186/s12916-026-05208-w)
Supplement: Supplementary file 1 [file 12916_2026_5208_MOESM1_ESM.docx]

**Appendix to the paper:**

**Bayesian probabilistic statements for communicating clinical trial results**

Orestis Efthimiou^1,2^, Konstantina Chalkou^1^, George CM Siontis^1,3^, Urs Fischer^4^, Thomas Perneger^5,6^, Georgia Salanti^1*^, Angèle Gayet-Ageron^1*^***co-last authors**

*^1^Institute of Social and Preventive Medicine (ISPM), University of Bern, Bern, Switzerland*

*^2^Institute of Primary Health Care (BIHAM), University of Bern, Bern, Switzerland*

*^3^Department of Cardiology, Bern University Hospital, Inselspital, University of Bern, Bern, Switzerland*

*^4^Department of Neurology, Inselspital, University Hospital Bern and University of Bern, Bern, Switzerland*

*^5^ Faculty of Medicine, University of Geneva, Geneva, Switzerland*

*^6^Division of Clinical Epidemiology, University Hospitals of Geneva, Geneva, Switzerland*

# Section 1. The negative trials dataset

The “*negative trials*” dataset was previously collected by Perneger and Gayet-Ageron.^1^ The authors conducted a cross-sectional study to identify statistically nonsignificant results in reports of randomized clinical trials published in the Annals of Internal Medicine, BMJ, JAMA, Lancet, New England Journal of Medicine, and PLoS Medicine, in 2021. The dataset includes treatment effects about primary outcomes estimated from a total 130 trials. Most trials (80.8%) included 2 randomized groups, the rest were either multi-arm trials (11.5%) or had a factorial design (7.7%). Most of the experimental interventions that were tested in the trials were substances (i.e., drugs, supplements, biologics, vaccines; 47.7%) and corresponding delivery methods or dose variations (12.3%). The rest (40%) were non-pharmacological interventions (such as devices, surgery techniques, diagnostic methods, behavioral or exercise interventions, etc). Control treatments were usual care (37.7%), active interventions (34.6%), placebo or sham interventions (27.7%). In total, 169 primary outcomes were included (larger than the number of trials, as several trials had two co-primary outcomes). As effect measures the trials used mostly ratios (e.g., odds/risk/hazards/incidence rate ratios; 66.2%). The remaining 33.8% used either mean differences or risk differences. More details are provided in the original publication,^1^ where they provided the full dataset (also available in the online supplement of the present paper <https://github.com/esm-ispm-unibe-ch/Bayesian_reanalysis>).

# Section 2. The positive trials dataset

The “*positive trials*” dataset was previously collected by Perneger et al.^2^ The authors included RCTs in oncology reporting a statistically significant finding with respect to overall survival for a new treatment (pharmacotherapy or radiotherapy). The authors only collected treatment effects expressed as hazard ratios (HR). Trials were published in the New England Journal of Medicine, Lancet, Lancet Oncology, Journal of Clinical Oncology, JAMA, and JAMA Oncology, between January 1^st^, 2009 and June 30^th^, 2019. The dataset includes the HR from each trial (or two HRs, when there were two prespecified co-primary outcomes) and corresponding confidence intervals. A total of 225 trials were included, reporting on 234 HRs that showed a statistically significant survival benefit of the experimental cancer treatment. Most trials (89.3%) were phase III. In 50% of the trials, the primary outcome was overall survival. The trials were on patients with cancers of the blood (17%), lung (14%), breast (12.3%), prostate (10%), colon/rectum (8%), stomach and isophagus (8%), skin (7%), pancreas (4%), liver (4%), uterus or ovary (4%), head and neck (3.1%), urinary tract and kidney (3%), brain (3%), or sarcoma (2%). This is summarized in **eTable 1** below.

The median sample size in the trials was 508 patients. Below we provide a histogram of the sample size.

eFigure 1: Histogram of sample sizes in the positive trials dataset

More details about the trials are provided in the original publication by Perneger et al.,^2^ where they give free access to the full dataset (also available in the online supplement of the present paper <https://github.com/esm-ispm-unibe-ch/Bayesian_reanalysis>).

eTable 1: Breakdown of the positive studies in oncology, by site of cancer.

| **Site** | **N** | **percent** |
| --- | --- | --- |
| **Blood** | 38 | 17% |
| **Lung** | 31 | 14% |
| **Breast** | 28 | 12% |
| **Prostate** | 22 | 10% |
| **Colon/rectum** | 19 | 8% |
| **Stomach & esophagus** | 17 | 8% |
| **Skin** | 15 | 7% |
| **Liver** | 10 | 4% |
| **Pancreas** | 10 | 4% |
| **Head/neck** | 7 | 3% |
| **Brain** | 6 | 3% |
| **Ovary** | 6 | 3% |
| **Kidney** | 5 | 2% |
| **Sarcoma** | 5 | 2% |
| **Uterus** | 4 | 2% |
| **Urinary** | 2 | 1% |

# Section 3. Analyses of the positive and negative trials

For each outcome we fitted a Bayesian model with a normal likelihood; for this, we assumed asymptotic normality of the previously estimated effects. This assumption is more plausible for larger sample sizes; however, given the didactic aim of this paper, we did not further explore the implications of possible violations. Given the estimated $i^{th}$ treatment effect ($\hat{TE}_{i}$), and corresponding standard error (${se\hat{TE}}_{i})$, we fit the following Bayesian model:

Likelihood:

$\hat{TE}_{i}\sim N\left( \mu_{i}, \sigma^{2}={se\hat{TE}}_{i}^{2} \right)$

Prior distribution:

$$\mu_{i}\sim N(0, \sigma^{2}=1000)$$

where $\mu_{i}$ is the target estimand, i.e., the true treatment effect $i$. The second part of the model above corresponds to the prior distribution, and it is “uninformative”, i.e., it has a very large variance.

In sensitivity analysis we used a less vague prior $\mu_{i}\sim N(0, \sigma^{2}=10)$, and a skeptical prior $\mu_{i}\sim N(0, \sigma^{2}=1/5)$. We performed these sensitivity analyses for negative trials that reported results using ratios, and for all positive trials (which reported effects using hazard ratios). The skeptical prior corresponds to a distribution of the effect (the ratio, i.e., $\exp\left( \mu\right))$centered around 1, with 95% of the mass being from 0.41 to 2.42; this reflects the usual situation where the effect of the experimental drug is not expected to be huge. The graph below illustrates this prior:

We fitted the model via Markov Chain Monte Carlo in R using the rjags library.^3,4^ We used 4 chains, 10,000 iterations each, after an initial adaptation of 500 iterations. We checked convergence by visually checking the posterior distribution and the mixing of the chains, and via R-hat. The data and R codes required to replicate all analyses are freely available in the following link:

<https://github.com/esm-ispm-unibe-ch/Bayesian_reanalysis/tree/main/>.

# Section 4. The ELAN trial

## 4.1 Overview

The data and R codes required to replicate all analyses described below are freely available in the following link

<https://github.com/esm-ispm-unibe-ch/Bayesian_reanalysis/tree/main/analysis%20of%20ELAN>.

We fit all models described below via Markov Chain Monte Carlo in R using the rjags library.^3,4^ We used 4 chains, 10,000 iterations each, after an initial adaptation of 500 iterations. We checked convergence by visually checking the posterior distribution and the mixing of the chains, and via R-hat.

## 4.2 Data

Below we show the data we used for the main analysis.

eTable 2: Data from the ELAN trial.

|  |  | **Early intervention** | | **Late intervention** | |
| --- | --- | --- | --- | --- | --- |
|  | **Outcome** | **Events** | **Total** | **Events** | **Total** |
| **30 days** | **Composite outcome (primary)** | 29 | 1006 | 41 | 1007 |
|  | **Recurrent ischemic stroke** | 14 | 984 | 25 | 991 |
|  | **Symptomatic intracranial haemorrhage** | 2 | 984 | 2 | 991 |
|  | **Systemic embolism** | 4 | 984 | 9 | 991 |
|  | **Major extracranial bleeding** | 3 | 984 | 5 | 991 |
|  | **Vascular death** | 11 | 984 | 10 | 991 |
| **90 days** | **Composite outcome** | 36 | 1006 | 54 | 1007 |
|  | **Recurrent ischemic stroke** | 18 | 968 | 30 | 965 |
|  | **Symptomatic intracranial haemorrhage** | 2 | 968 | 2 | 965 |
|  | **Systemic embolism** | 4 | 968 | 10 | 965 |
|  | **Major extracranial bleeding** | 3 | 968 | 8 | 965 |
|  | **Vascular death** | 17 | 968 | 16 | 965 |

## 4.3 Description of the main analysis

We used the data of **eTable 2** to fit a Bayesian model with a binomial likelihood for each treatment arm and obtain the posterior distribution of the risk difference for each different outcome. In more detail, for each outcome we used the number of events and total number of patients for each arm, i.e. $f_{early}, N_{early}, f_{late}, N_{late}$ to fit the following model:

Likelihood:

$$f_{early}\sim Binomial\left( p_{early}, N_{early} \right)$$

$$f_{late}\sim Binomial\left( p_{late}, N_{late} \right)$$

Prior distributions:

$$p_{early},p_{late}\sim Uniform(0,1)$$

Definition of the measure of interest:

$$RD=p_{late}-p_{early}$$

## 4.4 Using alternative priors

For the analysis with the skeptical prior for the outcome of symptomatic intracranial haemorrhage, we used the following model instead:

Likelihood:

$$f_{early}\sim Binomial\left( p_{early}, N_{early} \right)$$

$$f_{late}\sim Binomial\left( p_{late}, N_{late} \right)$$

Prior distributions:

$$p_{early}\sim Uniform(0,1)$$

$$RD\sim N(-0.01, \sigma^{2}=\frac{1}{30000})$$

$$p_{late}=RD+p_{early}$$

This particular prior for the risk difference spans mostly negative values: it is centered around -1% and has the 95% of the mass of the probability distribution between -2.1% and +0.1%. This reflects a strong prior belief that early treatment increases the risk as compared to late, i.e., $RD=p_{late}-p_{early}<0$.

For the analysis with the enthusiastic prior, we used instead $RD\sim N(+0.01, \sigma^{2}=\frac{1}{30000})$, i.e., centered around +1%, with 95% of the mass of the probability distribution between -0.1% and +2.1%.

Finally, as a further analysis to illustrate how we can assess the impact of the choice of prior on the estimated risk difference and corresponding posterior probabilities, we created a family of priors, $RD\sim N(prior\_mean, \sigma^{2}=\frac{1}{30000})$, where $prior\_mean$ was allowed to vary between -1% to 1%. We ran an analysis for 20 values within this range, obtained the posterior probability of $p(RD<-0.05\%)$ for each value of $prior\_mean$, and plotted them in a scatterplot.

## 4.5 Eliciting prior information from experts

Ideally, in a Bayesian analysis we should elicit prior information from experts in the field, and there are several methods to perform such an elicitation.^5^ Here, we outline a mock elicitation procedure for the outcome of symptomatic intracranial haemorrhage, based on the Sheffield Elicitation Framework (SHELF) and corresponding tools (<https://shelf.sites.sheffield.ac.uk/home>), and particularly <https://jeremy-oakley.shinyapps.io/SHELF-single/>. Although we did not use it for what follows, there is an accompanying R package^6^ and another tool, MATCH, also based on SHELF.^7^

In what follows we describe the elicitation based on the roulette method, which users tend to find easier.^7^ . In this method, the expert is provided with a grid of equally sized bins that cover the range of possible risks (0 to 1), and is asked to allocate chips between the bins. The probability of X lying in a particular bin is interpreted as the proportion of chips allocated to that bin. Let us assume that the expert provided the following input for the risk of symptomatic intracranial haemorrhage in the early intervention:

Based on this input, a Beta distribution is recreated by the tool: Beta(1.1, 27.8).

This can in turn be used as a prior in our model above, i.e. $p_{early}\sim Beta(1.1, 27.8)$; likewise for late intervention.

Ideally, information from multiple experts should be combined, e.g. using the tool here <https://jeremy-oakley.shinyapps.io/SHELF-multiple/>. Finally, the information elicited from each can be weighted, e.g. according to their years of experience;^8^ for the case of eliciting a probability (as in the running example) this leads to a prior $Beta(\sum_{k} w_{k}a_{k}, \sum_{k} w_{k}b_{k})$, where $w_{k}$ is the weight assigned to expert $k$, and $a_{k}$, $b_{k}$ are the parameters of the corresponding elicited Beta distribution.

## 4.6 Posterior predictive checks

We performed a posterior predictive check. This type of analysis allows us to see how well the model fits the data; it simulates new data sets using the fitted model parameters and then it compares features of the replicated data set with the ones of the original data set. If the real data looks like an outlier compared to what the replicated data show, it indicates possible model misspecification. For this illustration, we used the outcome of symptomatic intracranial haemorrhage. We did posterior predictive checks for three analyses, i.e., with uninformative / skeptical / enthusiastic prior. We created a range of replicated data using the predictive distributions from the models, using the posterior distributions of the probability of an event in each group and simulating the number of events via binomial draws. From these, we calculated the risk difference. We finally compared the observed (real) risk difference with the distribution of risk differences shown in the replicated data. We also calculated the percent of replications where the risk difference was larger than the observed. This percent, sometimes called Bayesian (posterior predictive) p-value^9^, should be around 50% for a good fit. Values near 0 or 1 indicate a poor fit.

## 4.7 Description of the decision analysis

For illustration purposes, we performed a simple Bayesian decision analysis using the ELAN trial data. This type of analysis tries to identify the best treatment decision by considering the effects of competing treatment options on different outcomes, the corresponding uncertainty, and the relative importance (severity weight) of the outcomes, e.g. as elicited through information on patient preferences.

In more detail, for a decision analysis we need to: (1) use all important efficacy and safety outcomes; (2) estimate the risk of all outcomes under all treatments, and the corresponding uncertainty of the estimates; (3) define severity weights, quantifying how much each outcome is valued by the patients; and (4) identify the treatment that maximizes the total “utility” by integrating the posterior distribution for each outcome under each treatment, multiplied by the corresponding severity weight. The utility function quantifies in a mathematical way the preferences of a decision maker and allows a numerical comparison of the effects of the various treatment options across multiple outcomes.

Here, for simplicity we performed a decision analysis based on two outcomes only, recurrent ischemic stroke and symptomatic intracranial haemorrhage at 30 days. Since both outcomes are negative, we calculate a total “disutility” (i.e., harm), associated with each treatment; the aim is to find the treatment that minimizes harm. We assumed that we are equally interested in avoiding both outcomes and we set a severity weight=1 to each outcome. The total harm folllowing each intervention is calculated using the probability $p$ of each outcome and the corresponding severity weight of each outcome, i.e.,

$$H_{early}=1\times p_{early}^{stroke}+1\times p_{early}^{haemorrhage}$$

and likewise for late:

$$H_{late}=1\times p_{late}^{stroke}+1\times p_{late}^{haemorrhage}$$

The difference between the two harms is $\Delta H=H_{late}-H_{early}$. When $\Delta H>0$, it means that the harm is larger with late treatment, i.e., early is superior. When $\Delta H<0$, late is superior. By substituting we see that $\Delta H$ only depends on the risk difference ($RD$), i.e.,

$$\Delta H=H_{late}-H_{early}={RD}^{stroke}+{RD}^{haemorrhage}$$

Thus, to calculate the posterior distribution of $\Delta H$, we only need estimates of the two risk differences. To this aim, we fit a joined model for the two outcomes, following similar procedures as the ones described in the previous section. We then use the posterior of $\Delta H$ to make probabilistic statements about each treatment being preferable. The R code required to run this analysis is available in <https://github.com/esm-ispm-unibe-ch/Bayesian_reanalysis/tree/main/analysis%20of%20ELAN>.

Of note, the two outcomes may be correlated. Such correlation could be induced via patient-level risk factors, such as age, which may affect the risk of both events. Also correlations may be induced through the common treatment mechanism. Moreover, at the data level, there might be patients that experience both events.

In that case, we could change the harm function to account for this possibility, i.e.,

$$H_{early}=1\times p_{early}^{only stroke}+1\times p_{early}^{only haemorrhage}+2\times p_{early}^{stroke \& haemorrhage}$$

and likewise for late:

$$H_{late}=1\times p_{late}^{only stroke}+1\times p_{late}^{only haemorrhage}+2\times p_{late}^{stroke \& haemorrhage}$$

where we assumed double severity weight for the combined outcome.

To estimate all corresponding risks while accounting for the correlations between outcomes, we would need access to cross-tabulated data about the number of events (i.e., events of stroke only, haemorrhage only, and number of patients experiencing both or no events, per treatment group). We would then model this data using a multinomial likelihood for each treatment arm, with e.g. a flat Dirichlet prior on the four probabilities (i.e., no event, only stroke, only haemorrhage, both events). However, given that the trial did not provide this information, for illustrative reasons we only present the simple analysis described above. Alternatively, copulas could be used.

## 4.8 Results

*eTable 3: Probabilities that early intervention with anticoagulants in stroke patients with atrial fibrillation reduces the risk outcome of clinical outcomes as compared to late, and corresponding risk difference [95% Credible Interval], late minus early. RD>0 means increased risk with late intervention*

|  | **Probability that early is better than late intervention**  **risk difference [95% CrI]** | |
| --- | --- | --- |
| **Outcome** | **30 days** | **90 days** |
| **Composite outcome** | 93%  $RD=1.2\% [-0.4\%, +2.8\%]$ | 97%  $RD=1.8\% [0.0\%, +3.6\%]$ |
| **Recurrent ischemic stroke** | 96%  $RD=1.1\% [-0.1\%, +2.3\%]$ | 96%  $RD=1.2\% [-0.1\%, +2.6\%]$ |
| **Symptomatic intracranial haemorrhage** | 50%  $RD=0.0\% [-0.4\%, +0.4\%]$ | 49%  $RD=0.0\% [-0.4\%, +0.4\%]$ |
| **Systemic embolism** | 91%  $RD=0.5\% [-0.2\%, +1.2\%]$ | 94%  $RD=0.6\% [-0.1\%, +1.4\%]$ |
| **Major extracranial bleeding** | 74%  $RD=0.2\% [-0.4\%, +0.8\%]$ | 93%  $RD=0.5\% [-0.2\%, +1.2\%]$ |
| **Vascular death** | 41%  $RD=-0.1\% [-1.0\%, +0.8\%]$ | 44%  $RD=-0.1\% [-1.3\%, +1.1\%]$ |

eFigure 2: Posterior distributions of risk difference (RD) for early vs. late at 30 days. RD>0 (red colors) favors early intervention

eFigure 3: Posterior distributions of risk difference (RD) for early vs. late at 90 days. RD>0 (red colors) favors early intervention

eFigure 4: results of the sensitivity analysis, where we used a family of priors defined by a continuous parameter on the posterior $p({RD}^{haemorrhage}<-0.5\%)$.

Below we show results from the posterior predictive checking analysis for the outcome of symptomatic intracranial haemorrhage.

We found no indication of poor fit for any of the three models.

# Section 5. Details of the web application

The web application is based on R shiny^10^ and is freely available here: <https://oefthimiou.shinyapps.io/BayesianProbabilities/>. The code of the app is also available in our online appendix and can be used to run the app locally.

The app can be used to analyse either raw data (for a binary outcome), or a previously obtained estimate of a treatment effect and the corresponding 95% uncertainty interval. Below we discuss these in more detail.

## 5.1 Analysis of raw data (raw binary data)

The required input is the number of events and total randomized per treatment group ($f_{control}$, $f_{experimental},N_{control}$, $N_{experimental}$). The user needs to choose the effect measure among odds ratio (OR), risk ratio (RR), or risk difference (RD). The user also needs to provide a reference effect size, the Smallest Worthwhile Effect (SWE)”, which will be used to make probabilistic statements (i.e., there is XXX% probability that the treatment effect is larger than the alternative effect). As a default, the SWE is set to the null (i.e. 1, for OR/RR, and 0 for RD).

The web app then implements the following Bayesian model

Likelihood:

$$f_{control}\sim Binomial\left( p_{control}, N_{control} \right)$$

$$f_{experimental}\sim Binomial\left( p_{experimental}, N_{experimental} \right)$$

Prior distributions:

$$p_{control},p_{experimental}\sim Beta(1,1)$$

The $Beta(1,1)$ distribution is equivalent to a $Uniform(0,1)$, i.e. it is flat on the probability space. It is chosen because it is a conjugate prior to the binomial. With this choice, the posterior distributions of the probabilities of an event in control and experimental, $p_{control},p_{experimental}$*,* are respectively

$$Beta\left( {1+f}_{control}, {1+N}_{control}-f_{control} \right)$$

$$Beta\left( {1+f}_{experimemtal}, {1+N}_{control}-f_{experimemtal} \right)$$

We draw 1,000,000 samples from these posterior distributions, and from these samples we calculate 1,000,000 effect sizes using the corresponding formula:

$$OR=\frac{\frac{p_{experimental}}{1-p_{experimental}}}{\frac{p_{control}}{1-p_{control}}}$$

$$RR=\frac{p_{experimental}}{p_{control}}$$

$$RD=p_{late}-p_{early}$$

Finally, we plot the posterior distribution of the effect and calculate the percent of samples where the effect was larger than SWE.

## 5.2 Analysis of a generic estimate of treatment effect

For this analysis, the user needs to input a previously estimated treatment effect and the corresponding 95% confidence or credible interval. The user also needs to select the effect measure: odds ratio, risk ratio, risk difference (for binary outcomes), hazard ratio (for time-to-event outcomes), or mean difference (for continuous outcomes).

For the analysis, we make the assumption of normality about the previously estimated treatment effect: if the estimate was obtained from a Bayesian analysis, we assume the posterior to be a normal distribution; if the estimate was derived from a frequent analysis, we assume approximate normality of the likelihood function around the maximum likelihood estimate.

Based on these assumptions we fit the following model:

Likelihood:

$$TE\sim N\left( \mu, \sigma^{2}=seTE^{2} \right)$$

Prior distribution:

$$\mu\sim N(0, \sigma^{2}=1,000,00)$$

where $TE$ is the user-provided treatment effect, and $seTE$ is either the frequentist standard error (calculated from the user-provided 95% CI) or the Bayesian standard deviation of the posterior estimate of the treatment effect (calculated from the user-provided 95% CrI). Given this model, and the fact that the prior distribution is uninformative ($\sigma^{2}$ can be set to be arbitrarily large), the posterior distribution of the treatment effect ($\mu$) equals the likelihood.

We then draw 1,000,000 samples from the posterior of $\mu$. Depending on the user-provided effect measure, we either exponentiate the samples (to return to the natural scale, for the case when the effect measure is a ratio), or we use the samples as they are to produce plots of the posterior. We also calculate the percent of samples that were larger than the SWE, to provide probabilistic statements to the user.

# Section 6. Detailed results from analyses on negative trials

Below we present results from the main analysis. The sensitivity analysis with different choices for the prior distribution led to largely similar results.


## 6.1 Relative outcomes (ratios)

The **eTable 4** below shows the results from the Bayesian re-analyses of the estimated effects of negative trials that reported treatment effects as ratios (odds/risk/incidence rate/hazard ratios). The five columns on the right show the posterior probabilities that the treatment effect size (ES) is below 0.80; below 0.90; below 0.95; below 1.00. Smaller ES favors treatment. P(ES<1.00) corresponds to the Bayesian posterior probability that the experimental treatment is better than the control. We see that although all estimates were statistically non-significant, for several there was a high chance that the experimental treatment is better than the control. In several cases there was a high chance that the effect was quite pronounced, e.g. <0.80.

eTable 4: Results from analyses of negative studies using ratio as effect measure (odds ratio, risk ratio, incidence rate ratio, hazard ratio, geometric mean ratio). ES: effect size. P(ES<0.80) denotes the posterior probability that the effect size is <0.80. CrI: credible interval. HR: Hazard ratio. GMR: geometric mean ratio. OR: risk ratio. RR: risk ratio. IRR: incidence rate ratio.

| **Reference** | **Measure** | **Estimated Effect size [95% CrI]** | $\boldsymbol{P(ES<0.80)}$ | $\boldsymbol{P(ES<0.90)}$ | $\boldsymbol{P(ES<0.95)}$ | $\boldsymbol{P(ES<1.00)}$ |
| --- | --- | --- | --- | --- | --- | --- |
| JAMA. 2021;325(11):1061-1073 | HR | 1.09 [0.96;1.24] | $0\%$ | $0.1\%$ | $1.7\%$ | $9.3\%$ |
| JAMA. 2021;325(11):1061-1073 | HR | 1.09 [0.96;1.25] | $0\%$ | $0.2\%$ | $2.1\%$ | $10\%$ |
| JAMA. 2021;325(11):1061-1073 | HR | 0.92 [0.81;1.04] | $1.7\%$ | $38.3\%$ | $70.3\%$ | $90.7\%$ |
| JAMA. 2021;325(11):1061-1073 | HR | 0.92 [0.8;1.04] | $2.1\%$ | $39\%$ | $69.7\%$ | $89.8\%$ |
| JAMA. 2021;325(9):833-842 | HR | 0.7 [0.47;1.03] | $74.8\%$ | $89.6\%$ | $93.6\%$ | $96.3\%$ |
| JAMA. 2021;325(15):1513-1523 | GMR | 1.2 [1;1.5] | $0\%$ | $0.3\%$ | $1.3\%$ | $4.1\%$ |
| JAMA. 2021;326(24):2478-2487 | RR | 0.87 [0.74;1.03] | $16.1\%$ | $65.5\%$ | $85.2\%$ | $95.1\%$ |
| JAMA. 2021;326(4):314-323 | HR | 0.92 [0.81;1.05] | $1.8\%$ | $37.1\%$ | $68.5\%$ | $89.7\%$ |
| JAMA. 2021;326(4):314-323 | OR | 0.94 [0.83;1.08] | $0.7\%$ | $24.6\%$ | $54.6\%$ | $80.8\%$ |
| JAMA. 2021;326(11):1024-1033 | HR | 1.03 [0.87;1.22] | $0.2\%$ | $5.8\%$ | $17.3\%$ | $36.9\%$ |
| JAMA. 2021;326(12):1158-1169 | OR | 0.75 [0.5;1.14] | $61.7\%$ | $80.6\%$ | $86.9\%$ | $91.4\%$ |
| JAMA. 2021;326(15):1494-1503 | RR | 1.07 [0.86;1.33] | $0.4\%$ | $6.1\%$ | $14.3\%$ | $27.3\%$ |
| JAMA. 2021;325(3):254-264 | ratio | 1.05 [0.99;1.11] | $0\%$ | $0\%$ | $0\%$ | $4.1\%$ |
| JAMA. 2021;325(3):254-264 | ratio | 0.95 [0.9;1.01] | $0\%$ | $3.3\%$ | $49.7\%$ | $95.9\%$ |
| JAMA. 2021;325(14):1426-1435 | HR | 0.93 [0.76;1.15] | $7.2\%$ | $36.2\%$ | $56.1\%$ | $73.7\%$ |
| JAMA. 2021;325(18):1841-1851 | HR | 1.04 [0.71;1.53] | $9.2\%$ | $23.4\%$ | $32.6\%$ | $42.4\%$ |
| JAMA. 2021;326(11):1013-1023 | RR | 1.05 [0.83;1.33] | $1.2\%$ | $10\%$ | $20\%$ | $34\%$ |
| JAMA. 2021;325(13):1277-1285 | HR | 0.89 [0.76;1.03] | $8.5\%$ | $55.6\%$ | $79.9\%$ | $93.3\%$ |
| JAMA. 2021;325(19):1946-1954 | HR | 1.11 [0.88;1.41] | $0.4\%$ | $4.2\%$ | $9.9\%$ | $19.4\%$ |
| JAMA. 2021;325(20):2056-2066 | OR | 0.98 [0.68;1.41] | $13.7\%$ | $32.3\%$ | $43.3\%$ | $54.3\%$ |
| JAMA. 2021;326(22):2268-2276 | RR | 1.39 [0.97;2.04] | $0.2\%$ | $1.1\%$ | $2.2\%$ | $4.1\%$ |
| JAMA. 2021;325(1):39-49 | HR | 1.06 [0.98;1.17] | $0\%$ | $0\%$ | $0.9\%$ | $10.3\%$ |
| JAMA. 2021;326(9):818-829 | HR | 0.97 [0.9;1.05] | $0\%$ | $2.8\%$ | $29.4\%$ | $77.8\%$ |
| JAMA. 2021;326(9):830-838 | HR | 1.03 [0.96;1.11] | $0\%$ | $0\%$ | $1.4\%$ | $21.5\%$ |
| N Engl J Med 2021; 384:905-914 | HR | 0.94 [0.68;1.3] | $15.8\%$ | $38.8\%$ | $51.8\%$ | $63.9\%$ |
| N Engl J Med 2021;384:808-17 | OR | 0.98 [0.71;1.33] | $10\%$ | $29.6\%$ | $42.4\%$ | $55.3\%$ |
| N Engl J Med 2021; 384:1003-1014 | HR | 0.73 [0.48;1.12] | $65.6\%$ | $82.9\%$ | $88.4\%$ | $92.3\%$ |
| N Engl J Med 2021; 384:1003-1014 | HR | 0.91 [0.57;1.45] | $29.2\%$ | $48.2\%$ | $57.1\%$ | $65.3\%$ |
| N Engl J Med 2021;384:2283-94 | RR | 1.04 [0.94;1.14] | $0\%$ | $0.1\%$ | $3.3\%$ | $21.5\%$ |
| N Engl J Med 2021;385:119-29 | RR | 0.79 [0.61;1.01] | $54.8\%$ | $85.2\%$ | $93\%$ | $97\%$ |
| N Engl J Med 2021;385:119-29 | RR | 0.81 [0.61;1.08] | $45.8\%$ | $75.9\%$ | $85.9\%$ | $92.3\%$ |
| N Engl J Med 2021;385:2544-53 | HR | 1.28 [1;1.63] | $0\%$ | $0.2\%$ | $0.8\%$ | $2.3\%$ |
| N Engl J Med 2021;385:395-405 | HR | 0.99 [0.85;1.15] | $0.3\%$ | $10.8\%$ | $29.3\%$ | $55.1\%$ |
| N Engl J Med 2021;384:1089-100 | HR | 1.02 [0.81;1.3] | $2.3\%$ | $15\%$ | $27.9\%$ | $43.6\%$ |
| N Engl J Med 2021; 384:895-904 | RR | 0.95 [0.87;1.05] | $0\%$ | $13\%$ | $50.1\%$ | $85.5\%$ |
| N Engl J Med 2021; 384:895-904 | RR | 0.98 [0.93;1.02] | $0\%$ | $0\%$ | $9.3\%$ | $80.4\%$ |
| N Engl J Med 2021; 384:895-904 | RR | 1.04 [0.88;1.23] | $0.1\%$ | $4.4\%$ | $14.3\%$ | $32.3\%$ |
| N Engl J Med 2021; 384:895-904 | RR | 1 [0.93;1.08] | $0\%$ | $0.3\%$ | $8.6\%$ | $49.8\%$ |
| N Engl J Med 2021;384:1789-99 | HR | 0.97 [0.84;1.12] | $0.4\%$ | $15.4\%$ | $39\%$ | $66.2\%$ |
| N Engl J Med 2021;385:436-44 | RR | 1.17 [0.8;1.72] | $2.6\%$ | $8.7\%$ | $14.1\%$ | $21\%$ |
| N Engl J Med 2021; 384:1981-1990 | HR | 1.02 [0.91;1.14] | $0\%$ | $1.4\%$ | $10.9\%$ | $36.7\%$ |
| N Engl J Med 2021;384:1910-20 | RR | 0.85 [0.67;1.09] | $31.9\%$ | $68.4\%$ | $82.1\%$ | $90.8\%$ |
| N Engl J Med 2021;385:1833-44 | OR | 0.84 [0.62;1.15] | $38\%$ | $66.9\%$ | $78.1\%$ | $86.5\%$ |
| N Engl J Med 2021; 384:1885-1898 | RR | 0.78 [0.4;1.5] | $52.7\%$ | $66.2\%$ | $72\%$ | $76.8\%$ |
| N Engl J Med 2021;385:516-25 | RR | 0.9 [0.69;1.19] | $19.8\%$ | $49.9\%$ | $65.1\%$ | $77.5\%$ |
| N Engl J Med 2021;384:417-27 | RR | 0.86 [0.52;1.42] | $39.2\%$ | $57.4\%$ | $65.5\%$ | $72.6\%$ |
| N Engl J Med 2021;385:2025-35 | RR | 1.03 [0.84;1.27] | $0.8\%$ | $10.2\%$ | $22.2\%$ | $39.2\%$ |
| N Engl J Med 2021;385:1845-55 | HR | 0.9 [0.78;1.04] | $5.3\%$ | $49.6\%$ | $76.8\%$ | $92.3\%$ |
| N Engl J Med 2021;385:297-308 | HR | 1.32 [0.78;2.23] | $3.1\%$ | $7.7\%$ | $10.9\%$ | $14.8\%$ |
| N Engl J Med 2021;384:1301-11 | RR | 1.02 [0.94;1.11] | $0\%$ | $0.1\%$ | $4.7\%$ | $32.2\%$ |
| N Engl J Med 2021;384:619-29 | OR | 1.2 [0.74;1.92] | $4.7\%$ | $11.7\%$ | $16.5\%$ | $22.1\%$ |
| N Engl J Med 2021;384:2028-38 | RR | 0.77 [0.58;1.04] | $60.4\%$ | $85.2\%$ | $92.1\%$ | $95.9\%$ |
| N Engl J Med 2021; 384:216-228 | HR | 0.79 [0.63;1] | $54.1\%$ | $86.4\%$ | $94.2\%$ | $97.8\%$ |
| N Engl J Med 2021; 384:216-228 | HR | 0.86 [0.67;1.1] | $28.1\%$ | $63.6\%$ | $78.2\%$ | $88.4\%$ |
| Lancet 2021; 398: 991–1001 | HR | 0.88 [0.74;1.05] | $14.2\%$ | $59.8\%$ | $80.6\%$ | $92.5\%$ |
| Lancet 2021; 398: 1417–26 | HR | 0.88 [0.7;1.11] | $19.1\%$ | $56.1\%$ | $72.9\%$ | $85.2\%$ |
| Lancet 2021; 397: 2182–93 | HR | 0.94 [0.82;1.08] | $1\%$ | $26.5\%$ | $56.3\%$ | $81.2\%$ |
| Lancet 2021; 397: 2182–93 | HR | 0.96 [0.83;1.1] | $0.6\%$ | $18.4\%$ | $44.1\%$ | $71.4\%$ |
| Lancet 2021; 398: 1687–99 | RR | 0.91 [0.81;1.02] | $1.4\%$ | $42.6\%$ | $77.1\%$ | $94.7\%$ |
| Lancet 2021; 398: 1687–99 | RR | 0.9 [0.77;1.06] | $7.5\%$ | $50.2\%$ | $74.4\%$ | $90.3\%$ |
| Lancet 2021; 398: 1687–99 | RR | 0.97 [0.82;1.14] | $1\%$ | $18.6\%$ | $40.3\%$ | $64.4\%$ |
| Lancet 2021; 398: 1687–99 | RR | 0.98 [0.87;1.1] | $0\%$ | $7.6\%$ | $30.3\%$ | $63.3\%$ |
| Lancet 2021; 397: 112–18 | OR | 1.16 [0.89;1.52] | $0.3\%$ | $2.9\%$ | $6.8\%$ | $13.3\%$ |
| Lancet 2021; 397: 605–12 | HR | 0.97 [0.87;1.07] | $0\%$ | $7.5\%$ | $34.3\%$ | $71.7\%$ |
| Lancet 2021; 397: 2049–59 | HR | 1 [0.93;1.07] | $0\%$ | $0.2\%$ | $7.6\%$ | $49.9\%$ |
| Lancet 2021; 398: 1507–16 | HR | 0.8 [0.61;1.05] | $50.2\%$ | $80.5\%$ | $89.3\%$ | $94.7\%$ |
| BMJ 2021;373:n1162 | OR | 0.85 [0.6;1.08] | $34.4\%$ | $64.7\%$ | $77.1\%$ | $86.2\%$ |
| BMJ 2021;374:n1585 | HR | 0.95 [0.77;1.17] | $5.3\%$ | $31\%$ | $50.6\%$ | $68.9\%$ |
| BMJ 2021;372:n716 | RR | 1.17 [0.9;1.53] | $0.3\%$ | $2.7\%$ | $6.2\%$ | $12.4\%$ |
| BMJ 2021;374:n2103 | GMR | 0.72 [0.51;1.01] | $72.7\%$ | $90.1\%$ | $94.5\%$ | $97.3\%$ |
| BMJ 2021;372:n740 | IRR | 0.82 [0.66;1.02] | $41.6\%$ | $80.1\%$ | $90.7\%$ | $96.2\%$ |
| BMJ 2021;373:n893 | RR | 0.76 [0.57;1.01] | $63.8\%$ | $87.6\%$ | $93.7\%$ | $97\%$ |
| BMJ 2021;374:n2106 | HR | 0.91 [0.62;1.35] | $25.9\%$ | $48\%$ | $58.6\%$ | $68.4\%$ |
| BMJ 2021;374:n2106 | HR | 0.91 [0.62;1.35] | $25.6\%$ | $47.4\%$ | $58.2\%$ | $67.9\%$ |
| BMJ 2021;374:n1857 | RR | 1.03 [0.89;1.21] | $0.1\%$ | $4.2\%$ | $14.9\%$ | $35.3\%$ |
| BMJ 2021;374:n1857 | RR | 1.01 [0.76;1.36] | $5.8\%$ | $21.5\%$ | $34\%$ | $47.3\%$ |
| BMJ 2021;372:n256 | HR | 0.85 [0.71;1.01] | $24.7\%$ | $73.7\%$ | $89.1\%$ | $96.2\%$ |
| BMJ 2021;375:n2400 | OR | 0.69 [0.43;1.1] | $73.5\%$ | $86.6\%$ | $90.9\%$ | $93.9\%$ |
| BMJ 2021;372:n355 | OR | 0.97 [0.86;1.08] | $0\%$ | $10.1\%$ | $35.9\%$ | $69.9\%$ |
| BMJ 2021;372:n84 | OR | 1.54 [0.66;3.66] | $6.5\%$ | $10.7\%$ | $13.2\%$ | $15.9\%$ |
| BMJ 2021;372:m4786 | RR | 1.02 [0.93;1.13] | $0\%$ | $0.6\%$ | $7.5\%$ | $34.7\%$ |
| Ann Intern Med. 2021;174:145–156 | HR | 0.94 [0.76;1.15] | $6.3\%$ | $33.9\%$ | $53.5\%$ | $71.9\%$ |
| Ann Intern Med. 2021;174:344–352 | HR | 1.1 [0.63;1.66] | $9.9\%$ | $20.8\%$ | $27.7\%$ | $35.1\%$ |
| Ann Intern Med. 2021;174:335–343 | OR | 0.82 [0.54;1.33] | $45.7\%$ | $66\%$ | $74.4\%$ | $80.9\%$ |
| Ann Intern Med. 2021;174:1367-1376 | HR | 0.96 [0.61;1.5] | $21.3\%$ | $38.9\%$ | $48\%$ | $56.9\%$ |
| Ann Intern Med. 2021;174:889–898 | RR | 0.95 [0.87;1.05] | $0\%$ | $12.2\%$ | $47.7\%$ | $83.9\%$ |
| Ann Intern Med. 2021;174:1683-1692 | OR | 0.9 [0.67;1.2] | $21.3\%$ | $49.6\%$ | $63.7\%$ | $75.6\%$ |
| PLoS Med 18(9): e1003744 | RR | 1.08 [0.99;1.16] | $0\%$ | $0\%$ | $0.1\%$ | $3.9\%$ |
| PLoS Med 18(9): e1003744 | RR | 1.11 [0.96;1.3] | $0\%$ | $0.3\%$ | $2\%$ | $8.3\%$ |
| PLoS Med 18(5): e1003628 | OR | 0.96 [0.63;1.47] | $19.4\%$ | $37.7\%$ | $47.5\%$ | $56.9\%$ |
| PLoS Med 18(6): e1003670 | OR | 1.09 [0.75;1.82] | $8.9\%$ | $20.4\%$ | $27.7\%$ | $35.8\%$ |
| PLoS Med 18(5): e1003630 | OR | 1.15 [0.67;2] | $9.6\%$ | $18.9\%$ | $24.5\%$ | $30.7\%$ |
| PLoS Med 18(1): e1003475 | RR | 0.96 [0.89;1.02] | $0\%$ | $2.7\%$ | $36.1\%$ | $87.2\%$ |
| PLoS Med 18(1): e1003475 | RR | 0.95 [0.88;1.04] | $0\%$ | $10\%$ | $47.7\%$ | $86.7\%$ |
| PLoS Med 18(10): e1003808 | RR | 0.91 [0.8;1.02] | $1.8\%$ | $42.6\%$ | $75.8\%$ | $93.4\%$ |
| PLoS Med 18(1): e1003411 | RR | 1.1 [0.86;1.42] | $0.6\%$ | $6\%$ | $12.9\%$ | $23.3\%$ |
| PLoS Med 18(5): e1003650 | RR | 1.57 [0.78;3.15] | $3\%$ | $6.1\%$ | $8.1\%$ | $10.4\%$ |
| PLoS Med 18(5): e1003650 | HR | 0.87 [0.56;1.34] | $35.6\%$ | $56.2\%$ | $65.3\%$ | $73.2\%$ |
| PLoS Med 18(5): e1003650 | HR | 1.11 [0.78;1.58] | $3.5\%$ | $12.6\%$ | $19.6\%$ | $28.3\%$ |
| PLoS Med 18(5): e1003650 | HR | 1.09 [0.73;1.65] | $7\%$ | $17.9\%$ | $25.8\%$ | $34\%$ |
| PLoS Med 18(5): e1003650 | HR | 1.08 [0.55;1.54] | $12.6\%$ | $24.6\%$ | $31.7\%$ | $38.9\%$ |
| PLoS Med 18(5): e1003650 | RR | 0.89 [0.4;1.98] | $39.8\%$ | $51.1\%$ | $56.6\%$ | $61.6\%$ |
| PLoS Med 18(4): e1003609 | RRR | 0.96 [0.76;1.2] | $5.8\%$ | $29.4\%$ | $47\%$ | $64.6\%$ |
| PLoS Med 18(9): e1003680 | OR | 0.96 [0.84;1.11] | $0.5\%$ | $18\%$ | $44.1\%$ | $71.8\%$ |
| PLoS Med 18(9): e1003680 | OR | 1.02 [0.86;1.2] | $0.3\%$ | $7.3\%$ | $20\%$ | $41.1\%$ |
| PLoS Med 18(3): e1003506 | OR | 0.86 [0.55;1.36] | $37.7\%$ | $58.4\%$ | $67\%$ | $74.5\%$ |
| PLoS Med 18(3): e1003506 | OR | 0.87 [0.55;1.38] | $36.5\%$ | $56.1\%$ | $65\%$ | $72.9\%$ |
| PLoS Med 18(5): e1003601 | RR | 0.92 [0.6;1.42] | $26.4\%$ | $46\%$ | $56\%$ | $64.8\%$ |
| PLoS Med 18(6): e1003668 | HR | 0.87 [0.52;1.45] | $37\%$ | $55\%$ | $63.2\%$ | $70.4\%$ |
| PLoS Med 18(10): e1003798 | OR | 0.68 [0.36;1.28] | $69.4\%$ | $80.8\%$ | $85\%$ | $88.4\%$ |
| PLoS Med 18(10): e1003798 | OR | 0.72 [0.38;1.39] | $61.8\%$ | $74.3\%$ | $79.1\%$ | $83.4\%$ |
| PLoS Med 18(7): e1003689 | OR | 0.97 [0.66;1.42] | $16\%$ | $35.1\%$ | $45.9\%$ | $56.4\%$ |
| PLoS Med 18(7): e1003694 | IRR | 0.9 [0.74;1.08] | $11.3\%$ | $50.1\%$ | $71.3\%$ | $86.1\%$ |

Based on the results of this table we create **eFigure 5** shown below.

eFigure 5: Histogram of the posterior probabilities of treatment effects obtained from 113 estimates from published, negative RCTs. Smaller ratios favor treatment. The four panels show the results of the same analyses. Top left panel: posterior probabilities that the experimental treatment is better than the control (ratio<1). Top right: posterior probabilities that the ratio is <0.95 (top right). Bottom left: ratio <0.90. Bottom right: ratio <0.80. Green colors indicate high posterior probability (also shown in the x-axis) that the ratio is below the threshold shown in the title of the plot. The plots show how negative trials may yield evidence of treatment effect.

Below we show the results from the first sensitivity analysis, where we used a prior $\mu_{i}\sim N(0, \sigma^{2}=10)$ for the effect (log of the ratio). Results were very similar to the primary analysis. For 23/113 of the outcomes there was >90% probability that the treatment is superior to control. For 9/113, this probability was >95%.

eTable 5: Results from analyses of negative studies using ratio as effect measure (odds ratio, risk ratio, incidence rate ratio, hazard ratio, geometric mean ratio), with prior for the log ratio $\mu_{i}\sim N(0, \sigma^{2}=10)$. ES: effect size. P(ES<0.80) denotes the posterior probability that the effect size is <0.80. CrI: credible interval. HR: Hazard ratio. GMR: geometric mean ratio. OR: risk ratio. RR: risk ratio. IRR: incidence rate ratio.

| **Study** | **Measure** | **Estimated** | **P(ES<0.80)** | **P(ES<0.90)** | **P(ES<0.95)** | **P(ES<1.00)** |
| --- | --- | --- | --- | --- | --- | --- |
| JAMA. 2021;325(11):1061-1073 | HR | 1.09 [0.96;1.24] | 0% | 0.2% | 1.7% | 9.1% |
| JAMA. 2021;325(11):1061-1073 | HR | 1.09 [0.96;1.24] | 0% | 0.2% | 2% | 10.1% |
| JAMA. 2021;325(11):1061-1073 | HR | 0.92 [0.81;1.04] | 1.8% | 38.6% | 70.2% | 90.5% |
| JAMA. 2021;325(11):1061-1073 | HR | 0.92 [0.8;1.05] | 2% | 38.8% | 70% | 90% |
| JAMA. 2021;325(9):833-842 | HR | 0.7 [0.48;1.04] | 74.7% | 89.5% | 93.5% | 96.2% |
| JAMA. 2021;325(15):1513-1523 | GMR | 1.2 [0.98;1.47] | 0% | 0.3% | 1.3% | 4.1% |
| JAMA. 2021;326(24):2478-2487 | RR | 0.87 [0.74;1.03] | 16% | 65.3% | 84.9% | 94.9% |
| JAMA. 2021;326(4):314-323 | HR | 0.92 [0.81;1.05] | 1.8% | 37.1% | 68.4% | 89.4% |
| JAMA. 2021;326(4):314-323 | OR | 0.94 [0.83;1.08] | 0.8% | 24% | 53.7% | 80.6% |
| JAMA. 2021;326(11):1024-1033 | HR | 1.03 [0.87;1.22] | 0.2% | 6% | 17.8% | 37% |
| JAMA. 2021;326(12):1158-1169 | OR | 0.75 [0.5;1.13] | 61.7% | 80.8% | 87.1% | 91.6% |
| JAMA. 2021;326(15):1494-1503 | RR | 1.07 [0.86;1.33] | 0.4% | 5.9% | 14.2% | 27% |
| JAMA. 2021;325(3):254-264 | ratio | 1.05 [0.99;1.12] | 0% | 0% | 0% | 4.1% |
| JAMA. 2021;325(3):254-264 | ratio | 0.95 [0.9;1.01] | 0% | 3.4% | 49.8% | 96% |
| JAMA. 2021;325(14):1426-1435 | HR | 0.93 [0.76;1.15] | 7.1% | 36.6% | 56.3% | 73.8% |
| JAMA. 2021;325(18):1841-1851 | HR | 1.04 [0.71;1.54] | 9.2% | 23% | 32.2% | 42% |
| JAMA. 2021;326(11):1013-1023 | RR | 1.05 [0.83;1.33] | 1.2% | 10.3% | 20.1% | 34% |
| JAMA. 2021;325(13):1277-1285 | HR | 0.89 [0.76;1.04] | 8.4% | 55.7% | 80.1% | 93.4% |
| JAMA. 2021;325(19):1946-1954 | HR | 1.11 [0.88;1.4] | 0.3% | 4% | 9.6% | 19.2% |
| JAMA. 2021;325(20):2056-2066 | OR | 0.98 [0.68;1.41] | 13.4% | 32.1% | 43.1% | 54.1% |
| JAMA. 2021;326(22):2268-2276 | RR | 1.39 [0.96;2.01] | 0.2% | 1.1% | 2.2% | 4.2% |
| JAMA. 2021;325(1):39-49 | HR | 1.06 [0.97;1.16] | 0% | 0% | 0.9% | 10.4% |
| JAMA. 2021;326(9):818-829 | HR | 0.97 [0.9;1.05] | 0% | 2.9% | 29.2% | 78.1% |
| JAMA. 2021;326(9):830-838 | HR | 1.03 [0.96;1.11] | 0% | 0% | 1.4% | 21.5% |
| N Engl J Med 2021; 384:905-914 | HR | 0.94 [0.68;1.3] | 15.8% | 38.5% | 51.5% | 64% |
| N Engl J Med 2021;384:808-17 | OR | 0.98 [0.71;1.35] | 10.2% | 29.5% | 42.3% | 54.9% |
| N Engl J Med 2021; 384:1003-1014 | HR | 0.74 [0.48;1.12] | 65.5% | 83% | 88.7% | 92.6% |
| N Engl J Med 2021; 384:1003-1014 | HR | 0.91 [0.57;1.45] | 28.8% | 47.6% | 57% | 65.4% |
| N Engl J Med 2021;384:2283-94 | RR | 1.04 [0.94;1.15] | 0% | 0.2% | 3.3% | 21.6% |
| N Engl J Med 2021;385:119-29 | RR | 0.79 [0.61;1.01] | 55.1% | 85.3% | 92.9% | 97% |
| N Engl J Med 2021;385:119-29 | RR | 0.81 [0.61;1.09] | 45.8% | 75.7% | 85.7% | 92.2% |
| N Engl J Med 2021;385:2544-53 | HR | 1.28 [1;1.64] | 0% | 0.3% | 0.8% | 2.4% |
| N Engl J Med 2021;385:395-405 | HR | 0.99 [0.85;1.15] | 0.2% | 10.5% | 29.3% | 54.8% |
| N Engl J Med 2021;384:1089-100 | HR | 1.02 [0.8;1.29] | 2.2% | 15% | 27.9% | 43.5% |
| N Engl J Med 2021; 384:895-904 | RR | 0.95 [0.86;1.04] | 0% | 13.3% | 50.3% | 86% |
| N Engl J Med 2021; 384:895-904 | RR | 0.98 [0.94;1.03] | 0% | 0% | 9.4% | 80.4% |
| N Engl J Med 2021; 384:895-904 | RR | 1.04 [0.88;1.23] | 0.1% | 4.5% | 14.4% | 32.4% |
| N Engl J Med 2021; 384:895-904 | RR | 1 [0.93;1.08] | 0% | 0.3% | 8.8% | 50.2% |
| N Engl J Med 2021;384:1789-99 | HR | 0.97 [0.84;1.12] | 0.4% | 15.4% | 38.9% | 66% |
| N Engl J Med 2021;385:436-44 | RR | 1.17 [0.8;1.7] | 2.6% | 9% | 14.3% | 21.2% |
| N Engl J Med 2021; 384:1981-1990 | HR | 1.02 [0.91;1.14] | 0% | 1.5% | 10.9% | 36.4% |
| N Engl J Med 2021;384:1910-20 | RR | 0.85 [0.66;1.08] | 32.2% | 68.9% | 82.5% | 91% |
| N Engl J Med 2021;385:1833-44 | OR | 0.84 [0.62;1.14] | 37.3% | 66.9% | 78.3% | 86.6% |
| N Engl J Med 2021; 384:1885-1898 | RR | 0.78 [0.41;1.5] | 52.4% | 66.2% | 72.2% | 77.2% |
| N Engl J Med 2021;385:516-25 | RR | 0.9 [0.68;1.18] | 19.8% | 50.3% | 65.2% | 77.5% |
| N Engl J Med 2021;384:417-27 | RR | 0.86 [0.52;1.42] | 38.6% | 56.6% | 64.8% | 72% |
| N Engl J Med 2021;385:2025-35 | RR | 1.03 [0.84;1.27] | 0.8% | 10.5% | 22.8% | 39.4% |
| N Engl J Med 2021;385:1845-55 | HR | 0.9 [0.78;1.04] | 5.4% | 50.7% | 77.3% | 92.5% |
| N Engl J Med 2021;385:297-308 | HR | 1.32 [0.78;2.22] | 3.2% | 7.7% | 11.1% | 15.2% |
| N Engl J Med 2021;384:1301-11 | RR | 1.02 [0.94;1.11] | 0% | 0.1% | 4.6% | 32.6% |
| N Engl J Med 2021;384:619-29 | OR | 1.2 [0.75;1.94] | 4.6% | 11.3% | 15.9% | 21.9% |
| N Engl J Med 2021;384:2028-38 | RR | 0.77 [0.57;1.03] | 59.8% | 85% | 91.9% | 95.9% |
| N Engl J Med 2021; 384:216-228 | HR | 0.79 [0.63;1] | 54.3% | 86.3% | 93.9% | 97.6% |
| N Engl J Med 2021; 384:216-228 | HR | 0.86 [0.67;1.1] | 28.3% | 63.9% | 78.5% | 88.4% |
| Lancet 2021; 398: 991–1001 | HR | 0.88 [0.74;1.05] | 14% | 60% | 80.5% | 92.5% |
| Lancet 2021; 398: 1417–26 | HR | 0.88 [0.7;1.11] | 19.2% | 55.9% | 73.1% | 85.5% |
| Lancet 2021; 397: 2182–93 | HR | 0.94 [0.82;1.08] | 1.2% | 26.7% | 56.3% | 81.3% |
| Lancet 2021; 397: 2182–93 | HR | 0.96 [0.84;1.1] | 0.6% | 18.3% | 44.5% | 71.8% |
| Lancet 2021; 398: 1687–99 | RR | 0.91 [0.81;1.02] | 1.4% | 42.4% | 76.8% | 94.5% |
| Lancet 2021; 398: 1687–99 | RR | 0.9 [0.77;1.06] | 7.2% | 50.1% | 74.9% | 90.1% |
| Lancet 2021; 398: 1687–99 | RR | 0.97 [0.82;1.14] | 1% | 18.4% | 40.3% | 64.4% |
| Lancet 2021; 398: 1687–99 | RR | 0.98 [0.87;1.1] | 0% | 7.8% | 30.2% | 63.6% |
| Lancet 2021; 397: 112–18 | OR | 1.16 [0.89;1.52] | 0.3% | 2.9% | 6.6% | 13.1% |
| Lancet 2021; 397: 605–12 | HR | 0.97 [0.87;1.08] | 0% | 7.7% | 34.5% | 72.2% |
| Lancet 2021; 397: 2049–59 | HR | 1 [0.93;1.07] | 0% | 0.2% | 7.4% | 49.8% |
| Lancet 2021; 398: 1507–16 | HR | 0.8 [0.61;1.05] | 49.5% | 79.8% | 89.1% | 94.5% |
| BMJ 2021;373:n1162 | OR | 0.85 [0.63;1.14] | 34.5% | 64.9% | 77% | 85.9% |
| BMJ 2021;374:n1585 | HR | 0.95 [0.77;1.17] | 5.2% | 30.2% | 49.7% | 68.4% |
| BMJ 2021;372:n716 | RR | 1.17 [0.9;1.52] | 0.2% | 2.6% | 6.1% | 12.2% |
| BMJ 2021;374:n2103 | GMR | 0.72 [0.51;1.01] | 72.5% | 90% | 94.5% | 97.1% |
| BMJ 2021;372:n740 | IRR | 0.82 [0.66;1.02] | 41.5% | 80.2% | 90.8% | 96.2% |
| BMJ 2021;373:n893 | RR | 0.76 [0.57;1.01] | 63.6% | 87.6% | 93.6% | 96.9% |
| BMJ 2021;374:n2106 | HR | 0.91 [0.62;1.34] | 25.8% | 48% | 58.7% | 68.1% |
| BMJ 2021;374:n2106 | HR | 0.91 [0.62;1.35] | 25.6% | 47.4% | 58.1% | 67.8% |
| BMJ 2021;374:n1857 | RR | 1.03 [0.88;1.2] | 0% | 4.3% | 14.9% | 35.4% |
| BMJ 2021;374:n1857 | RR | 1.01 [0.75;1.35] | 5.9% | 21.8% | 34.3% | 47.5% |
| BMJ 2021;372:n256 | HR | 0.85 [0.71;1.01] | 25.2% | 73.9% | 89.1% | 96.5% |
| BMJ 2021;375:n2400 | OR | 0.69 [0.44;1.11] | 72.9% | 86.4% | 90.8% | 93.9% |
| BMJ 2021;372:n355 | OR | 0.97 [0.87;1.09] | 0% | 9.6% | 35.6% | 69.9% |
| BMJ 2021;372:n84 | OR | 1.53 [0.66;3.56] | 6.7% | 11.1% | 13.6% | 16.3% |
| BMJ 2021;372:m4786 | RR | 1.02 [0.92;1.12] | 0% | 0.6% | 7.6% | 34.9% |
| Ann Intern Med. 2021;174:145–156 | HR | 0.94 [0.77;1.16] | 6.3% | 33.9% | 53.9% | 72% |
| Ann Intern Med. 2021;174:344–352 | HR | 1.1 [0.68;1.79] | 9.9% | 20.9% | 27.8% | 35.1% |
| Ann Intern Med. 2021;174:335–343 | OR | 0.82 [0.52;1.29] | 45.5% | 65.5% | 73.5% | 80.2% |
| Ann Intern Med. 2021;174:1367-1376 | HR | 0.96 [0.61;1.5] | 21.6% | 38.9% | 48.4% | 57.3% |
| Ann Intern Med. 2021;174:889–898 | RR | 0.95 [0.87;1.05] | 0% | 12.5% | 48.4% | 84.2% |
| Ann Intern Med. 2021;174:1683-1692 | OR | 0.9 [0.67;1.21] | 21% | 49.7% | 64% | 75.8% |
| PLoS Med 18(9): e1003744 | RR | 1.08 [0.99;1.16] | 0% | 0% | 0.1% | 3.8% |
| PLoS Med 18(9): e1003744 | RR | 1.11 [0.96;1.29] | 0% | 0.3% | 2% | 8.4% |
| PLoS Med 18(5): e1003628 | OR | 0.96 [0.63;1.46] | 19.6% | 38.1% | 47.9% | 57.4% |
| PLoS Med 18(6): e1003670 | OR | 1.09 [0.7;1.7] | 8.9% | 20.5% | 27.9% | 36.1% |
| PLoS Med 18(5): e1003630 | OR | 1.15 [0.67;1.98] | 9.7% | 19.1% | 24.6% | 30.7% |
| PLoS Med 18(1): e1003475 | RR | 0.96 [0.9;1.03] | 0% | 2.6% | 36.2% | 87.4% |
| PLoS Med 18(1): e1003475 | RR | 0.95 [0.87;1.04] | 0% | 9.9% | 47.8% | 86.6% |
| PLoS Med 18(10): e1003808 | RR | 0.91 [0.8;1.03] | 2% | 43.3% | 75.6% | 93.5% |
| PLoS Med 18(1): e1003411 | RR | 1.1 [0.86;1.41] | 0.6% | 5.8% | 12.5% | 22.8% |
| PLoS Med 18(5): e1003650 | RR | 1.57 [0.78;3.12] | 3% | 6% | 8.2% | 10.6% |
| PLoS Med 18(5): e1003650 | HR | 0.87 [0.57;1.35] | 35.1% | 55.7% | 65.1% | 73.1% |
| PLoS Med 18(5): e1003650 | HR | 1.11 [0.78;1.59] | 3.5% | 12.3% | 19.6% | 28.1% |
| PLoS Med 18(5): e1003650 | HR | 1.09 [0.72;1.63] | 6.8% | 18% | 25.7% | 34.3% |
| PLoS Med 18(5): e1003650 | HR | 1.08 [0.65;1.8] | 12.8% | 24.6% | 31.5% | 38.8% |
| PLoS Med 18(5): e1003650 | RR | 0.89 [0.4;1.97] | 39.8% | 51.3% | 56.9% | 61.7% |
| PLoS Med 18(4): e1003609 | RRR | 0.96 [0.77;1.2] | 5.8% | 29.2% | 47% | 64.6% |
| PLoS Med 18(9): e1003680 | OR | 0.96 [0.83;1.1] | 0.5% | 18.2% | 44.2% | 71.7% |
| PLoS Med 18(9): e1003680 | OR | 1.02 [0.86;1.21] | 0.2% | 7.1% | 20.1% | 40.7% |
| PLoS Med 18(3): e1003506 | OR | 0.86 [0.55;1.35] | 37.2% | 57.4% | 66.4% | 74% |
| PLoS Med 18(3): e1003506 | OR | 0.87 [0.55;1.38] | 36% | 55.8% | 64.7% | 72.4% |
| PLoS Med 18(5): e1003601 | RR | 0.92 [0.6;1.41] | 26.1% | 46.2% | 56.3% | 65.1% |
| PLoS Med 18(6): e1003668 | HR | 0.87 [0.53;1.45] | 37.1% | 55.2% | 63.1% | 70.2% |
| PLoS Med 18(10): e1003798 | OR | 0.69 [0.36;1.29] | 68.3% | 80.4% | 84.7% | 88.1% |
| PLoS Med 18(10): e1003798 | OR | 0.73 [0.38;1.38] | 61.4% | 74.1% | 79.2% | 83.4% |
| PLoS Med 18(7): e1003689 | OR | 0.97 [0.66;1.43] | 16% | 35% | 45.6% | 56.2% |
| PLoS Med 18(7): e1003694 | IRR | 0.9 [0.75;1.09] | 10.9% | 50.1% | 71% | 86.1% |

Below we show the results from the second sensitivity analysis, where we used a prior $\mu_{i}\sim N(0, \sigma^{2}=1/5)$ for the effect (log of the ratio). For 20/113 of the outcomes there was >90% probability that the treatment is superior to control. For 8/113, this probability was >95%.

eTable 6: Results from analyses of negative studies using ratio as effect measure (odds ratio, risk ratio, incidence rate ratio, hazard ratio, geometric mean ratio), with prior for the log ratio $\mu_{i}\sim N(0, \sigma^{2}=1/5)$. ES: effect size. P(ES<0.80) denotes the posterior probability that the effect size is <0.80. CrI: credible interval. HR: Hazard ratio. GMR: geometric mean ratio. OR: risk ratio. RR: risk ratio. IRR: incidence rate ratio.

| **Study** | **Measure** | **Estimated** | **P(ES<0.80)** | **P(ES<0.90)** | **P(ES<0.95)** | **P(ES<1.00)** |
| --- | --- | --- | --- | --- | --- | --- |
| JAMA. 2021;325(11):1061-1073 | HR | 1.09 [0.96;1.23] | 0% | 0.1% | 1.8% | 9.8% |
| JAMA. 2021;325(11):1061-1073 | HR | 1.09 [0.95;1.24] | 0% | 0.2% | 2.1% | 10.2% |
| JAMA. 2021;325(11):1061-1073 | HR | 0.92 [0.81;1.04] | 1.5% | 37.9% | 70.1% | 90.6% |
| JAMA. 2021;325(11):1061-1073 | HR | 0.92 [0.81;1.05] | 2% | 37.6% | 69.2% | 89.8% |
| JAMA. 2021;325(9):833-842 | HR | 0.74 [0.52;1.07] | 65.3% | 85% | 90.9% | 94.7% |
| JAMA. 2021;325(15):1513-1523 | GMR | 1.19 [0.98;1.45] | 0% | 0.3% | 1.3% | 4.4% |
| JAMA. 2021;326(24):2478-2487 | RR | 0.87 [0.74;1.03] | 14.5% | 63.9% | 84.3% | 94.9% |
| JAMA. 2021;326(4):314-323 | HR | 0.92 [0.81;1.05] | 1.5% | 35.8% | 68% | 89.4% |
| JAMA. 2021;326(4):314-323 | OR | 0.94 [0.83;1.08] | 0.6% | 23.6% | 53.6% | 80.6% |
| JAMA. 2021;326(11):1024-1033 | HR | 1.03 [0.87;1.22] | 0.2% | 5.7% | 17.4% | 36.8% |
| JAMA. 2021;326(12):1158-1169 | OR | 0.79 [0.55;1.15] | 52.1% | 74.9% | 83.2% | 89.1% |
| JAMA. 2021;326(15):1494-1503 | RR | 1.07 [0.86;1.31] | 0.4% | 5.9% | 14.3% | 27.9% |
| JAMA. 2021;325(3):254-264 | ratio | 1.05 [0.99;1.11] | 0% | 0% | 0% | 4.1% |
| JAMA. 2021;325(3):254-264 | ratio | 0.95 [0.9;1.01] | 0% | 3.2% | 49.7% | 95.9% |
| JAMA. 2021;325(14):1426-1435 | HR | 0.94 [0.76;1.15] | 6.4% | 34.3% | 54.9% | 73.4% |
| JAMA. 2021;325(18):1841-1851 | HR | 1.03 [0.73;1.47] | 7.9% | 22.3% | 32.2% | 43.1% |
| JAMA. 2021;326(11):1013-1023 | RR | 1.05 [0.84;1.31] | 1.1% | 9.6% | 20.1% | 34.6% |
| JAMA. 2021;325(13):1277-1285 | HR | 0.89 [0.77;1.04] | 7.4% | 53.9% | 79% | 93% |
| JAMA. 2021;325(19):1946-1954 | HR | 1.1 [0.88;1.38] | 0.3% | 3.9% | 10.1% | 20.2% |
| JAMA. 2021;325(20):2056-2066 | OR | 0.98 [0.7;1.38] | 11.5% | 30.5% | 42.4% | 54.3% |
| JAMA. 2021;326(22):2268-2276 | RR | 1.32 [0.94;1.86] | 0.2% | 1.4% | 3% | 5.6% |
| JAMA. 2021;325(1):39-49 | HR | 1.06 [0.97;1.16] | 0% | 0% | 0.9% | 10.3% |
| JAMA. 2021;326(9):818-829 | HR | 0.97 [0.9;1.05] | 0% | 2.8% | 30.4% | 78.6% |
| JAMA. 2021;326(9):830-838 | HR | 1.03 [0.96;1.11] | 0% | 0% | 1.5% | 21% |
| N Engl J Med 2021; 384:905-914 | HR | 0.95 [0.7;1.29] | 13.5% | 36.6% | 50.3% | 63.5% |
| N Engl J Med 2021;384:808-17 | OR | 0.98 [0.73;1.32] | 8.7% | 28.4% | 41.5% | 54.7% |
| N Engl J Med 2021; 384:1003-1014 | HR | 0.78 [0.53;1.13] | 55.9% | 77.8% | 85.2% | 90.6% |
| N Engl J Med 2021; 384:1003-1014 | HR | 0.93 [0.62;1.4] | 23.4% | 43.6% | 53.8% | 63.5% |
| N Engl J Med 2021;384:2283-94 | RR | 1.04 [0.94;1.14] | 0% | 0.1% | 3.3% | 21% |
| N Engl J Med 2021;385:119-29 | RR | 0.8 [0.63;1.02] | 49.8% | 82.8% | 91.9% | 96.6% |
| N Engl J Med 2021;385:119-29 | RR | 0.83 [0.63;1.09] | 39.9% | 72.3% | 83.8% | 91.1% |
| N Engl J Med 2021;385:2544-53 | HR | 1.26 [0.99;1.59] | 0% | 0.3% | 1% | 2.9% |
| N Engl J Med 2021;385:395-405 | HR | 0.99 [0.85;1.15] | 0.3% | 10.4% | 29.3% | 55.2% |
| N Engl J Med 2021;384:1089-100 | HR | 1.02 [0.81;1.28] | 1.9% | 14.4% | 27.6% | 43.6% |
| N Engl J Med 2021; 384:895-904 | RR | 0.95 [0.87;1.04] | 0% | 12.6% | 49.2% | 85.7% |
| N Engl J Med 2021; 384:895-904 | RR | 0.98 [0.94;1.03] | 0% | 0% | 9.4% | 80.6% |
| N Engl J Med 2021; 384:895-904 | RR | 1.04 [0.88;1.22] | 0.1% | 4.4% | 14.4% | 32.3% |
| N Engl J Med 2021; 384:895-904 | RR | 1 [0.93;1.08] | 0% | 0.3% | 9.1% | 50% |
| N Engl J Med 2021;384:1789-99 | HR | 0.97 [0.84;1.12] | 0.4% | 14.7% | 38.4% | 66.4% |
| N Engl J Med 2021;385:436-44 | RR | 1.14 [0.8;1.62] | 2.6% | 9.4% | 15.6% | 23.4% |
| N Engl J Med 2021; 384:1981-1990 | HR | 1.02 [0.91;1.14] | 0% | 1.4% | 10.7% | 36.4% |
| N Engl J Med 2021;384:1910-20 | RR | 0.86 [0.68;1.09] | 27.7% | 65.4% | 80.1% | 89.8% |
| N Engl J Med 2021;385:1833-44 | OR | 0.86 [0.64;1.15] | 32.7% | 63.1% | 75.9% | 85.4% |
| N Engl J Med 2021; 384:1885-1898 | RR | 0.85 [0.51;1.44] | 40.4% | 58% | 65.5% | 72.1% |
| N Engl J Med 2021;385:516-25 | RR | 0.91 [0.7;1.18] | 17.5% | 47.5% | 63.2% | 76.2% |
| N Engl J Med 2021;384:417-27 | RR | 0.89 [0.58;1.37] | 31% | 51.5% | 61.2% | 69.7% |
| N Engl J Med 2021;385:2025-35 | RR | 1.03 [0.84;1.26] | 0.8% | 9.8% | 22.2% | 39.3% |
| N Engl J Med 2021;385:1845-55 | HR | 0.9 [0.78;1.04] | 4.7% | 48.6% | 76.4% | 92.3% |
| N Engl J Med 2021;385:297-308 | HR | 1.23 [0.78;1.92] | 3.1% | 8.6% | 13.2% | 18.8% |
| N Engl J Med 2021;384:1301-11 | RR | 1.02 [0.94;1.11] | 0% | 0.2% | 4.5% | 32.1% |
| N Engl J Med 2021;384:619-29 | OR | 1.15 [0.76;1.75] | 4.4% | 12.4% | 18.3% | 25.1% |
| N Engl J Med 2021;384:2028-38 | RR | 0.79 [0.6;1.04] | 53.2% | 82% | 90.2% | 95.2% |
| N Engl J Med 2021; 384:216-228 | HR | 0.8 [0.64;1] | 48.8% | 84.2% | 93% | 97.4% |
| N Engl J Med 2021; 384:216-228 | HR | 0.87 [0.69;1.1] | 24.7% | 61% | 76.7% | 87.6% |
| Lancet 2021; 398: 991–1001 | HR | 0.88 [0.75;1.05] | 12.5% | 57.5% | 79% | 92.1% |
| Lancet 2021; 398: 1417–26 | HR | 0.89 [0.72;1.11] | 16.3% | 53.1% | 71.3% | 84.4% |
| Lancet 2021; 397: 2182–93 | HR | 0.94 [0.82;1.08] | 1% | 25.8% | 55.2% | 80.8% |
| Lancet 2021; 397: 2182–93 | HR | 0.96 [0.84;1.1] | 0.4% | 18.1% | 44% | 71.5% |
| Lancet 2021; 398: 1687–99 | RR | 0.91 [0.81;1.02] | 1.3% | 41.2% | 76.6% | 94.6% |
| Lancet 2021; 398: 1687–99 | RR | 0.9 [0.77;1.06] | 6.7% | 48.7% | 74% | 90% |
| Lancet 2021; 398: 1687–99 | RR | 0.97 [0.83;1.14] | 1% | 17.9% | 39.6% | 63.6% |
| Lancet 2021; 398: 1687–99 | RR | 0.98 [0.87;1.1] | 0% | 7.4% | 29.4% | 63% |
| Lancet 2021; 397: 112–18 | OR | 1.15 [0.89;1.48] | 0.3% | 3% | 7.1% | 14.3% |
| Lancet 2021; 397: 605–12 | HR | 0.97 [0.88;1.08] | 0% | 7.5% | 34.2% | 71.7% |
| Lancet 2021; 397: 2049–59 | HR | 1 [0.93;1.07] | 0% | 0.1% | 7.6% | 50.5% |
| Lancet 2021; 398: 1507–16 | HR | 0.82 [0.63;1.06] | 44.3% | 77.3% | 87.5% | 93.7% |
| BMJ 2021;373:n1162 | OR | 0.86 [0.66;1.14] | 29.6% | 61.5% | 74.7% | 84.8% |
| BMJ 2021;374:n1585 | HR | 0.95 [0.78;1.17] | 4.5% | 29.2% | 48.8% | 67.9% |
| BMJ 2021;372:n716 | RR | 1.16 [0.9;1.49] | 0.2% | 2.7% | 6.5% | 13.1% |
| BMJ 2021;374:n2103 | GMR | 0.75 [0.55;1.03] | 65.4% | 87.1% | 92.9% | 96.4% |
| BMJ 2021;372:n740 | IRR | 0.83 [0.67;1.02] | 36.8% | 77.5% | 89.6% | 95.9% |
| BMJ 2021;373:n893 | RR | 0.78 [0.59;1.02] | 57.5% | 85.2% | 92.5% | 96.5% |
| BMJ 2021;374:n2106 | HR | 0.92 [0.65;1.32] | 21.8% | 44.6% | 56.3% | 67.3% |
| BMJ 2021;374:n2106 | HR | 0.92 [0.65;1.32] | 21.2% | 44.2% | 56.3% | 67% |
| BMJ 2021;374:n1857 | RR | 1.03 [0.88;1.2] | 0.1% | 4.1% | 15.1% | 35.8% |
| BMJ 2021;374:n1857 | RR | 1.01 [0.77;1.33] | 4.9% | 20.7% | 33.5% | 47.4% |
| BMJ 2021;372:n256 | HR | 0.86 [0.72;1.01] | 22.2% | 72% | 88.3% | 96.3% |
| BMJ 2021;375:n2400 | OR | 0.75 [0.5;1.14] | 62.2% | 80.7% | 86.8% | 91.3% |
| BMJ 2021;372:n355 | OR | 0.97 [0.87;1.09] | 0% | 9.5% | 35.5% | 69.8% |
| BMJ 2021;372:n84 | OR | 1.25 [0.68;2.3] | 7.8% | 14.8% | 19.1% | 23.9% |
| BMJ 2021;372:m4786 | RR | 1.02 [0.93;1.12] | 0% | 0.6% | 7.4% | 34.2% |
| Ann Intern Med. 2021;174:145–156 | HR | 0.94 [0.77;1.15] | 5.3% | 32.3% | 52.8% | 71.5% |
| Ann Intern Med. 2021;174:344–352 | HR | 1.08 [0.71;1.65] | 8.5% | 20.6% | 28.4% | 36.9% |
| Ann Intern Med. 2021;174:335–343 | OR | 0.86 [0.57;1.28] | 37.1% | 59.8% | 69.5% | 77.6% |
| Ann Intern Med. 2021;174:1367-1376 | HR | 0.97 [0.65;1.44] | 17.6% | 36.3% | 46.3% | 56.1% |
| Ann Intern Med. 2021;174:889–898 | RR | 0.95 [0.87;1.05] | 0% | 11.7% | 47.1% | 83.7% |
| Ann Intern Med. 2021;174:1683-1692 | OR | 0.91 [0.69;1.2] | 17.9% | 46.8% | 61.8% | 74.7% |
| PLoS Med 18(9): e1003744 | RR | 1.08 [0.99;1.16] | 0% | 0% | 0.1% | 3.8% |
| PLoS Med 18(9): e1003744 | RR | 1.11 [0.96;1.29] | 0% | 0.3% | 2.1% | 8.9% |
| PLoS Med 18(5): e1003628 | OR | 0.97 [0.66;1.42] | 16.1% | 35% | 45.7% | 56.1% |
| PLoS Med 18(6): e1003670 | OR | 1.07 [0.72;1.59] | 7.9% | 20.2% | 28.3% | 37.3% |
| PLoS Med 18(5): e1003630 | OR | 1.11 [0.69;1.75] | 8.7% | 19.7% | 26.4% | 33.9% |
| PLoS Med 18(1): e1003475 | RR | 0.96 [0.9;1.03] | 0% | 2.5% | 35.8% | 87.6% |
| PLoS Med 18(1): e1003475 | RR | 0.95 [0.88;1.04] | 0% | 9.4% | 47.1% | 86.5% |
| PLoS Med 18(10): e1003808 | RR | 0.91 [0.81;1.03] | 1.6% | 42.2% | 74.7% | 93.2% |
| PLoS Med 18(1): e1003411 | RR | 1.09 [0.86;1.39] | 0.6% | 6.1% | 13% | 23.9% |
| PLoS Med 18(5): e1003650 | RR | 1.31 [0.76;2.28] | 3.9% | 8.8% | 12.3% | 16.5% |
| PLoS Med 18(5): e1003650 | HR | 0.9 [0.61;1.33] | 28.8% | 50.9% | 61.7% | 71% |
| PLoS Med 18(5): e1003650 | HR | 1.1 [0.79;1.52] | 3.2% | 12.2% | 20% | 29.2% |
| PLoS Med 18(5): e1003650 | HR | 1.07 [0.74;1.55] | 5.8% | 17.7% | 25.9% | 35.3% |
| PLoS Med 18(5): e1003650 | HR | 1.06 [0.68;1.65] | 10.9% | 23.7% | 31.7% | 40.2% |
| PLoS Med 18(5): e1003650 | RR | 0.94 [0.52;1.7] | 29.9% | 44.3% | 51.3% | 58.2% |
| PLoS Med 18(4): e1003609 | RRR | 0.96 [0.77;1.19] | 5% | 28% | 46.1% | 64.2% |
| PLoS Med 18(9): e1003680 | OR | 0.96 [0.84;1.1] | 0.5% | 17.9% | 43.8% | 71.6% |
| PLoS Med 18(9): e1003680 | OR | 1.02 [0.87;1.2] | 0.2% | 6.8% | 20% | 40.7% |
| PLoS Med 18(3): e1003506 | OR | 0.89 [0.59;1.32] | 30.4% | 52.7% | 63% | 71.9% |
| PLoS Med 18(3): e1003506 | OR | 0.9 [0.6;1.34] | 29.3% | 50.9% | 61.1% | 70.1% |
| PLoS Med 18(5): e1003601 | RR | 0.94 [0.64;1.38] | 21.3% | 42.1% | 53.1% | 63.3% |
| PLoS Med 18(6): e1003668 | HR | 0.9 [0.58;1.4] | 29.4% | 49.5% | 59.1% | 67.7% |
| PLoS Med 18(10): e1003798 | OR | 0.77 [0.46;1.3] | 54.8% | 71.6% | 78.2% | 83.7% |
| PLoS Med 18(10): e1003798 | OR | 0.81 [0.48;1.36] | 47.7% | 64.8% | 72% | 78.2% |
| PLoS Med 18(7): e1003689 | OR | 0.98 [0.68;1.39] | 13.4% | 32.4% | 43.9% | 55.5% |
| PLoS Med 18(7): e1003694 | IRR | 0.9 [0.75;1.09] | 9.4% | 47.7% | 69.9% | 85.8% |

**Survival outcomes**

We focus on estimates on survival outcomes where a ratio was used as the measure of effect. We included 18 estimates from 15 studies. In essence, from the 113 results on ratios shown above, we only keep here ones where the outcome was survival. **eTable 7** shows the results, also summarized in

**eFigure 6** below.

eTable 7: Results from analyses on survival outcome using ratio as effect measure (odds ratio, risk ratio, or hazard ratio). ES: effect measure. P(ES<0.80) denotes the posterior probability that the effect size is <0.80. Lower values favor the experimental treatment.

| **Reference** | **Measure** | **Estimated Effect size [95% CrI]** | $\boldsymbol{P(ES<0.80)}$ | $\boldsymbol{P(ES<0.90)}$ | $\boldsymbol{P(ES<0.95)}$ | $\boldsymbol{P(ES<1.00)}$ |
| --- | --- | --- | --- | --- | --- | --- |
| JAMA. 2021;325(9):833-842 | HR | 0.7 [0.47;1.03] | 74.8% | 89.6% | 93.8% | 96.3% |
| JAMA. 2021;325(13):1277-1285 | HR | 0.89 [0.76;1.03] | 8.4% | 55.7% | 79.9% | 93.2% |
| JAMA. 2021;326(9):818-829 | HR | 0.97 [0.9;1.05] | 0% | 2.9% | 29.9% | 78.3% |
| JAMA. 2021;326(9):830-838 | HR | 1.03 [0.96;1.11] | 0% | 0% | 1.4% | 21.5% |
| N Engl J Med 2021;384:2283-94 | RR | 1.04 [0.94;1.14] | 0% | 0.1% | 3.2% | 21.3% |
| N Engl J Med 2021;385:119-29 | RR | 0.79 [0.61;1.01] | 55% | 85.1% | 92.8% | 96.8% |
| N Engl J Med 2021;385:119-29 | RR | 0.81 [0.61;1.08] | 45.7% | 75.9% | 85.8% | 92.2% |
| N Engl J Med 2021;385:2544-53 | HR | 1.28 [1;1.63] | 0% | 0.2% | 0.9% | 2.5% |
| N Engl J Med 2021;385:395-405 | HR | 0.99 [0.85;1.15] | 0.3% | 10.7% | 29.8% | 55.5% |
| N Engl J Med 2021;384:1301-11 | RR | 1.02 [0.94;1.11] | 0% | 0.1% | 4.8% | 32.7% |
| N Engl J Med 2021;384:2028-38 | RR | 0.77 [0.58;1.04] | 60.5% | 85.5% | 92.3% | 96.1% |
| Lancet 2021; 397: 2182–93 | HR | 0.94 [0.82;1.08] | 1.1% | 27.1% | 55.8% | 81.3% |
| Lancet 2021; 397: 2182–93 | HR | 0.96 [0.83;1.1] | 0.5% | 18.1% | 44.1% | 71.2% |
| Lancet 2021; 397: 605–12 | HR | 0.97 [0.87;1.07] | 0% | 7.9% | 34.9% | 71.5% |
| Lancet 2021; 397: 2049–59 | HR | 1 [0.93;1.07] | 0% | 0.1% | 7.7% | 50% |
| BMJ 2021;372:n355 | OR | 0.97 [0.86;1.08] | 0% | 9.7% | 35.9% | 69.9% |
| PLoS Med 18(5): e1003650 | RR | 1.57 [0.78;3.15] | 3% | 6.1% | 8% | 10.3% |
| PLoS Med 18(5): e1003650 | RR | 0.89 [0.4;1.98] | 39.9% | 51.1% | 56.4% | 60.9% |

eFigure 6: Posterior probability that the true treatment effect (odds, risk, or hazard ratio) is <1. This is the probability that the experimental treatment is better than control, for survival outcomes.

We can see that for many of these negative trials there is a very high posterior probability that the treatment offers survival benefit. In 5/18 (28%) instances, the estimated probability that the experimental treatment offers a benefit in survival was ≥90%; in 3/18 (17%) of the estimates the probability that treatment increases survival was ≥96%.

**Composite outcomes including survival**

We now focus composite outcomes that in their definition included survival. A total of 25 trials provided information on 28 effects. Results are shown in **eTable 8** and summarized in **eFigure 7**. In 5/28 of the estimates (18%) there was a ≥90% probability that the experimental treatment was better than the control. In 2/28 estimates (7%) this probability was 95% or more.

eTable 8: Results from the analysis of composite outcomes that included survival. ES: effect measure. P(ES<0.80) denotes the posterior probability that the effect size is <0.80, where lower values favor the experimental treatment.

| **Reference** | **Measure** | **Estimated Effect size [95% CrI]** | $\boldsymbol{P(ES<0.80)}$ | $\boldsymbol{P(ES<0.90)}$ | $\boldsymbol{P(ES<0.95)}$ | $\boldsymbol{P(ES<1.00)}$ |
| --- | --- | --- | --- | --- | --- | --- |
| JAMA. 2021;326(24):2478-2487 | RR | 0.87 [0.74;1.03] | 15.4% | 65.3% | 84.7% | 94.8% |
| JAMA. 2021;326(4):314-323 | HR | 0.92 [0.81;1.05] | 1.7% | 37.2% | 68.3% | 89.6% |
| JAMA. 2021;326(15):1494-1503 | RR | 1.07 [0.86;1.33] | 0.4% | 5.7% | 14% | 26.9% |
| JAMA. 2021;325(18):1841-1851 | HR | 1.04 [0.71;1.53] | 9.1% | 23.4% | 32.4% | 42.4% |
| JAMA. 2021;325(19):1946-1954 | HR | 1.11 [0.88;1.41] | 0.3% | 4% | 9.8% | 19.4% |
| JAMA. 2021;325(1):39-49 | HR | 1.06 [0.98;1.17] | 0% | 0% | 0.9% | 10.4% |
| N Engl J Med 2021;384:808-17 | OR | 0.98 [0.71;1.33] | 10.3% | 29.9% | 42.5% | 55.5% |
| N Engl J Med 2021; 384:895-904 | RR | 1.04 [0.88;1.23] | 0.1% | 4.6% | 14.6% | 32.3% |
| N Engl J Med 2021;385:436-44 | RR | 1.17 [0.8;1.72] | 2.5% | 9% | 14.6% | 21.5% |
| N Engl J Med 2021; 384:1981-1990 | HR | 1.02 [0.91;1.14] | 0% | 1.5% | 11% | 36.2% |
| N Engl J Med 2021;385:516-25 | RR | 0.9 [0.69;1.19] | 19.9% | 50.3% | 65.4% | 77.7% |
| N Engl J Med 2021;385:2025-35 | RR | 1.03 [0.84;1.27] | 0.8% | 9.8% | 22% | 38.9% |
| N Engl J Med 2021;385:1845-55 | HR | 0.9 [0.78;1.04] | 5.4% | 50.2% | 77% | 92.3% |
| N Engl J Med 2021;385:297-308 | HR | 1.32 [0.78;2.23] | 3.2% | 7.9% | 11.2% | 15.3% |
| N Engl J Med 2021; 384:216-228 | HR | 0.79 [0.63;1] | 54.5% | 86.6% | 94.1% | 97.8% |
| N Engl J Med 2021; 384:216-228 | HR | 0.86 [0.67;1.1] | 27.8% | 63.6% | 78% | 88% |
| Lancet 2021; 398: 991–1001 | HR | 0.88 [0.74;1.05] | 14.2% | 59.9% | 80.4% | 92.3% |
| BMJ 2021;373:n1162 | OR | 0.85 [0.6;1.08] | 34% | 64.8% | 77.3% | 86.1% |
| BMJ 2021;374:n2106 | HR | 0.91 [0.62;1.35] | 25.9% | 47.4% | 58.3% | 67.9% |
| BMJ 2021;374:n2106 | HR | 0.91 [0.62;1.35] | 25.7% | 47.9% | 58.7% | 68.3% |
| BMJ 2021;374:n1857 | RR | 1.03 [0.89;1.21] | 0.1% | 4.5% | 15.1% | 35.4% |
| BMJ 2021;374:n1857 | RR | 1.01 [0.76;1.36] | 5.8% | 21.9% | 33.8% | 47.2% |
| BMJ 2021;375:n2400 | OR | 0.69 [0.43;1.1] | 73.5% | 86.9% | 91.1% | 94.1% |
| BMJ 2021;372:n84 | OR | 1.54 [0.66;3.66] | 6.7% | 11% | 13.4% | 16.2% |
| BMJ 2021;372:m4786 | RR | 1.02 [0.93;1.13] | 0% | 0.6% | 7.7% | 34.6% |
| Ann Intern Med. 2021;174:145–156 | HR | 0.94 [0.76;1.15] | 6.3% | 34.2% | 54.2% | 72.2% |
| PLoS Med 18(3): e1003506 | OR | 0.86 [0.55;1.36] | 38.1% | 58% | 66.8% | 74.5% |
| PLoS Med 18(7): e1003689 | OR | 0.97 [0.66;1.42] | 16.2% | 35.1% | 45.7% | 56% |

eFigure 7: Posterior probability that the true treatment effect (odds, risk, or hazard ratio) is <1. This is the probability that the experimental treatment is better than control, for composite outcomes including survival.

## 6.2 Absolute outcomes (differences)

Results from the negative trials providing results on differences are shown below.

eTable 9: Results from analyses of negative trials, absolute effect measures. ES: effect measure, can be risk or mean difference. P(ES<0) denotes the posterior probability that the effect size is <0, favoring the experimental treatment

| **Study** | **Estimated** | **P(ES<0)** |
| --- | --- | --- |
| JAMA. 2021;326(15):1485-1493 | -0.4 [-5.2;4.3] | 56% |
| JAMA. 2021;326(15):1485-1493 | 0.4 [-4.3;5.2] | 43% |
| JAMA. 2021;325(4):363-372 | -1.4 [-3;0.3] | 95% |
| JAMA. 2021;326(20):2021-2030 | 0.2 [-1.5;1.9] | 41% |
| JAMA. 2021;326(20):2021-2030 | -0.4 [-0.9;0.2] | 93% |
| JAMA. 2021;326(3):230-239 | -3.1 [-9.3;3.1] | 83% |
| JAMA. 2021;326(24):2488-2497 | 2.6 [-2.2;7.3] | 14% |
| JAMA. 2021;325(10):942-951 | 0.3 [-2.6;3.1] | 41% |
| JAMA. 2021;325(7):632-644 | 0.31 [-0.13;0.76] | 8% |
| JAMA. 2021;325(7):632-644 | -0.27 [-0.71;0.16] | 89% |
| JAMA. 2021;325(7):632-644 | 0.09 [-0.35;0.52] | 34% |
| JAMA. 2021;325(17):1731-1743 | -2 [-6;2] | 84% |
| JAMA. 2021;325(16):1620-1630 | 1.5 [-6.6;9.8] | 36% |
| JAMA. 2021;326(13):1277-1285 | 0.5 [-6.2;7.3] | 44% |
| JAMA. 2021;326(2):137-144. | 2.7 [-3.3;8.8] | 19% |
| JAMA. 2021;325(2):138-145 | 2.9 [NA;8] | 18% |
| JAMA. 2021;325(2):138-145 | 1.3 [NA;6.5] | 34% |
| JAMA. 2021;325(13):1266-1276 | -8.7 [-34.4;17] | 73% |
| JAMA. 2021;325(7):646-657 | 0.7 [-0.1;1.6] | 5% |
| JAMA. 2021;325(7):646-657 | 58 [-165;282] | 45% |
| JAMA. 2021;325(7):646-657 | 0.2 [-0.6;1.1] | 32% |
| JAMA. 2021;325(7):646-657 | 21 [-193;235] | 48% |
| JAMA. 2021;325(6):542-551 | 0.4 [-0.6;1.4] | 22% |
| JAMA. 2021;326(23):2385-2394 | 0.03 [-0.01;0.07] | 7% |
| JAMA. 2021;326(6):490-498 | 0 [-15;14] | 50% |
| JAMA. 2021;326(16):1595-1605 | 1 [-3;6] | 33% |
| JAMA. 2021;326(19):1919-1929 | 2.5 [-3.5;8.5] | 21% |
| JAMA. 2021;325(8):742-751 | 1 [-2;4] | 26% |
| JAMA. 2021;326(10):926-939 | 1.26 [-0.59;3.11] | 9% |
| JAMA. 2021;325(17):1744-1754 | 1.5 [-8.2;11.1] | 38% |
| N Engl J Med 2021;385:1372-81 | -1 [-12;10] | 57% |
| N Engl J Med 2021;385:982-95 | 0.3 [-0.48;1.08] | 23% |
| N Engl J Med 2021;385:982-95 | -0.23 [-1;0.55] | 72% |
| Lancet 2021; 398: 1487–97 | -1.74 [-7.17;3.69] | 74% |
| Lancet 2021; 397: 1554–63 | -3.4 [-9.2;2.4] | 87% |
| Lancet 2021; 397: 2372–84 | -5.2 [-10.5;0] | 97% |
| Lancet 2021; 397: 2372–84 | -3.8 [-12;4.3] | 82% |
| Lancet 2021; 398: 416–28 | -1.11 [-4.47;2.26] | 74% |
| Lancet 2021; 398: 416–28 | -0.66 [-4.52;3.2] | 63% |
| Lancet 2021; 397: 305–17 | -11 [-33;11] | 82% |
| BMJ 2021;375:e068060 | -5.5 [-18.8;7.8] | 79% |
| BMJ 2021;372:n135 | -0.11 [-0.36;0.14] | 80% |
| BMJ 2021;374:n1506 | -1.8 [-5.6;2] | 82% |
| BMJ 2021;373:n1014 | -1.06 [-3.43;1.32] | 81% |
| BMJ 2021;373:n1205 | -0.3 [-1.3;0.8] | 71% |
| BMJ 2021;374:n2209 | -0.2 [-0.9;0.5] | 72% |
| BMJ 2021;373:n1022 | 1 [0;1.9] | 2% |
| BMJ 2021;372:m4903 | 1.9 [-0.3;4.2] | 5% |
| Ann Intern Med. 2021;174:1282-1292 | -0.09 [-2.76;2.58] | 53% |
| Ann Intern Med. 2021;174:462–471 | 2.3 [-0.9;5.5] | 8% |
| PLoS Med 18(10): e1003839 | 0.07 [-0.06;0.2] | 15% |
| PLoS Med 18(9): e1003617 | 0.03 [-0.09;0.15] | 33% |
| PLoS Med 18(9): e1003617 | -0.01 [-0.11;0.13] | 55% |
| PLoS Med 18(2): e1003448 | 0.5 [-3.1;2.1] | 35% |
| PLoS Med 18(11): e1003847 | 0.9 [-0.27;2.2] | 8% |
| PLoS Med 18(1): e1003433 | -0.35 [-1.51;0.81] | 72% |

# Section 7. Detailed results from analyses on positive studies

Below we show the complete results of the analysis of positive studies. We present results from the main analysis. The sensitivity analysis with different choices for the prior distribution led to largely similar results.

eTable 10: Analysis results from 234 estimates of HR in oncology trials.

| **Study** | **Estimated Effect size**  **[95% CrI]** | $\boldsymbol{P(HR<0.80)}$ | $\boldsymbol{P(HR<0.90)}$ | $\boldsymbol{P(HR<0.95)}$ | $\boldsymbol{P(HR<1.00)}$ |
| --- | --- | --- | --- | --- | --- |
| JAMA. 2010;304(19):2154-2160 | 0.49 [0.3;0.8] | 97% | 99% | 100% | 100% |
| N Engl J Med 2018; 379:54-63 | 0.76 [0.63;0.92] | 70% | 96% | 99% | 100% |
| Lancet 2019; 393: 1948–57 | 0.77 [0.64;0.94] | 65% | 94% | 98% | 100% |
| J Clin Oncol 27:5062-5067 | 0.84 [0.72;0.98] | 27% | 81% | 94% | 99% |
| J Clin Oncol 34:972-979 | 0.69 [0.53;0.9] | 86% | 97% | 99% | 100% |
| J Clin Oncol 27:3109-3116 | 0.84 [0.71;1] | 29% | 79% | 92% | 98% |
| N Engl J Med 2018; 379:2342-235 | 0.68 [0.47;1] | 80% | 93% | 96% | 98% |
| Lancet Oncol 2016; 17: 1248–60 | 0.7 [0.55;0.9] | 85% | 98% | 99% | 100% |
| Lancet Oncol 2017; 18: 611–22 | 0.84 [0.7;0.99] | 29% | 78% | 92% | 97% |
| Lancet Oncol 2012; 13: 671–78 | 0.68 [0.47;0.98] | 81% | 93% | 96% | 98% |
| J Clin Oncol 30:2718-2724 | 0.45 [0.24;0.81] | 97% | 99% | 99% | 100% |
| Lancet Oncol 2019; 20: 494–503 | 0.53 [0.3;0.94] | 92% | 97% | 98% | 99% |
| Lancet 2010; 376: 687–97 | 0.74 [0.6;0.91] | 77% | 97% | 99% | 100% |
| Lancet 2012; 379: 315–21 | 0.72 [0.52;1] | 74% | 91% | 95% | 98% |
| J Clin Oncol 33:3858-3865 | 0.56 [0.35;0.87] | 94% | 98% | 99% | 99% |
| N Engl J Med 2012; 366:109-119 | 0.64 [0.47;0.88] | 92% | 98% | 99% | 100% |
| Lancet Oncol 2015; 16: 1037–48 | 0.65 [0.49;0.88] | 92% | 98% | 99% | 100% |
| N Engl J Med 2014; 371:424-433 | 0.71 [0.6;0.84] | 92% | 100% | 100% | 100% |
| J Clin Oncol 27:4454-4461 | 0.78 [0.61;0.99] | 58% | 88% | 95% | 98% |
| N Engl J Med 2017; 376:1015-1026 | 0.73 [0.59;0.91] | 79% | 97% | 99% | 100% |
| N Engl J Med 2014; 371:906-917 | 0.78 [0.64;0.96] | 59% | 92% | 97% | 99% |
| Lancet Oncol 2013; 14: 29–37 | 0.81 [0.69;0.94] | 44% | 91% | 98% | 100% |
| J Clin Oncol 30:2585-2592 | 0.74 [0.57;0.96] | 72% | 93% | 97% | 99% |
| Lancet Oncol 2010; 11: 1066–73 | 0.6 [0.45;0.8] | 97% | 100% | 100% | 100% |
| Lancet Oncol 2010; 11: 21–28 | 0.73 [0.56;0.95] | 75% | 94% | 97% | 99% |
| N Engl J Med 2015;373:1627-39 | 0.73 [0.6;0.89] | 82% | 98% | 100% | 100% |
| N Engl J Med 2015;373:123-35 | 0.59 [0.44;0.79] | 98% | 100% | 100% | 100% |
| Lancet 2017; 389: 56–66 | 0.63 [0.5;0.79] | 98% | 100% | 100% | 100% |
| N Engl J Med 2016;374:1344-55 | 0.59 [0.42;0.83] | 96% | 99% | 100% | 100% |
| N Engl J Med 2015; 373:2425-2437 | 0.16 [0.05;0.56] | 100% | 100% | 100% | 100% |
| J Clin Oncol 28:29-34 | 0.78 [0.61;0.99] | 58% | 88% | 95% | 98% |
| N Engl J Med 2014; 371:213-223 | 0.43 [0.24;0.79] | 98% | 99% | 100% | 100% |
| Lancet Oncol 2010; 11: 521–29 | 0.81 [0.7;0.95] | 43% | 91% | 98% | 100% |
| Lancet 2012; 379: 1508–16 | 0.69 [0.49;0.98] | 80% | 93% | 97% | 98% |
| Lancet Oncol 2018; 19: 1247–58 | 0.65 [0.45;0.93] | 87% | 96% | 98% | 99% |
| Lancet Oncol 2009; 10: 25–34 | 0.68 [0.5;0.93] | 85% | 96% | 98% | 99% |
| J Clin Oncol 36:2259-2266 | 0.67 [0.47;0.96] | 84% | 95% | 97% | 99% |
| N Engl J Med 2015;373:1814-23 | 0.67 [0.51;0.89] | 89% | 98% | 99% | 100% |
| Lancet 2009; 374: 1432–40 | 0.79 [0.65;0.95] | 55% | 91% | 97% | 99% |
| Lancet 2019; 393: 156–67 | 0.8 [0.65;0.98] | 50% | 87% | 95% | 98% |
| N Engl J Med 2011; 364:1817-1825 | 0.57 [0.45;0.73] | 100% | 100% | 100% | 100% |
| N Engl J Med 2018; 379:2395-2406 | 0.64 [0.48;0.86] | 93% | 99% | 100% | 100% |
| J Clin Oncol 29:3247-3254 | 0.66 [0.46;0.94] | 86% | 95% | 98% | 99% |
| Lancet 2011; 377: 914–23 | 0.81 [0.66;0.99] | 45% | 85% | 94% | 98% |
| Lancet Oncol 2015; 16: 1306–15 | 0.81 [0.65;0.98] | 46% | 85% | 94% | 98% |
| (online) | 0.67 [0.52;0.86] | 92% | 99% | 100% | 100% |
| Lancet 2010; 376: 1147–54 | 0.7 [0.59;0.83] | 94% | 100% | 100% | 100% |
| N Engl J Med 2011; 364:1995-2005 | 0.65 [0.54;0.77] | 99% | 100% | 100% | 100% |
| Lancet 2015; 385: 1863–72 | 0.65 [0.51;0.84] | 95% | 99% | 100% | 100% |
| Lancet Oncol 2017; 18: 1327–37 | 0.79 [0.65;0.96] | 55% | 90% | 97% | 99% |
| N Engl J Med 2013; 369:1023-1034 | 0.78 [0.62;0.99] | 59% | 89% | 95% | 98% |
| Lancet Oncol 2016; 17: 727–37 | 0.55 [0.3;1] | 89% | 94% | 96% | 97% |
| J Clin Oncol 29:1678-1685 | 0.68 [0.49;0.95] | 83% | 95% | 98% | 99% |
| Lancet Oncol 2018; 19: 1315–27 | 0.76 [0.58;0.98] | 65% | 90% | 95% | 98% |
| Lancet Oncol 2018; 19: 1315–27 | 0.61 [0.47;0.79] | 98% | 100% | 100% | 100% |
| Lancet 2017; 389: 519–27 | 0.71 [0.52;0.96] | 78% | 94% | 97% | 99% |
| J Clin Oncol 33:4138-4144 | 0.63 [0.44;0.9] | 90% | 97% | 99% | 99% |
| N Engl J Med 2016;375:1845-55 | 0.72 [0.58;0.88] | 84% | 98% | 100% | 100% |
| J Clin Oncol 33:3781-7 | 0.7 [0.5;0.98] | 78% | 93% | 96% | 98% |
| Lancet Oncol 2011; 12: 1204–13 | 0.65 [0.45;0.94] | 86% | 96% | 98% | 99% |
| Lancet 2016; 387: 1837–46 | 0.73 [0.53;0.99] | 72% | 91% | 95% | 98% |
| J Clin Oncol 28:562-569 | 0.47 [0.28;0.79] | 98% | 99% | 100% | 100% |
| N Engl J Med 2009; 361:1249-1259 | 0.74 [0.6;0.9] | 78% | 97% | 99% | 100% |
| N Engl J Med 2016;375:1856-67 | 0.7 [0.52;0.92] | 82% | 96% | 98% | 99% |
| N Engl J Med 2017; 377:352-360 | 0.62 [0.51;0.76] | 99% | 100% | 100% | 100% |
| N Engl J Med 2019; 380:1235-1246 | 0.71 [0.5;0.99] | 76% | 91% | 95% | 97% |
| N Engl J Med 2012; 367:107-114 | 0.54 [0.32;0.92] | 93% | 97% | 98% | 99% |
| Lancet Oncol 2014; 15: 78–86 | 0.67 [0.49;0.92] | 87% | 97% | 99% | 99% |
| Lancet 2014; 383: 31–39 | 0.78 [0.6;1] | 59% | 87% | 94% | 98% |
| N Engl J Med 2014; 370:997-1007 | 0.28 [0.09;0.86] | 97% | 98% | 98% | 99% |
| N Engl J Med 2018; 378:2078-2092 | 0.49 [0.38;0.64] | 100% | 100% | 100% | 100% |
| J Clin Oncol 29:2528-2533 | 0.56 [0.35;0.9] | 93% | 98% | 99% | 99% |
| Lancet 2014; 384: 665–73 | 0.86 [0.75;0.98] | 14% | 75% | 93% | 99% |
| Lancet Oncol 2014; 15: 1351–60 | 0.43 [0.26;0.69] | 99% | 100% | 100% | 100% |
| N Engl J Med 2014; 370:1101-1110 | 0.41 [0.23;0.74] | 99% | 100% | 100% | 100% |
| Lancet 2013; 382: 1021–28 | 0.76 [0.65;0.88] | 75% | 99% | 100% | 100% |
| Lancet Oncol 2016; 17: 1147–57 | 0.67 [0.51;0.88] | 90% | 98% | 99% | 100% |
| Lancet 2013; 381: 303–12 | 0.77 [0.64;0.94] | 65% | 94% | 98% | 100% |
| J Clin Oncol 31:1947-1953 | 0.74 [0.58;0.94] | 74% | 95% | 98% | 99% |
| J Clin Oncol 30:4344-4351 | 0.73 [0.55;0.96] | 74% | 93% | 97% | 99% |
| Lancet 2010; 376: 1164–74 | 0.67 [0.48;0.92] | 86% | 96% | 98% | 99% |
| JAMA Oncol. 2018;4(11):1569-1575 | 0.68 [0.54;0.87] | 91% | 99% | 100% | 100% |
| JAMA Oncol. doi:10.1001/jamaoncol.2019.0250 | 0.35 [0.26;0.48] | 100% | 100% | 100% | 100% |
| Lancet Oncol 2014; 15: 1065–75 | 0.77 [0.62;0.96] | 64% | 92% | 97% | 99% |
| Lancet 2016; 387: 1540–50 | 0.71 [0.58;0.88] | 87% | 99% | 100% | 100% |
| Lancet 2016; 387: 1540–50 | 0.61 [0.49;0.75] | 99% | 100% | 100% | 100% |
| N Engl J Med 2010; 363:711-723 | 0.66 [0.51;0.87] | 92% | 99% | 100% | 100% |
| N Engl J Med 2010; 363:711-723 | 0.68 [0.55;0.85] | 93% | 99% | 100% | 100% |
| Lancet Oncol 2018; 19: 1480–92 | 0.54 [0.44;0.67] | 100% | 100% | 100% | 100% |
| Lancet Oncol 2018; 19: 1480–92 | 0.65 [0.53;0.79] | 98% | 100% | 100% | 100% |
| J Clin Oncol 30:2441-2448 | 0.69 [0.5;0.96] | 81% | 94% | 97% | 99% |
| N Engl J Med 2018; 379:2220-2229 | 0.7 [0.54;0.91] | 85% | 97% | 99% | 100% |
| Lancet 2019; 393: 229–40 | 0.66 [0.46;0.95] | 85% | 95% | 98% | 99% |
| J Clin Oncol 27:242-249 | 0.26 [0.08;0.83] | 97% | 98% | 99% | 99% |
| J Clin Oncol 34:3609-3617 | 0.83 [0.69;0.99] | 34% | 81% | 93% | 98% |
| JAMA Oncol. 2018;4(4):483-492 | 0.73 [0.54;0.98] | 73% | 92% | 96% | 98% |
| Lancet 2016; 387: 1163–77 | 0.82 [0.69;0.97] | 38% | 86% | 95% | 99% |
| Lancet 2016; 387: 1163–77 | 0.78 [0.66;0.93] | 61% | 95% | 99% | 100% |
| N Engl J Med 2017; 377:338-351 | 0.63 [0.52;0.76] | 99% | 100% | 100% | 100% |
| JAMA 2012;307(12):1265-1272 | 0.45 [0.22;0.89] | 95% | 97% | 98% | 99% |
| J Clin Oncol 27:1177-1183 | 0.69 [0.5;0.97] | 81% | 94% | 97% | 99% |
| N Engl J Med 2011; 365:107-118 | 0.85 [0.74;0.99] | 21% | 78% | 93% | 99% |
| J Clin Oncol 30:1513-1518 | 0.66 [0.48;0.89] | 90% | 98% | 99% | 100% |
| Lancet 2017; 390: 2461–71 | 0.63 [0.51;0.78] | 99% | 100% | 100% | 100% |
| N Engl J Med 2017; 376:836-847 | 0.71 [0.55;0.93] | 81% | 96% | 99% | 100% |
| J Clin Oncol 28:1099-1105 | 0.56 [0.35;0.85] | 94% | 98% | 99% | 100% |
| N Engl J Med 2010; 363:411-422 | 0.78 [0.61;0.98] | 58% | 88% | 95% | 98% |
| Lancet 2009; 374: 1331–38 | 0.75 [0.57;0.98] | 68% | 91% | 96% | 98% |
| Lancet Oncol 2017; 18: 743–54 | 0.68 [0.54;0.85] | 92% | 99% | 100% | 100% |
| J Clin Oncol 33:1325-1333 | 0.59 [0.36;0.97] | 89% | 95% | 97% | 98% |
| J Clin Oncol 31:301-307 | 0.69 [0.48;0.99] | 77% | 92% | 95% | 97% |
| Lancet Oncol 2016; 17: 1579–89 | 0.73 [0.55;0.96] | 74% | 93% | 97% | 99% |
| N Engl J Med 2017; 377:1250-1260 | 0.5 [0.26;0.99] | 92% | 96% | 97% | 98% |
| J Clin Oncol 31:3219-3225 | 0.37 [0.22;0.62] | 100% | 100% | 100% | 100% |
| J Clin Oncol 31:3219-3225 | 0.41 [0.24;0.72] | 99% | 100% | 100% | 100% |
| JAMA. 2018;319(24):2486-2496 | 0.65 [0.51;0.83] | 95% | 100% | 100% | 100% |
| Lancet Oncol 2019; 20: 110–119 | 0.5 [0.33;0.75] | 99% | 100% | 100% | 100% |
| N Engl J Med 2014;371:1877-88 | 0.63 [0.42;0.94] | 88% | 96% | 98% | 99% |
| N Engl J Med 2017; 377:1813-1823 | 0.57 [0.43;0.79] | 98% | 100% | 100% | 100% |
| Lancet Oncol 2011; 12: 153–59 | 0.74 [0.58;0.94] | 74% | 94% | 98% | 99% |
| Lancet Oncol 2013; 14: 72–80 | 0.74 [0.61;0.9] | 78% | 98% | 99% | 100% |
| Lancet Oncol 2012; 13: 916–26 | 0.7 [0.52;0.93] | 82% | 95% | 98% | 99% |
| N Engl J Med 2017; 376:2147-2159 | 0.59 [0.39;0.9] | 92% | 98% | 99% | 99% |
| N Engl J Med 2013; 369:438-447 | 0.31 [0.1;0.91] | 95% | 97% | 98% | 98% |
| N Engl J Med 2015;372:1909-19 | 0.68 [0.58;0.81] | 97% | 100% | 100% | 100% |
| Lancet Oncol 2014; 15: 323–32 | 0.7 [0.57;0.87] | 89% | 99% | 100% | 100% |
| N Engl J Med 2012;366:1770-81 | 0.62 [0.4;0.95] | 88% | 96% | 97% | 99% |
| N Engl J Med 2012; 367:435-444 | 0.81 [0.65;1] | 46% | 83% | 93% | 97% |
| N Engl J Med 2012; 366:399-408 | 0.5 [0.25;0.99] | 91% | 95% | 97% | 98% |
| J Clin Oncol 28:2874-2880 | 0.76 [0.59;0.97] | 66% | 91% | 96% | 98% |
| J Clin Oncol 36:2244-2250 | 0.76 [0.58;0.99] | 64% | 89% | 95% | 98% |
| Lancet 2019; 393: 1819–30 | 0.81 [0.71;0.93] | 43% | 94% | 99% | 100% |
| Lancet 2019; 393: 1819–30 | 0.69 [0.56;0.85] | 92% | 99% | 100% | 100% |
| J Clin Oncol 28:3562-3569 | 0.67 [0.46;0.99] | 82% | 93% | 96% | 98% |
| Lancet 2010; 376: 1989–99 | 0.87 [0.77;0.99] | 10% | 70% | 91% | 99% |
| N Engl J Med 2015;373:1803-13 | 0.73 [0.57;0.93] | 77% | 95% | 98% | 99% |
| N Engl J Med 2018; 378:1277-1290 | 0.63 [0.44;0.89] | 91% | 98% | 99% | 100% |
| Lancet Oncol 2013; 14: 317–26 | 0.5 [0.27;0.93] | 93% | 97% | 98% | 99% |
| Lancet 2017; 389: 1011–24 | 0.82 [0.68;0.98] | 40% | 84% | 94% | 98% |
| JAMA. 2013;310(14):1473-1481 | 0.76 [0.61;0.95] | 67% | 93% | 98% | 99% |
| J Clin Oncol 32:2423-2429 | 0.66 [0.48;0.91] | 88% | 97% | 99% | 99% |
| N Engl J Med 2011; 364:205-214 | 0.57 [0.36;0.9] | 93% | 97% | 99% | 99% |
| Lancet Oncol 2015; 16: 928–36 | 0.78 [0.63;0.97] | 59% | 90% | 96% | 99% |
| J Clin Oncol 32:634-640 | 0.7 [0.52;0.92] | 82% | 96% | 98% | 99% |
| J Clin Oncol 31:1732-1739 | 0.65 [0.42;0.99] | 83% | 93% | 96% | 97% |
| Lancet 2010; 376: 2009–17 | 0.56 [0.36;0.87] | 94% | 98% | 99% | 99% |
| N Engl J Med 2013; 369:213-223 | 0.7 [0.55;0.88] | 87% | 98% | 99% | 100% |
| Lancet Oncol 2012; 13: 247–55 | 0.78 [0.64;0.96] | 59% | 92% | 97% | 99% |
| N Engl J Med 2018; 379:2040-2051 | 0.64 [0.49;0.85] | 94% | 99% | 100% | 100% |
| J Clin Oncol 31:426-432 | 0.52 [0.34;0.82] | 96% | 99% | 100% | 100% |
| N Engl J Med 2017;376:1027-37 | 0.67 [0.56;0.8] | 97% | 100% | 100% | 100% |
| Lancet 2009; 373: 1525–31 | 0.87 [0.76;1] | 11% | 69% | 90% | 98% |
| Lancet Oncol 2019; 20: 663–73 | 0.75 [0.58;0.97] | 69% | 92% | 96% | 99% |
| J Clin Oncol 36:3031-3039 | 0.76 [0.61;0.96] | 67% | 93% | 97% | 99% |
| Lancet 2011; 378: 1079–88 | 0.64 [0.52;0.78] | 98% | 100% | 100% | 100% |
| N Engl J Med 2011; 364:501-513 | 0.41 [0.19;0.89] | 96% | 98% | 98% | 99% |
| Lancet 2011; 378: 1858–67 | 0.44 [0.28;0.81] | 99% | 100% | 100% | 100% |
| N Engl J Med 2016;375:1823-33 | 0.6 [0.41;0.89] | 93% | 98% | 99% | 99% |
| Lancet Oncol 2011; 12: 1101–08 | 0.79 [0.69;0.9] | 57% | 97% | 100% | 100% |
| Lancet 2016; 387: 2402–11 | 0.51 [0.3;0.86] | 95% | 98% | 99% | 99% |
| N Engl J Med 2019; 380:1116-1127 | 0.53 [0.38;0.74] | 99% | 100% | 100% | 100% |
| Lancet 2017; 389: 255–65 | 0.73 [0.62;0.88] | 85% | 99% | 100% | 100% |
| Lancet Oncol 2018; 19: 1449–58 | 0.66 [0.51;0.85] | 93% | 99% | 100% | 100% |
| N Engl J Med 2011; 364:2517-2526 | 0.72 [0.59;0.87] | 85% | 99% | 100% | 100% |
| N Engl J Med 2015;372:30-9 | 0.69 [0.53;0.89] | 86% | 98% | 99% | 100% |
| N Engl J Med 2015; 372:320-330 | 0.42 [0.3;0.59] | 100% | 100% | 100% | 100% |
| N Engl J Med 2015;372:2521-32 | 0.63 [0.47;0.83] | 95% | 99% | 100% | 100% |
| N Engl J Med 2015;372:2521-32 | 0.69 [0.52;0.9] | 85% | 97% | 99% | 100% |
| J Clin Oncol 37:1159-1168 | 0.69 [0.49;0.97] | 80% | 94% | 97% | 98% |
| N Engl J Med 2013; 368:138-148 | 0.75 [0.61;0.93] | 73% | 95% | 99% | 100% |
| J Clin Oncol 31:448-455 | 0.7 [0.57;0.85] | 91% | 99% | 100% | 100% |
| J Clin Oncol 29:4387-4393 | 0.67 [0.54;0.83] | 95% | 100% | 100% | 100% |
| J Clin Oncol 30:172-178 | 0.63 [0.43;0.92] | 89% | 97% | 98% | 99% |
| N Engl J Med 2012; 367:1187-1197 | 0.63 [0.53;0.75] | 100% | 100% | 100% | 100% |
| N Engl J Med 2018; 379:2108-2121 | 0.62 [0.45;0.86] | 94% | 99% | 100% | 100% |
| J Clin Oncol 33:3733-3740 | 0.83 [0.7;0.99] | 34% | 82% | 94% | 98% |
| Lancet 2016; 387: 1629–37 | 0.77 [0.62;0.95] | 64% | 92% | 97% | 99% |
| N Engl J Med 2018;378:1107-20 | 0.48 [0.25;0.9] | 94% | 97% | 98% | 99% |
| J Clin Oncol 28:4581-4586 | 0.44 [0.22;0.86] | 96% | 98% | 99% | 99% |
| N Engl J Med 2017; 376:417-428 | 0.77 [0.59;0.99] | 61% | 88% | 94% | 98% |
| Lancet Oncol 2018; 19: 1437–48 | 0.69 [0.56;0.85] | 92% | 99% | 100% | 100% |
| JAMA Oncol. 2018;4(4):506-513 | 0.82 [0.68;0.98] | 39% | 84% | 94% | 98% |
| N Engl J Med 2011; 365:1273-1283 | 0.63 [0.48;0.82] | 96% | 100% | 100% | 100% |
| N Engl J Med 2011; 365:1273-1283 | 0.77 [0.6;0.98] | 62% | 90% | 95% | 98% |
| J Clin Oncol 30:2327-2333 | 0.76 [0.62;0.91] | 71% | 96% | 99% | 100% |
| N Engl J Med 2018; 378:2288-2301 | 0.78 [0.64;0.96] | 60% | 92% | 97% | 99% |
| Lancet Oncol 2015; 16: 897–907 | 0.81 [0.69;0.95] | 44% | 90% | 97% | 100% |
| N Engl J Med 2018; 378:113-125 | 0.63 [0.45;0.88] | 92% | 98% | 99% | 100% |
| J Clin Oncol 27:1788-1793 | 0.41 [0.22;0.76] | 98% | 99% | 100% | 100% |
| N Engl J Med 2015; 372:142-152 | 0.79 [0.63;0.99] | 54% | 87% | 95% | 98% |
| J Clin Oncol 29:4498-4504 | 0.72 [0.55;0.94] | 78% | 95% | 98% | 99% |
| N Engl J Med 2017; 377:454-464 | 0.78 [0.63;0.96] | 60% | 91% | 97% | 99% |
| Lancet Oncol 2009; 10: 459–66 | 0.49 [0.32;0.76] | 99% | 100% | 100% | 100% |
| JAMA. 2015;314(23):2535-2543 | 0.74 [0.56;0.98] | 71% | 92% | 96% | 98% |
| N Engl J Med 2015; 373:737-746 | 0.61 [0.47;0.8] | 98% | 100% | 100% | 100% |
| Lancet Oncol 2015; 16: 499–508 | 0.84 [0.73;0.98] | 24% | 81% | 94% | 99% |
| Lancet 2016; 388: 488–97 | 0.46 [0.3;0.71] | 100% | 100% | 100% | 100% |
| N Engl J Med 2014;370:734-43 | 0.71 [0.54;0.95] | 79% | 95% | 98% | 99% |
| Lancet Oncol 2015; 16: 763–74 | 0.84 [0.74;0.96] | 23% | 85% | 97% | 100% |
| Lancet Oncol 2015; 16: 1493–1505 | 0.79 [0.63;0.99] | 54% | 87% | 95% | 98% |
| J Clin Oncol 27:2938-2945 | 0.83 [0.69;0.99] | 35% | 81% | 93% | 98% |
| J Clin Oncol 35:3030-3038 | 0.68 [0.51;0.9] | 87% | 97% | 99% | 100% |
| N Engl J Med 2010; 362:1273-1281 | 0.64 [0.52;0.8] | 98% | 100% | 100% | 100% |
| J Clin Oncol 29:2011-2019 | 0.88 [0.77;1] | 7% | 65% | 89% | 98% |
| J Clin Oncol 30:3499-3506 | 0.82 [0.71;0.94] | 38% | 92% | 98% | 100% |
| N Engl J Med 2012; 366:2074-2084 | 0.66 [0.5;0.87] | 92% | 99% | 100% | 100% |
| N Engl J Med 2018; 378:230-240 | 0.67 [0.48;0.94] | 85% | 96% | 98% | 99% |
| N Engl J Med 2012; 367:1783-1791 | 0.68 [0.55;0.85] | 93% | 99% | 100% | 100% |
| N Engl J Med 2012; 366:799-807 | 0.5 [0.25;0.98] | 91% | 95% | 97% | 98% |
| N Engl J Med 2013;369:1691-703 | 0.72 [0.62;0.83] | 92% | 100% | 100% | 100% |
| Lancet 2016; 387: 545–57 | 0.67 [0.49;0.92] | 87% | 97% | 99% | 99% |
| Lancet 2011; 378: 2104–11 | 0.77 [0.61;0.98] | 63% | 90% | 96% | 99% |
| Lancet Oncol 2016; 17: 943–55 | 0.48 [0.29;0.8] | 98% | 99% | 100% | 100% |
| J Clin Oncol 36:3110-3119 | 0.63 [0.41;0.95] | 87% | 95% | 97% | 98% |
| Lancet 2009; 373: 301–08 | 0.68 [0.52;0.89] | 88% | 98% | 99% | 100% |
| Lancet Oncol 2014; 15: 1224–35 | 0.81 [0.68;0.96] | 46% | 89% | 97% | 99% |
| J Clin Oncol 36:2926-2934 | 0.63 [0.41;0.97] | 86% | 94% | 97% | 98% |
| Lancet Oncol 2013; 14: 777–86 | 0.79 [0.64;0.99] | 55% | 88% | 95% | 98% |
| J Clin Oncol 36:350-358 | 0.79 [0.62;0.99] | 54% | 86% | 94% | 98% |
| J Clin Oncol 36:2796-2803 | 0.71 [0.53;0.96] | 79% | 94% | 97% | 99% |
| J Clin Oncol 31:2128-2135 | 0.59 [0.36;0.97] | 89% | 95% | 97% | 98% |
| J Clin Oncol 29:1715-1721 | 0.69 [0.5;0.95] | 81% | 95% | 97% | 99% |
| J Clin Oncol 31:1931-1938 | 0.54 [0.33;0.89] | 94% | 98% | 99% | 99% |
| JAMA Oncol. 2018;4(5):661-669 | 0.61 [0.38;0.98] | 87% | 95% | 97% | 98% |
| Lancet Oncol 2012; 13: 993–1001 | 0.56 [0.29;0.81] | 91% | 96% | 98% | 98% |
| Lancet 2016; 387: 1405–14 | 0.77 [0.62;0.95] | 64% | 93% | 97% | 99% |
| Lancet Oncol 2015; 16: 320–27 | 0.4 [0.21;0.76] | 98% | 99% | 100% | 100% |
| Lancet Oncol 2017; 18: 297–311 | 0.62 [0.42;0.92] | 90% | 97% | 98% | 99% |
| Lancet 2016; 388: 1883–92 | 0.62 [0.45;0.84] | 95% | 99% | 100% | 100% |
| Lancet Oncol 2017; 18: 371–83 | 0.63 [0.45;0.88] | 92% | 98% | 99% | 100% |
| N Engl J Med 2019; 381:1124-1135 | 0.43 [0.24;0.77] | 98% | 99% | 100% | 100% |
| J Clin Oncol 33:2197-2204 | 0.68 [0.5;0.93] | 85% | 96% | 98% | 99% |
| Lancet Oncol 2019; 20: 282–96 | 0.71 [0.53;0.95] | 79% | 95% | 98% | 99% |
| J Clin Oncol 31:2849-2853 | 0.62 [0.46;0.83] | 96% | 99% | 100% | 100% |

Below we show the results from the first sensitivity analysis, where we used a prior $\mu_{i}\sim N(0, \sigma^{2}=10)$ for the effect (log of the hazard ratio). Results were very similar to the primary analysis.

eTable 11: Analysis results from 234 estimates of HR in oncology trials using a prior $logHR\sim N(0, \sigma^{2}=10)$

| **Study** | **Estimated Effect size**  **[95% CrI]** | $\boldsymbol{P(HR<0.80)}$ | $\boldsymbol{P(HR<0.90)}$ | $\boldsymbol{P(HR<0.95)}$ | $\boldsymbol{P(HR<1.00)}$ |
| --- | --- | --- | --- | --- | --- |
| JAMA. 2010;304(19):2154-2160 | 0.49 [0.3;0.8] | 97.4% | 99.3% | 99.6% | 99.8% |
| N Engl J Med 2018; 379:54-63 | 0.76 [0.63;0.92] | 70.1% | 95.9% | 98.9% | 99.8% |
| Lancet 2019; 393: 1948–57 | 0.77 [0.64;0.93] | 65% | 94.5% | 98.3% | 99.6% |
| J Clin Oncol 27:5062-5067 | 0.84 [0.72;0.98] | 26.9% | 80.9% | 94.1% | 98.7% |
| J Clin Oncol 34:972-979 | 0.69 [0.53;0.9] | 86.5% | 97.5% | 99.1% | 99.7% |
| J Clin Oncol 27:3109-3116 | 0.84 [0.71;1] | 29.1% | 78.7% | 92.2% | 97.8% |
| N Engl J Med 2018; 379:2342-235 | 0.68 [0.47;0.99] | 79.6% | 92.7% | 95.8% | 97.8% |
| Lancet Oncol 2016; 17: 1248–60 | 0.7 [0.55;0.9] | 85.5% | 97.7% | 99.2% | 99.7% |
| Lancet Oncol 2017; 18: 611–22 | 0.84 [0.71;1] | 28.9% | 78.3% | 91.8% | 97.6% |
| Lancet Oncol 2012; 13: 671–78 | 0.68 [0.47;0.98] | 80.8% | 93.2% | 96.3% | 98% |
| J Clin Oncol 30:2718-2724 | 0.45 [0.25;0.83] | 96.8% | 98.8% | 99.2% | 99.5% |
| Lancet Oncol 2019; 20: 494–503 | 0.53 [0.3;0.95] | 91.9% | 96.3% | 97.6% | 98.4% |
| Lancet 2010; 376: 687–97 | 0.74 [0.6;0.91] | 76.8% | 96.7% | 99.1% | 99.8% |
| Lancet 2012; 379: 315–21 | 0.72 [0.52;0.99] | 73.5% | 91.1% | 95.4% | 97.7% |
| J Clin Oncol 33:3858-3865 | 0.56 [0.36;0.89] | 93.6% | 97.8% | 98.8% | 99.4% |
| N Engl J Med 2012; 366:109-119 | 0.64 [0.47;0.88] | 91.7% | 98.3% | 99.4% | 99.8% |
| Lancet Oncol 2015; 16: 1037–48 | 0.65 [0.49;0.87] | 91.6% | 98.5% | 99.5% | 99.8% |
| N Engl J Med 2014; 371:424-433 | 0.71 [0.6;0.84] | 91.9% | 99.8% | 100% | 100% |
| J Clin Oncol 27:4454-4461 | 0.78 [0.61;0.99] | 58.3% | 87.8% | 94.5% | 97.9% |
| N Engl J Med 2017; 376:1015-1026 | 0.73 [0.59;0.91] | 79.4% | 97% | 99.1% | 99.8% |
| N Engl J Med 2014; 371:906-917 | 0.78 [0.64;0.95] | 59.7% | 91.4% | 97.2% | 99.2% |
| Lancet Oncol 2013; 14: 29–37 | 0.81 [0.69;0.95] | 43.4% | 90.7% | 97.8% | 99.6% |
| J Clin Oncol 30:2585-2592 | 0.74 [0.57;0.96] | 71.9% | 92.7% | 96.7% | 98.7% |
| Lancet Oncol 2010; 11: 1066–73 | 0.6 [0.45;0.8] | 97.3% | 99.7% | 99.9% | 100% |
| Lancet Oncol 2010; 11: 21–28 | 0.73 [0.56;0.95] | 75.3% | 93.9% | 97.4% | 99% |
| N Engl J Med 2015;373:1627-39 | 0.73 [0.6;0.89] | 82.1% | 98.1% | 99.5% | 99.9% |
| N Engl J Med 2015;373:123-35 | 0.59 [0.44;0.79] | 97.8% | 99.8% | 99.9% | 100% |
| Lancet 2017; 389: 56–66 | 0.63 [0.5;0.79] | 97.9% | 99.9% | 100% | 100% |
| N Engl J Med 2016;374:1344-55 | 0.59 [0.42;0.83] | 95.9% | 99.2% | 99.7% | 99.9% |
| N Engl J Med 2015; 373:2425-2437 | 0.17 [0.05;0.56] | 99.4% | 99.7% | 99.8% | 99.8% |
| J Clin Oncol 28:29-34 | 0.78 [0.61;1] | 58% | 87.7% | 94.4% | 97.7% |
| N Engl J Med 2014; 371:213-223 | 0.43 [0.24;0.79] | 97.8% | 99.2% | 99.5% | 99.7% |
| Lancet Oncol 2010; 11: 521–29 | 0.81 [0.7;0.94] | 43.6% | 91.1% | 97.9% | 99.6% |
| Lancet 2012; 379: 1508–16 | 0.69 [0.49;0.98] | 79.7% | 93.3% | 96.5% | 98.2% |
| Lancet Oncol 2018; 19: 1247–58 | 0.65 [0.45;0.94] | 87% | 96.1% | 97.9% | 99% |
| Lancet Oncol 2009; 10: 25–34 | 0.68 [0.5;0.93] | 84.4% | 96.1% | 98.2% | 99.3% |
| J Clin Oncol 36:2259-2266 | 0.67 [0.47;0.96] | 83.5% | 94.8% | 97.3% | 98.6% |
| N Engl J Med 2015;373:1814-23 | 0.67 [0.51;0.89] | 89.5% | 98.1% | 99.3% | 99.8% |
| Lancet 2009; 374: 1432–40 | 0.79 [0.65;0.95] | 55.1% | 91.3% | 97.2% | 99.3% |
| Lancet 2019; 393: 156–67 | 0.8 [0.65;0.98] | 50% | 86.9% | 94.9% | 98.4% |
| N Engl J Med 2011; 364:1817-1825 | 0.57 [0.45;0.73] | 99.7% | 100% | 100% | 100% |
| N Engl J Med 2018; 379:2395-2406 | 0.64 [0.48;0.86] | 93.3% | 98.9% | 99.6% | 99.9% |
| J Clin Oncol 29:3247-3254 | 0.66 [0.46;0.94] | 85.6% | 95.6% | 97.8% | 98.9% |
| Lancet 2011; 377: 914–23 | 0.81 [0.66;0.99] | 45.1% | 84.4% | 93.9% | 97.8% |
| Lancet Oncol 2015; 16: 1306–15 | 0.81 [0.66;0.99] | 45.7% | 84.4% | 93.7% | 97.8% |
| (online) | 0.67 [0.52;0.86] | 91.9% | 99% | 99.7% | 99.9% |
| Lancet 2010; 376: 1147–54 | 0.7 [0.59;0.83] | 93.7% | 99.8% | 100% | 100% |
| N Engl J Med 2011; 364:1995-2005 | 0.65 [0.54;0.78] | 99% | 100% | 100% | 100% |
| Lancet 2015; 385: 1863–72 | 0.65 [0.51;0.84] | 94.9% | 99.5% | 99.9% | 100% |
| Lancet Oncol 2017; 18: 1327–37 | 0.79 [0.65;0.97] | 54.5% | 89.7% | 96.3% | 99% |
| N Engl J Med 2013; 369:1023-1034 | 0.78 [0.62;0.99] | 58.5% | 88.4% | 94.9% | 98% |
| Lancet Oncol 2016; 17: 727–37 | 0.55 [0.31;1.01] | 88.5% | 94.4% | 96.2% | 97.4% |
| J Clin Oncol 29:1678-1685 | 0.68 [0.49;0.95] | 83.1% | 95.1% | 97.6% | 98.9% |
| Lancet Oncol 2018; 19: 1315–27 | 0.76 [0.58;0.99] | 64.6% | 89.5% | 95.1% | 97.9% |
| Lancet Oncol 2018; 19: 1315–27 | 0.61 [0.47;0.79] | 98% | 99.9% | 100% | 100% |
| Lancet 2017; 389: 519–27 | 0.71 [0.52;0.96] | 78.3% | 93.9% | 97.1% | 98.7% |
| J Clin Oncol 33:4138-4144 | 0.63 [0.44;0.9] | 90.2% | 97.4% | 98.8% | 99.4% |
| N Engl J Med 2016;375:1845-55 | 0.72 [0.59;0.89] | 83.5% | 98.1% | 99.5% | 99.9% |
| J Clin Oncol 33:3781-7 | 0.7 [0.5;0.98] | 78.3% | 92.8% | 96.2% | 98.2% |
| Lancet Oncol 2011; 12: 1204–13 | 0.65 [0.45;0.94] | 86.2% | 95.6% | 97.7% | 98.9% |
| Lancet 2016; 387: 1837–46 | 0.73 [0.54;1] | 71.4% | 90.6% | 95.2% | 97.6% |
| J Clin Oncol 28:562-569 | 0.47 [0.28;0.79] | 97.8% | 99.4% | 99.6% | 99.8% |
| N Engl J Med 2009; 361:1249-1259 | 0.74 [0.6;0.91] | 77.5% | 97% | 99.2% | 99.8% |
| N Engl J Med 2016;375:1856-67 | 0.7 [0.53;0.93] | 81.6% | 95.7% | 98.1% | 99.2% |
| N Engl J Med 2017; 377:352-360 | 0.62 [0.51;0.76] | 99.3% | 100% | 100% | 100% |
| N Engl J Med 2019; 380:1235-1246 | 0.71 [0.51;1] | 74.8% | 91% | 95.2% | 97.5% |
| N Engl J Med 2012; 367:107-114 | 0.54 [0.32;0.92] | 92.7% | 97% | 98.1% | 98.8% |
| Lancet Oncol 2014; 15: 78–86 | 0.67 [0.49;0.92] | 86.4% | 96.6% | 98.5% | 99.4% |
| Lancet 2014; 383: 31–39 | 0.78 [0.6;1] | 59.6% | 87.7% | 94.3% | 97.6% |
| N Engl J Med 2014; 370:997-1007 | 0.29 [0.1;0.89] | 96.2% | 97.6% | 98.1% | 98.5% |
| N Engl J Med 2018; 378:2078-2092 | 0.49 [0.38;0.64] | 100% | 100% | 100% | 100% |
| J Clin Oncol 29:2528-2533 | 0.56 [0.35;0.9] | 93% | 97.6% | 98.6% | 99.2% |
| Lancet 2014; 384: 665–73 | 0.86 [0.75;0.98] | 14.4% | 75% | 92.9% | 98.6% |
| Lancet Oncol 2014; 15: 1351–60 | 0.43 [0.26;0.71] | 99.3% | 99.8% | 99.9% | 100% |
| N Engl J Med 2014; 370:1101-1110 | 0.41 [0.23;0.74] | 98.7% | 99.6% | 99.8% | 99.9% |
| Lancet 2013; 382: 1021–28 | 0.76 [0.65;0.88] | 74.6% | 98.6% | 99.8% | 100% |
| Lancet Oncol 2016; 17: 1147–57 | 0.67 [0.51;0.88] | 89.7% | 98.3% | 99.4% | 99.8% |
| Lancet 2013; 381: 303–12 | 0.77 [0.64;0.93] | 65% | 94.7% | 98.6% | 99.7% |
| J Clin Oncol 31:1947-1953 | 0.74 [0.58;0.94] | 73.5% | 94.4% | 97.7% | 99.2% |
| J Clin Oncol 30:4344-4351 | 0.73 [0.55;0.96] | 74.2% | 93.1% | 97% | 98.7% |
| Lancet 2010; 376: 1164–74 | 0.67 [0.48;0.93] | 85.9% | 96.3% | 98.2% | 99.2% |
| JAMA Oncol. 2018;4(11):1569-1575 | 0.68 [0.54;0.86] | 90.9% | 98.9% | 99.7% | 99.9% |
| JAMA Oncol. doi:10.1001/jamaoncol.2019.0250 | 0.35 [0.26;0.48] | 100% | 100% | 100% | 100% |
| Lancet Oncol 2014; 15: 1065–75 | 0.77 [0.62;0.96] | 63.3% | 92% | 97% | 99% |
| Lancet 2016; 387: 1540–50 | 0.71 [0.58;0.87] | 86.9% | 98.7% | 99.7% | 99.9% |
| Lancet 2016; 387: 1540–50 | 0.61 [0.49;0.75] | 99.4% | 100% | 100% | 100% |
| N Engl J Med 2010; 363:711-723 | 0.66 [0.51;0.86] | 92.1% | 98.9% | 99.6% | 99.9% |
| N Engl J Med 2010; 363:711-723 | 0.68 [0.55;0.84] | 93% | 99.5% | 99.9% | 100% |
| Lancet Oncol 2018; 19: 1480–92 | 0.54 [0.44;0.67] | 100% | 100% | 100% | 100% |
| Lancet Oncol 2018; 19: 1480–92 | 0.65 [0.53;0.79] | 97.9% | 99.9% | 100% | 100% |
| J Clin Oncol 30:2441-2448 | 0.69 [0.5;0.96] | 80.7% | 94.3% | 97.2% | 98.6% |
| N Engl J Med 2018; 379:2220-2229 | 0.7 [0.54;0.91] | 84.3% | 97.1% | 98.9% | 99.6% |
| Lancet 2019; 393: 229–40 | 0.66 [0.46;0.95] | 85% | 95.2% | 97.5% | 98.8% |
| J Clin Oncol 27:242-249 | 0.27 [0.09;0.85] | 96.9% | 98.1% | 98.4% | 98.8% |
| J Clin Oncol 34:3609-3617 | 0.83 [0.69;0.99] | 34.3% | 81% | 92.8% | 97.8% |
| JAMA Oncol. 2018;4(4):483-492 | 0.73 [0.54;0.98] | 72.5% | 91.4% | 95.7% | 98% |
| Lancet 2016; 387: 1163–77 | 0.82 [0.69;0.97] | 38.9% | 85.6% | 95.3% | 98.8% |
| Lancet 2016; 387: 1163–77 | 0.78 [0.66;0.92] | 61.2% | 95% | 98.8% | 99.8% |
| N Engl J Med 2017; 377:338-351 | 0.63 [0.52;0.76] | 99.2% | 100% | 100% | 100% |
| JAMA 2012;307(12):1265-1272 | 0.45 [0.23;0.91] | 94.5% | 97.3% | 98.1% | 98.7% |
| J Clin Oncol 27:1177-1183 | 0.69 [0.49;0.96] | 80.8% | 94.3% | 97% | 98.6% |
| N Engl J Med 2011; 365:107-118 | 0.85 [0.73;0.98] | 21% | 77.9% | 93.3% | 98.6% |
| J Clin Oncol 30:1513-1518 | 0.66 [0.49;0.89] | 89.7% | 97.8% | 99.2% | 99.7% |
| Lancet 2017; 390: 2461–71 | 0.63 [0.51;0.78] | 98.5% | 100% | 100% | 100% |
| N Engl J Med 2017; 376:836-847 | 0.71 [0.55;0.92] | 81.3% | 96.2% | 98.5% | 99.5% |
| J Clin Oncol 28:1099-1105 | 0.56 [0.36;0.88] | 94.2% | 98.2% | 99.1% | 99.5% |
| N Engl J Med 2010; 363:411-422 | 0.78 [0.62;0.99] | 58% | 88.2% | 94.8% | 98% |
| Lancet 2009; 374: 1331–38 | 0.75 [0.57;0.98] | 68.2% | 90.8% | 95.7% | 98.1% |
| Lancet Oncol 2017; 18: 743–54 | 0.68 [0.54;0.85] | 92.1% | 99.2% | 99.8% | 99.9% |
| J Clin Oncol 33:1325-1333 | 0.59 [0.36;0.97] | 88.2% | 95.1% | 97% | 98.1% |
| J Clin Oncol 31:301-307 | 0.69 [0.48;1] | 77.8% | 91.8% | 95.4% | 97.5% |
| Lancet Oncol 2016; 17: 1579–89 | 0.73 [0.55;0.97] | 74% | 93% | 96.8% | 98.5% |
| N Engl J Med 2017; 377:1250-1260 | 0.5 [0.26;0.98] | 91.3% | 95.7% | 97% | 97.9% |
| J Clin Oncol 31:3219-3225 | 0.37 [0.22;0.62] | 99.8% | 100% | 100% | 100% |
| J Clin Oncol 31:3219-3225 | 0.41 [0.24;0.72] | 99.1% | 99.7% | 99.9% | 99.9% |
| JAMA. 2018;319(24):2486-2496 | 0.65 [0.51;0.83] | 95.1% | 99.6% | 99.9% | 100% |
| Lancet Oncol 2019; 20: 110–119 | 0.5 [0.33;0.75] | 98.8% | 99.8% | 99.9% | 100% |
| N Engl J Med 2014;371:1877-88 | 0.63 [0.42;0.94] | 87.7% | 95.9% | 97.7% | 98.8% |
| N Engl J Med 2017; 377:1813-1823 | 0.57 [0.42;0.77] | 98.6% | 99.8% | 99.9% | 100% |
| Lancet Oncol 2011; 12: 153–59 | 0.74 [0.58;0.94] | 73.7% | 94.5% | 97.9% | 99.3% |
| Lancet Oncol 2013; 14: 72–80 | 0.74 [0.61;0.9] | 78.3% | 97.5% | 99.4% | 99.9% |
| Lancet Oncol 2012; 13: 916–26 | 0.7 [0.52;0.94] | 81.4% | 95.4% | 98% | 99.2% |
| N Engl J Med 2017; 376:2147-2159 | 0.59 [0.39;0.9] | 92.1% | 97.6% | 98.7% | 99.4% |
| N Engl J Med 2013; 369:438-447 | 0.32 [0.11;0.94] | 95.1% | 97% | 97.6% | 98.1% |
| N Engl J Med 2015;372:1909-19 | 0.68 [0.58;0.8] | 97.3% | 100% | 100% | 100% |
| Lancet Oncol 2014; 15: 323–32 | 0.7 [0.57;0.87] | 89.2% | 99% | 99.8% | 100% |
| N Engl J Med 2012;366:1770-81 | 0.62 [0.4;0.96] | 87.5% | 95.3% | 97.3% | 98.4% |
| N Engl J Med 2012; 367:435-444 | 0.81 [0.66;1] | 45.5% | 83.2% | 92.6% | 97.2% |
| N Engl J Med 2012; 366:399-408 | 0.5 [0.25;1] | 90.5% | 95.1% | 96.4% | 97.4% |
| J Clin Oncol 28:2874-2880 | 0.76 [0.6;0.97] | 65.5% | 91% | 96.3% | 98.6% |
| J Clin Oncol 36:2244-2250 | 0.76 [0.58;0.99] | 64.2% | 89% | 94.8% | 97.8% |
| Lancet 2019; 393: 1819–30 | 0.81 [0.71;0.93] | 43% | 93.7% | 98.9% | 99.9% |
| Lancet 2019; 393: 1819–30 | 0.69 [0.56;0.85] | 91.7% | 99.3% | 99.9% | 100% |
| J Clin Oncol 28:3562-3569 | 0.67 [0.46;0.98] | 81.3% | 93.2% | 96.2% | 97.9% |
| Lancet 2010; 376: 1989–99 | 0.87 [0.77;0.99] | 9.7% | 69.8% | 91.5% | 98.5% |
| N Engl J Med 2015;373:1803-13 | 0.73 [0.57;0.93] | 76.1% | 95.3% | 98.2% | 99.4% |
| N Engl J Med 2018; 378:1277-1290 | 0.63 [0.44;0.89] | 90.8% | 97.7% | 98.9% | 99.5% |
| Lancet Oncol 2013; 14: 317–26 | 0.5 [0.27;0.93] | 92.9% | 96.8% | 97.9% | 98.6% |
| Lancet 2017; 389: 1011–24 | 0.82 [0.68;0.99] | 39.4% | 84.2% | 94.3% | 98.4% |
| JAMA. 2013;310(14):1473-1481 | 0.76 [0.61;0.95] | 67.2% | 93.3% | 97.5% | 99.2% |
| J Clin Oncol 32:2423-2429 | 0.66 [0.48;0.91] | 88.1% | 97.1% | 98.7% | 99.5% |
| N Engl J Med 2011; 364:205-214 | 0.57 [0.36;0.9] | 92.5% | 97.4% | 98.6% | 99.2% |
| Lancet Oncol 2015; 16: 928–36 | 0.78 [0.63;0.97] | 59.3% | 90% | 96.4% | 98.7% |
| J Clin Oncol 32:634-640 | 0.7 [0.52;0.93] | 82% | 95.8% | 98.2% | 99.3% |
| J Clin Oncol 31:1732-1739 | 0.65 [0.42;1] | 82.8% | 93.1% | 95.9% | 97.6% |
| Lancet 2010; 376: 2009–17 | 0.56 [0.36;0.88] | 94.2% | 98.1% | 99% | 99.5% |
| N Engl J Med 2013; 369:213-223 | 0.7 [0.55;0.89] | 86.7% | 98.2% | 99.5% | 99.9% |
| Lancet Oncol 2012; 13: 247–55 | 0.78 [0.64;0.96] | 59.3% | 91.7% | 97.1% | 99.3% |
| N Engl J Med 2018; 379:2040-2051 | 0.64 [0.49;0.84] | 94.4% | 99.2% | 99.8% | 99.9% |
| J Clin Oncol 31:426-432 | 0.53 [0.34;0.82] | 96.8% | 99.1% | 99.5% | 99.7% |
| N Engl J Med 2017;376:1027-37 | 0.67 [0.56;0.8] | 97.5% | 100% | 100% | 100% |
| Lancet 2009; 373: 1525–31 | 0.87 [0.76;1] | 10.7% | 68.5% | 90% | 97.8% |
| Lancet Oncol 2019; 20: 663–73 | 0.75 [0.58;0.97] | 68.4% | 91.5% | 96.2% | 98.5% |
| J Clin Oncol 36:3031-3039 | 0.76 [0.61;0.96] | 67.2% | 92.8% | 97.2% | 99.1% |
| Lancet 2011; 378: 1079–88 | 0.64 [0.52;0.78] | 98.5% | 100% | 100% | 100% |
| N Engl J Med 2011; 364:501-513 | 0.41 [0.19;0.89] | 95.4% | 97.6% | 98.3% | 98.8% |
| Lancet 2011; 378: 1858–67 | 0.44 [0.26;0.75] | 98.5% | 99.6% | 99.8% | 99.9% |
| N Engl J Med 2016;375:1823-33 | 0.6 [0.41;0.89] | 92.6% | 97.9% | 98.9% | 99.5% |
| Lancet Oncol 2011; 12: 1101–08 | 0.79 [0.69;0.9] | 57.4% | 97.4% | 99.7% | 100% |
| Lancet 2016; 387: 2402–11 | 0.51 [0.3;0.87] | 95.2% | 98.2% | 98.9% | 99.3% |
| N Engl J Med 2019; 380:1116-1127 | 0.53 [0.38;0.74] | 99.2% | 99.9% | 100% | 100% |
| Lancet 2017; 389: 255–65 | 0.73 [0.61;0.87] | 84.8% | 99.1% | 99.8% | 100% |
| Lancet Oncol 2018; 19: 1449–58 | 0.66 [0.51;0.85] | 93.1% | 99.2% | 99.8% | 100% |
| N Engl J Med 2011; 364:2517-2526 | 0.72 [0.59;0.87] | 85.7% | 98.8% | 99.8% | 100% |
| N Engl J Med 2015;372:30-9 | 0.69 [0.53;0.89] | 86.6% | 97.7% | 99.2% | 99.8% |
| N Engl J Med 2015; 372:320-330 | 0.42 [0.3;0.59] | 100% | 100% | 100% | 100% |
| N Engl J Med 2015;372:2521-32 | 0.63 [0.48;0.84] | 95.1% | 99.3% | 99.8% | 99.9% |
| N Engl J Med 2015;372:2521-32 | 0.69 [0.52;0.91] | 85.4% | 97.1% | 98.8% | 99.6% |
| J Clin Oncol 37:1159-1168 | 0.69 [0.49;0.97] | 80.2% | 93.6% | 96.7% | 98.3% |
| N Engl J Med 2013; 368:138-148 | 0.75 [0.61;0.93] | 72.8% | 95.4% | 98.6% | 99.6% |
| J Clin Oncol 31:448-455 | 0.7 [0.57;0.85] | 91.1% | 99.4% | 99.9% | 100% |
| J Clin Oncol 29:4387-4393 | 0.67 [0.54;0.83] | 95% | 99.6% | 99.9% | 100% |
| J Clin Oncol 30:172-178 | 0.63 [0.43;0.92] | 89.1% | 96.7% | 98.3% | 99.1% |
| N Engl J Med 2012; 367:1187-1197 | 0.63 [0.53;0.75] | 99.7% | 100% | 100% | 100% |
| N Engl J Med 2018; 379:2108-2121 | 0.62 [0.45;0.86] | 93.6% | 98.7% | 99.5% | 99.8% |
| J Clin Oncol 33:3733-3740 | 0.83 [0.7;0.99] | 34.1% | 82.4% | 93.8% | 98.3% |
| Lancet 2016; 387: 1629–37 | 0.77 [0.62;0.95] | 63.6% | 92.5% | 97.4% | 99.2% |
| N Engl J Med 2018;378:1107-20 | 0.49 [0.26;0.91] | 93.9% | 97.2% | 98.1% | 98.8% |
| J Clin Oncol 28:4581-4586 | 0.44 [0.23;0.87] | 95.5% | 98% | 98.7% | 99.1% |
| N Engl J Med 2017; 376:417-428 | 0.77 [0.6;1] | 61% | 88.1% | 94.5% | 97.6% |
| Lancet Oncol 2018; 19: 1437–48 | 0.69 [0.56;0.85] | 91.8% | 99.3% | 99.9% | 100% |
| JAMA Oncol. 2018;4(4):506-513 | 0.82 [0.68;0.98] | 39.6% | 83.8% | 94.2% | 98.4% |
| N Engl J Med 2011; 365:1273-1283 | 0.63 [0.48;0.82] | 95.8% | 99.5% | 99.8% | 100% |
| N Engl J Med 2011; 365:1273-1283 | 0.77 [0.6;0.98] | 61.8% | 89.3% | 95.4% | 98.2% |
| J Clin Oncol 30:2327-2333 | 0.76 [0.63;0.92] | 70.9% | 96.1% | 99% | 99.8% |
| N Engl J Med 2018; 378:2288-2301 | 0.78 [0.64;0.96] | 59.4% | 91.7% | 97.2% | 99.3% |
| Lancet Oncol 2015; 16: 897–907 | 0.81 [0.69;0.95] | 44% | 90.1% | 97.4% | 99.5% |
| N Engl J Med 2018; 378:113-125 | 0.63 [0.45;0.88] | 91.9% | 98.1% | 99.2% | 99.6% |
| J Clin Oncol 27:1788-1793 | 0.41 [0.22;0.77] | 98.3% | 99.3% | 99.6% | 99.7% |
| N Engl J Med 2015; 372:142-152 | 0.79 [0.63;0.99] | 54.4% | 87% | 94.5% | 97.9% |
| J Clin Oncol 29:4498-4504 | 0.72 [0.55;0.94] | 78.4% | 95.1% | 97.9% | 99.2% |
| N Engl J Med 2017; 377:454-464 | 0.78 [0.63;0.96] | 59.2% | 90.9% | 96.6% | 99% |
| Lancet Oncol 2009; 10: 459–66 | 0.49 [0.32;0.75] | 98.8% | 99.7% | 99.9% | 99.9% |
| JAMA. 2015;314(23):2535-2543 | 0.74 [0.56;0.98] | 70.1% | 91.3% | 95.9% | 98.2% |
| N Engl J Med 2015; 373:737-746 | 0.61 [0.47;0.79] | 97.7% | 99.8% | 99.9% | 100% |
| Lancet Oncol 2015; 16: 499–508 | 0.84 [0.73;0.98] | 23.4% | 80.9% | 94.4% | 98.9% |
| Lancet 2016; 388: 488–97 | 0.46 [0.3;0.71] | 99.4% | 99.9% | 100% | 100% |
| N Engl J Med 2014;370:734-43 | 0.71 [0.54;0.94] | 79.8% | 95% | 97.9% | 99.2% |
| Lancet Oncol 2015; 16: 763–74 | 0.84 [0.74;0.96] | 22.9% | 85.2% | 96.7% | 99.6% |
| Lancet Oncol 2015; 16: 1493–1505 | 0.79 [0.63;0.99] | 54.2% | 87% | 94.4% | 97.9% |
| J Clin Oncol 27:2938-2945 | 0.83 [0.69;0.99] | 34.2% | 80.9% | 92.8% | 98% |
| J Clin Oncol 35:3030-3038 | 0.68 [0.51;0.9] | 86.8% | 97.4% | 99% | 99.6% |
| N Engl J Med 2010; 362:1273-1281 | 0.64 [0.52;0.79] | 97.9% | 99.9% | 100% | 100% |
| J Clin Oncol 29:2011-2019 | 0.88 [0.78;1] | 7.3% | 65.2% | 89.1% | 97.9% |
| J Clin Oncol 30:3499-3506 | 0.82 [0.71;0.94] | 38% | 91.7% | 98.5% | 99.8% |
| N Engl J Med 2012; 366:2074-2084 | 0.66 [0.5;0.87] | 91.5% | 98.6% | 99.5% | 99.9% |
| N Engl J Med 2018; 378:230-240 | 0.67 [0.48;0.94] | 84.6% | 95.7% | 97.9% | 99% |
| N Engl J Med 2012; 367:1783-1791 | 0.68 [0.55;0.84] | 93% | 99.5% | 99.8% | 100% |
| N Engl J Med 2012; 366:799-807 | 0.5 [0.25;0.99] | 90.9% | 95.4% | 96.8% | 97.7% |
| N Engl J Med 2013;369:1691-703 | 0.72 [0.62;0.83] | 92% | 99.8% | 100% | 100% |
| Lancet 2016; 387: 545–57 | 0.67 [0.49;0.92] | 86.6% | 96.7% | 98.4% | 99.3% |
| Lancet 2011; 378: 2104–11 | 0.77 [0.61;0.98] | 62.6% | 89.9% | 95.9% | 98.4% |
| Lancet Oncol 2016; 17: 943–55 | 0.48 [0.29;0.8] | 97.6% | 99.2% | 99.6% | 99.8% |
| J Clin Oncol 36:3110-3119 | 0.63 [0.42;0.96] | 86.4% | 95.2% | 97.2% | 98.5% |
| Lancet 2009; 373: 301–08 | 0.68 [0.52;0.89] | 88.3% | 98.1% | 99.3% | 99.8% |
| Lancet Oncol 2014; 15: 1224–35 | 0.81 [0.68;0.96] | 45.8% | 89.1% | 96.6% | 99.3% |
| J Clin Oncol 36:2926-2934 | 0.63 [0.41;0.97] | 85.3% | 94.5% | 96.8% | 98.1% |
| Lancet Oncol 2013; 14: 777–86 | 0.79 [0.64;0.98] | 54.3% | 87.9% | 95.1% | 98.3% |
| J Clin Oncol 36:350-358 | 0.79 [0.63;1] | 54.4% | 86.3% | 93.8% | 97.6% |
| J Clin Oncol 36:2796-2803 | 0.71 [0.53;0.96] | 78.2% | 94% | 97.2% | 98.8% |
| J Clin Oncol 31:2128-2135 | 0.59 [0.36;0.97] | 88.5% | 95.3% | 97% | 98.2% |
| J Clin Oncol 29:1715-1721 | 0.69 [0.5;0.95] | 81.8% | 94.9% | 97.5% | 98.9% |
| J Clin Oncol 31:1931-1938 | 0.54 [0.33;0.89] | 93.9% | 97.9% | 98.7% | 99.3% |
| JAMA Oncol. 2018;4(5):661-669 | 0.61 [0.38;0.98] | 86.8% | 94.6% | 96.7% | 97.9% |
| Lancet Oncol 2012; 13: 993–1001 | 0.56 [0.34;0.94] | 91.3% | 96.4% | 97.7% | 98.7% |
| Lancet 2016; 387: 1405–14 | 0.77 [0.62;0.96] | 63.5% | 92.1% | 97.1% | 99.1% |
| Lancet Oncol 2015; 16: 320–27 | 0.4 [0.21;0.77] | 98.2% | 99.3% | 99.6% | 99.7% |
| Lancet Oncol 2017; 18: 297–311 | 0.62 [0.42;0.92] | 89.8% | 96.8% | 98.3% | 99.2% |
| Lancet 2016; 388: 1883–92 | 0.62 [0.45;0.85] | 94.4% | 99% | 99.6% | 99.8% |
| Lancet Oncol 2017; 18: 371–83 | 0.63 [0.45;0.88] | 91.6% | 98.2% | 99.2% | 99.7% |
| N Engl J Med 2019; 381:1124-1135 | 0.43 [0.24;0.78] | 98.1% | 99.3% | 99.6% | 99.8% |
| J Clin Oncol 33:2197-2204 | 0.68 [0.5;0.92] | 84.5% | 96.2% | 98.4% | 99.2% |
| Lancet Oncol 2019; 20: 282–96 | 0.71 [0.53;0.95] | 78.5% | 94.4% | 97.5% | 99% |
| J Clin Oncol 31:2849-2853 | 0.62 [0.46;0.83] | 95.5% | 99.4% | 99.8% | 99.9% |

Below we show the results from the first sensitivity analysis, where we used a prior $\mu_{i}\sim N(0, \sigma^{2}=1/5)$ for the effect (log of the hazard ratio).

eTable 12: Analysis results from 234 estimates of HR in oncology trials using a prior $logHR\sim N(0, \sigma^{2}=1/5)$

| **Study** | **Estimated Effect size**  **[95% CrI]** | $\boldsymbol{P(HR<0.80)}$ | $\boldsymbol{P(HR<0.90)}$ | $\boldsymbol{P(HR<0.95)}$ | $\boldsymbol{P(HR<1.00)}$ |
| --- | --- | --- | --- | --- | --- |
| JAMA. 2010;304(19):2154-2160 | 0.58 [0.38;0.89] | 92.8% | 97.7% | 98.8% | 99.3% |
| N Engl J Med 2018; 379:54-63 | 0.77 [0.64;0.92] | 66.6% | 95.2% | 98.8% | 99.7% |
| Lancet 2019; 393: 1948–57 | 0.78 [0.65;0.94] | 60.8% | 93.3% | 98.2% | 99.6% |
| J Clin Oncol 27:5062-5067 | 0.84 [0.72;0.98] | 24.4% | 79.5% | 93.5% | 98.6% |
| J Clin Oncol 34:972-979 | 0.71 [0.55;0.92] | 81.9% | 96.5% | 98.8% | 99.6% |
| J Clin Oncol 27:3109-3116 | 0.85 [0.72;1] | 25.8% | 76.6% | 91.4% | 97.5% |
| N Engl J Med 2018; 379:2342-235 | 0.72 [0.51;1.02] | 71.6% | 89.4% | 94.1% | 96.9% |
| Lancet Oncol 2016; 17: 1248–60 | 0.72 [0.57;0.91] | 81.6% | 96.8% | 98.9% | 99.7% |
| Lancet Oncol 2017; 18: 611–22 | 0.85 [0.71;1] | 25.8% | 76% | 90.8% | 97.3% |
| Lancet Oncol 2012; 13: 671–78 | 0.72 [0.51;1.01] | 72.8% | 89.9% | 94.4% | 97.2% |
| J Clin Oncol 30:2718-2724 | 0.58 [0.35;0.96] | 89.3% | 95.7% | 97.3% | 98.4% |
| Lancet Oncol 2019; 20: 494–503 | 0.64 [0.39;1.04] | 82% | 91.7% | 94.6% | 96.6% |
| Lancet 2010; 376: 687–97 | 0.75 [0.61;0.92] | 72.7% | 96% | 98.9% | 99.7% |
| Lancet 2012; 379: 315–21 | 0.75 [0.55;1.02] | 66.3% | 87.9% | 93.5% | 96.7% |
| J Clin Oncol 33:3858-3865 | 0.63 [0.42;0.95] | 86.9% | 95.6% | 97.6% | 98.6% |
| N Engl J Med 2012; 366:109-119 | 0.67 [0.5;0.9] | 87.2% | 97.3% | 98.9% | 99.6% |
| Lancet Oncol 2015; 16: 1037–48 | 0.68 [0.51;0.9] | 87.9% | 97.7% | 99.1% | 99.7% |
| N Engl J Med 2014; 371:424-433 | 0.72 [0.61;0.85] | 89.7% | 99.6% | 99.9% | 100% |
| J Clin Oncol 27:4454-4461 | 0.79 [0.63;1] | 52.5% | 85.2% | 93.3% | 97.3% |
| N Engl J Med 2017; 376:1015-1026 | 0.74 [0.6;0.92] | 75.3% | 96.2% | 98.9% | 99.7% |
| N Engl J Med 2014; 371:906-917 | 0.79 [0.65;0.96] | 54.9% | 90.1% | 96.7% | 99.1% |
| Lancet Oncol 2013; 14: 29–37 | 0.82 [0.7;0.95] | 40.2% | 89.8% | 97.5% | 99.5% |
| J Clin Oncol 30:2585-2592 | 0.76 [0.59;0.97] | 66.4% | 91% | 96.1% | 98.5% |
| Lancet Oncol 2010; 11: 1066–73 | 0.63 [0.48;0.83] | 95.5% | 99.5% | 99.8% | 100% |
| Lancet Oncol 2010; 11: 21–28 | 0.75 [0.58;0.96] | 69.9% | 92.4% | 96.8% | 98.8% |
| N Engl J Med 2015;373:1627-39 | 0.74 [0.61;0.9] | 78.4% | 97.8% | 99.5% | 99.9% |
| N Engl J Med 2015;373:123-35 | 0.62 [0.47;0.82] | 96.3% | 99.5% | 99.9% | 100% |
| Lancet 2017; 389: 56–66 | 0.65 [0.52;0.81] | 96.7% | 99.8% | 100% | 100% |
| N Engl J Med 2016;374:1344-55 | 0.63 [0.46;0.87] | 92.8% | 98.5% | 99.4% | 99.8% |
| N Engl J Med 2015; 373:2425-2437 | 0.53 [0.26;1.08] | 87.3% | 92.8% | 94.7% | 96.1% |
| J Clin Oncol 28:29-34 | 0.79 [0.63;1] | 52.7% | 85.7% | 93.7% | 97.5% |
| N Engl J Med 2014; 371:213-223 | 0.56 [0.34;0.92] | 92% | 96.9% | 98.1% | 98.9% |
| Lancet Oncol 2010; 11: 521–29 | 0.82 [0.7;0.95] | 40% | 90.2% | 97.7% | 99.6% |
| Lancet 2012; 379: 1508–16 | 0.73 [0.52;1] | 72.3% | 90.5% | 94.9% | 97.5% |
| Lancet Oncol 2018; 19: 1247–58 | 0.69 [0.49;0.97] | 80.1% | 93.6% | 96.7% | 98.4% |
| Lancet Oncol 2009; 10: 25–34 | 0.71 [0.53;0.95] | 78.9% | 94.5% | 97.5% | 98.9% |
| J Clin Oncol 36:2259-2266 | 0.71 [0.51;0.99] | 76.4% | 92.1% | 95.9% | 98% |
| N Engl J Med 2015;373:1814-23 | 0.7 [0.53;0.91] | 85.4% | 97.2% | 99% | 99.6% |
| Lancet 2009; 374: 1432–40 | 0.8 [0.66;0.96] | 51% | 89.9% | 96.7% | 99.1% |
| Lancet 2019; 393: 156–67 | 0.81 [0.66;0.99] | 45.3% | 85% | 94.2% | 98.1% |
| N Engl J Med 2011; 364:1817-1825 | 0.59 [0.47;0.75] | 99.4% | 100% | 100% | 100% |
| N Engl J Med 2018; 379:2395-2406 | 0.67 [0.51;0.88] | 89.8% | 98.3% | 99.4% | 99.8% |
| J Clin Oncol 29:3247-3254 | 0.7 [0.5;0.98] | 78.4% | 93% | 96.4% | 98.2% |
| Lancet 2011; 377: 914–23 | 0.82 [0.67;1] | 40.9% | 82.6% | 93% | 97.5% |
| Lancet Oncol 2015; 16: 1306–15 | 0.82 [0.67;1] | 41.4% | 82.7% | 93% | 97.5% |
| (online) | 0.69 [0.54;0.88] | 88.2% | 98.4% | 99.5% | 99.9% |
| Lancet 2010; 376: 1147–54 | 0.71 [0.6;0.84] | 92.2% | 99.7% | 100% | 100% |
| N Engl J Med 2011; 364:1995-2005 | 0.66 [0.56;0.79] | 98.4% | 100% | 100% | 100% |
| Lancet 2015; 385: 1863–72 | 0.67 [0.53;0.85] | 92.5% | 99.2% | 99.8% | 99.9% |
| Lancet Oncol 2017; 18: 1327–37 | 0.8 [0.66;0.97] | 49.7% | 88.2% | 95.9% | 98.8% |
| N Engl J Med 2013; 369:1023-1034 | 0.79 [0.63;1] | 53% | 86.6% | 94.2% | 97.7% |
| Lancet Oncol 2016; 17: 727–37 | 0.67 [0.41;1.1] | 76.7% | 88.2% | 92% | 94.5% |
| J Clin Oncol 29:1678-1685 | 0.71 [0.52;0.97] | 76.6% | 92.9% | 96.5% | 98.4% |
| Lancet Oncol 2018; 19: 1315–27 | 0.78 [0.6;1] | 58.8% | 87.3% | 94.1% | 97.5% |
| Lancet Oncol 2018; 19: 1315–27 | 0.63 [0.5;0.81] | 96.6% | 99.7% | 99.9% | 100% |
| Lancet 2017; 389: 519–27 | 0.74 [0.55;0.98] | 71.7% | 91.5% | 96% | 98.3% |
| J Clin Oncol 33:4138-4144 | 0.67 [0.48;0.94] | 84.6% | 95.8% | 98% | 99.1% |
| N Engl J Med 2016;375:1845-55 | 0.73 [0.6;0.9] | 80% | 97.6% | 99.4% | 99.9% |
| J Clin Oncol 33:3781-7 | 0.73 [0.53;1.01] | 70.4% | 89.6% | 94.6% | 97.3% |
| Lancet Oncol 2011; 12: 1204–13 | 0.69 [0.49;0.97] | 79.5% | 93.4% | 96.5% | 98.2% |
| Lancet 2016; 387: 1837–46 | 0.76 [0.56;1.02] | 64.5% | 87.6% | 93.5% | 96.8% |
| J Clin Oncol 28:562-569 | 0.57 [0.36;0.89] | 93% | 97.8% | 98.8% | 99.3% |
| N Engl J Med 2009; 361:1249-1259 | 0.75 [0.62;0.91] | 73.6% | 96.5% | 99.1% | 99.8% |
| N Engl J Med 2016;375:1856-67 | 0.72 [0.55;0.95] | 76.4% | 94.2% | 97.5% | 99% |
| N Engl J Med 2017; 377:352-360 | 0.64 [0.52;0.77] | 99% | 100% | 100% | 100% |
| N Engl J Med 2019; 380:1235-1246 | 0.74 [0.54;1.02] | 67.8% | 88.3% | 93.5% | 96.6% |
| N Engl J Med 2012; 367:107-114 | 0.64 [0.41;0.99] | 84.3% | 93.7% | 96.1% | 97.7% |
| Lancet Oncol 2014; 15: 78–86 | 0.7 [0.52;0.94] | 80.6% | 94.9% | 97.8% | 99% |
| Lancet 2014; 383: 31–39 | 0.79 [0.62;1.01] | 53.8% | 85.3% | 93.2% | 97.2% |
| N Engl J Med 2014; 370:997-1007 | 0.62 [0.31;1.23] | 76.9% | 85.6% | 88.8% | 91.2% |
| N Engl J Med 2018; 378:2078-2092 | 0.52 [0.41;0.67] | 100% | 100% | 100% | 100% |
| J Clin Oncol 29:2528-2533 | 0.64 [0.42;0.97] | 85.4% | 94.7% | 96.9% | 98.3% |
| Lancet 2014; 384: 665–73 | 0.86 [0.76;0.99] | 12.9% | 73.7% | 92.4% | 98.6% |
| Lancet Oncol 2014; 15: 1351–60 | 0.52 [0.34;0.8] | 97.3% | 99.4% | 99.7% | 99.9% |
| N Engl J Med 2014; 370:1101-1110 | 0.54 [0.33;0.87] | 94.5% | 98.2% | 99% | 99.4% |
| Lancet 2013; 382: 1021–28 | 0.77 [0.66;0.89] | 71.2% | 98.2% | 99.7% | 100% |
| Lancet Oncol 2016; 17: 1147–57 | 0.69 [0.54;0.9] | 85.9% | 97.5% | 99.1% | 99.7% |
| Lancet 2013; 381: 303–12 | 0.78 [0.65;0.94] | 60.7% | 93.5% | 98.1% | 99.6% |
| J Clin Oncol 31:1947-1953 | 0.76 [0.6;0.95] | 68.8% | 92.9% | 97.3% | 99.1% |
| J Clin Oncol 30:4344-4351 | 0.75 [0.58;0.98] | 67.9% | 90.8% | 95.9% | 98.2% |
| Lancet 2010; 376: 1164–74 | 0.7 [0.52;0.95] | 79.6% | 94.4% | 97.3% | 98.8% |
| JAMA Oncol. 2018;4(11):1569-1575 | 0.7 [0.56;0.88] | 87.6% | 98.5% | 99.6% | 99.9% |
| JAMA Oncol. doi:10.1001/jamaoncol.2019.0250 | 0.39 [0.29;0.52] | 100% | 100% | 100% | 100% |
| Lancet Oncol 2014; 15: 1065–75 | 0.78 [0.63;0.97] | 58.3% | 90.3% | 96.4% | 98.9% |
| Lancet 2016; 387: 1540–50 | 0.72 [0.59;0.89] | 83.4% | 98.3% | 99.6% | 99.9% |
| Lancet 2016; 387: 1540–50 | 0.63 [0.51;0.77] | 98.9% | 100% | 100% | 100% |
| N Engl J Med 2010; 363:711-723 | 0.68 [0.53;0.88] | 88.4% | 98.3% | 99.4% | 99.8% |
| N Engl J Med 2010; 363:711-723 | 0.69 [0.56;0.86] | 90.2% | 99.1% | 99.8% | 100% |
| Lancet Oncol 2018; 19: 1480–92 | 0.56 [0.45;0.68] | 100% | 100% | 100% | 100% |
| Lancet Oncol 2018; 19: 1480–92 | 0.66 [0.55;0.81] | 97% | 99.9% | 100% | 100% |
| J Clin Oncol 30:2441-2448 | 0.72 [0.53;0.97] | 74.5% | 92.6% | 96.4% | 98.4% |
| N Engl J Med 2018; 379:2220-2229 | 0.72 [0.56;0.93] | 79.3% | 95.8% | 98.4% | 99.5% |
| Lancet 2019; 393: 229–40 | 0.7 [0.5;0.98] | 77.8% | 92.6% | 96.2% | 98% |
| J Clin Oncol 27:242-249 | 0.61 [0.31;1.23] | 76.9% | 85.7% | 88.9% | 91.5% |
| J Clin Oncol 34:3609-3617 | 0.84 [0.7;1] | 30.9% | 79.1% | 92.1% | 97.6% |
| JAMA Oncol. 2018;4(4):483-492 | 0.75 [0.57;1] | 66.1% | 89.1% | 94.7% | 97.5% |
| Lancet 2016; 387: 1163–77 | 0.83 [0.7;0.98] | 35.7% | 84.3% | 94.9% | 98.7% |
| Lancet 2016; 387: 1163–77 | 0.79 [0.66;0.93] | 57.9% | 94.3% | 98.7% | 99.8% |
| N Engl J Med 2017; 377:338-351 | 0.64 [0.53;0.77] | 98.9% | 100% | 100% | 100% |
| JAMA 2012;307(12):1265-1272 | 0.61 [0.35;1.06] | 83.2% | 91.6% | 94.3% | 96.1% |
| J Clin Oncol 27:1177-1183 | 0.72 [0.53;0.98] | 73.8% | 91.6% | 95.7% | 98.1% |
| N Engl J Med 2011; 365:107-118 | 0.85 [0.74;0.99] | 18.9% | 76.3% | 92.8% | 98.5% |
| J Clin Oncol 30:1513-1518 | 0.69 [0.52;0.92] | 84.9% | 96.8% | 98.6% | 99.4% |
| Lancet 2017; 390: 2461–71 | 0.65 [0.53;0.8] | 97.8% | 99.9% | 100% | 100% |
| N Engl J Med 2017; 376:836-847 | 0.73 [0.57;0.94] | 75.9% | 94.9% | 98% | 99.3% |
| J Clin Oncol 28:1099-1105 | 0.63 [0.43;0.94] | 88.3% | 96% | 97.8% | 98.8% |
| N Engl J Med 2010; 363:411-422 | 0.79 [0.63;1] | 52.9% | 86.3% | 94.1% | 97.7% |
| Lancet 2009; 374: 1331–38 | 0.77 [0.59;1] | 61.8% | 88.4% | 94.6% | 97.7% |
| Lancet Oncol 2017; 18: 743–54 | 0.7 [0.56;0.87] | 89% | 98.9% | 99.8% | 100% |
| J Clin Oncol 33:1325-1333 | 0.67 [0.43;1.03] | 79% | 90.9% | 94.3% | 96.5% |
| J Clin Oncol 31:301-307 | 0.73 [0.52;1.03] | 69.5% | 88.2% | 93.2% | 96.5% |
| Lancet Oncol 2016; 17: 1579–89 | 0.75 [0.58;0.98] | 67.7% | 90.9% | 95.8% | 98.2% |
| N Engl J Med 2017; 377:1250-1260 | 0.64 [0.38;1.09] | 78.6% | 89.1% | 92.3% | 94.8% |
| J Clin Oncol 31:3219-3225 | 0.48 [0.3;0.75] | 98.9% | 99.8% | 99.9% | 99.9% |
| J Clin Oncol 31:3219-3225 | 0.53 [0.33;0.84] | 96% | 98.8% | 99.3% | 99.6% |
| JAMA. 2018;319(24):2486-2496 | 0.67 [0.53;0.85] | 93% | 99.3% | 99.8% | 100% |
| Lancet Oncol 2019; 20: 110–119 | 0.57 [0.39;0.82] | 96.7% | 99.3% | 99.7% | 99.9% |
| N Engl J Med 2014;371:1877-88 | 0.68 [0.47;0.98] | 80.3% | 93.2% | 96.2% | 98% |
| N Engl J Med 2017; 377:1813-1823 | 0.6 [0.45;0.81] | 97.1% | 99.7% | 99.9% | 100% |
| Lancet Oncol 2011; 12: 153–59 | 0.76 [0.6;0.95] | 68.4% | 93% | 97.4% | 99.2% |
| Lancet Oncol 2013; 14: 72–80 | 0.75 [0.62;0.91] | 74.4% | 96.9% | 99.3% | 99.9% |
| Lancet Oncol 2012; 13: 916–26 | 0.73 [0.55;0.96] | 75.5% | 93.7% | 97.2% | 98.9% |
| N Engl J Med 2017; 376:2147-2159 | 0.65 [0.44;0.95] | 86.2% | 95.5% | 97.6% | 98.7% |
| N Engl J Med 2013; 369:438-447 | 0.64 [0.32;1.27] | 74.6% | 84.2% | 87.7% | 90.3% |
| N Engl J Med 2015;372:1909-19 | 0.69 [0.58;0.81] | 96.3% | 99.9% | 100% | 100% |
| Lancet Oncol 2014; 15: 323–32 | 0.71 [0.58;0.88] | 86% | 98.6% | 99.7% | 99.9% |
| N Engl J Med 2012;366:1770-81 | 0.68 [0.46;1] | 79.8% | 92.2% | 95.4% | 97.5% |
| N Engl J Med 2012; 367:435-444 | 0.82 [0.66;1.01] | 40.9% | 81% | 91.8% | 97% |
| N Engl J Med 2012; 366:399-408 | 0.65 [0.38;1.12] | 77% | 87.8% | 91.3% | 93.9% |
| J Clin Oncol 28:2874-2880 | 0.78 [0.61;0.98] | 59.9% | 89% | 95.4% | 98.2% |
| J Clin Oncol 36:2244-2250 | 0.78 [0.6;1] | 58.4% | 86.8% | 93.7% | 97.3% |
| Lancet 2019; 393: 1819–30 | 0.81 [0.71;0.93] | 40.2% | 93% | 98.8% | 99.9% |
| Lancet 2019; 393: 1819–30 | 0.71 [0.58;0.86] | 89% | 99.1% | 99.8% | 100% |
| J Clin Oncol 28:3562-3569 | 0.71 [0.5;1.02] | 73.6% | 89.9% | 94.2% | 96.9% |
| Lancet 2010; 376: 1989–99 | 0.87 [0.77;0.99] | 8.7% | 68.8% | 91.1% | 98.4% |
| N Engl J Med 2015;373:1803-13 | 0.75 [0.59;0.94] | 72.1% | 94.2% | 97.9% | 99.3% |
| N Engl J Med 2018; 378:1277-1290 | 0.67 [0.48;0.93] | 85.1% | 95.9% | 98.1% | 99.1% |
| Lancet Oncol 2013; 14: 317–26 | 0.63 [0.38;1.05] | 82.1% | 91.6% | 94.3% | 96.3% |
| Lancet 2017; 389: 1011–24 | 0.83 [0.69;0.99] | 36.1% | 82.3% | 93.5% | 98.2% |
| JAMA. 2013;310(14):1473-1481 | 0.77 [0.62;0.96] | 62.1% | 91.7% | 96.9% | 99.1% |
| J Clin Oncol 32:2423-2429 | 0.69 [0.51;0.94] | 82.4% | 95.6% | 98% | 99.1% |
| N Engl J Med 2011; 364:205-214 | 0.64 [0.43;0.97] | 85.3% | 94.7% | 96.9% | 98.2% |
| Lancet Oncol 2015; 16: 928–36 | 0.79 [0.64;0.97] | 53.9% | 88.7% | 95.7% | 98.6% |
| J Clin Oncol 32:634-640 | 0.72 [0.55;0.95] | 76.4% | 94.2% | 97.5% | 99% |
| J Clin Oncol 31:1732-1739 | 0.71 [0.48;1.04] | 73.5% | 88.9% | 93.3% | 96.1% |
| Lancet 2010; 376: 2009–17 | 0.63 [0.42;0.93] | 88.2% | 96.2% | 97.9% | 98.9% |
| N Engl J Med 2013; 369:213-223 | 0.72 [0.57;0.9] | 82.7% | 97.6% | 99.3% | 99.8% |
| Lancet Oncol 2012; 13: 247–55 | 0.79 [0.65;0.96] | 54.7% | 90.2% | 96.7% | 99.1% |
| N Engl J Med 2018; 379:2040-2051 | 0.67 [0.51;0.87] | 91.2% | 98.8% | 99.6% | 99.9% |
| J Clin Oncol 31:426-432 | 0.6 [0.4;0.89] | 92.2% | 97.7% | 98.8% | 99.4% |
| N Engl J Med 2017;376:1027-37 | 0.68 [0.57;0.81] | 96.5% | 99.9% | 100% | 100% |
| Lancet 2009; 373: 1525–31 | 0.87 [0.77;1] | 9.6% | 66.9% | 89.2% | 97.8% |
| Lancet Oncol 2019; 20: 663–73 | 0.77 [0.6;0.98] | 62.8% | 89.7% | 95.5% | 98.2% |
| J Clin Oncol 36:3031-3039 | 0.77 [0.62;0.97] | 61.7% | 91.2% | 96.6% | 98.8% |
| Lancet 2011; 378: 1079–88 | 0.65 [0.54;0.8] | 97.7% | 99.9% | 100% | 100% |
| N Engl J Med 2011; 364:501-513 | 0.61 [0.34;1.08] | 83% | 91.1% | 93.7% | 95.5% |
| Lancet 2011; 378: 1858–67 | 0.55 [0.35;0.86] | 94.9% | 98.4% | 99.2% | 99.6% |
| N Engl J Med 2016;375:1823-33 | 0.65 [0.46;0.93] | 86.8% | 96% | 98.1% | 99.1% |
| Lancet Oncol 2011; 12: 1101–08 | 0.79 [0.7;0.91] | 54.8% | 97% | 99.7% | 100% |
| Lancet 2016; 387: 2402–11 | 0.61 [0.39;0.96] | 88.1% | 95.3% | 97.2% | 98.3% |
| N Engl J Med 2019; 380:1116-1127 | 0.57 [0.42;0.78] | 98.2% | 99.7% | 99.9% | 100% |
| Lancet 2017; 389: 255–65 | 0.74 [0.62;0.88] | 81.6% | 98.7% | 99.8% | 100% |
| Lancet Oncol 2018; 19: 1449–58 | 0.68 [0.53;0.87] | 89.8% | 98.7% | 99.6% | 99.9% |
| N Engl J Med 2011; 364:2517-2526 | 0.73 [0.6;0.88] | 82.5% | 98.5% | 99.7% | 99.9% |
| N Engl J Med 2015;372:30-9 | 0.71 [0.55;0.91] | 82.4% | 96.8% | 98.8% | 99.6% |
| N Engl J Med 2015; 372:320-330 | 0.47 [0.34;0.64] | 99.9% | 100% | 100% | 100% |
| N Engl J Med 2015;372:2521-32 | 0.66 [0.5;0.86] | 92% | 98.8% | 99.6% | 99.9% |
| N Engl J Med 2015;372:2521-32 | 0.71 [0.55;0.93] | 80.2% | 95.9% | 98.4% | 99.4% |
| J Clin Oncol 37:1159-1168 | 0.72 [0.53;1] | 73.3% | 91.2% | 95.3% | 97.7% |
| N Engl J Med 2013; 368:138-148 | 0.76 [0.62;0.94] | 67.8% | 94.3% | 98.2% | 99.6% |
| J Clin Oncol 31:448-455 | 0.71 [0.58;0.86] | 88.5% | 99.1% | 99.8% | 100% |
| J Clin Oncol 29:4387-4393 | 0.68 [0.56;0.84] | 93.3% | 99.6% | 99.9% | 100% |
| J Clin Oncol 30:172-178 | 0.68 [0.48;0.96] | 82.4% | 94.5% | 97.1% | 98.6% |
| N Engl J Med 2012; 367:1187-1197 | 0.64 [0.54;0.76] | 99.4% | 100% | 100% | 100% |
| N Engl J Med 2018; 379:2108-2121 | 0.66 [0.48;0.89] | 90.2% | 97.8% | 99.1% | 99.6% |
| J Clin Oncol 33:3733-3740 | 0.84 [0.71;0.99] | 30.8% | 80.4% | 92.9% | 98% |
| Lancet 2016; 387: 1629–37 | 0.78 [0.64;0.96] | 58.9% | 91.2% | 96.8% | 99.1% |
| N Engl J Med 2018;378:1107-20 | 0.62 [0.37;1.04] | 82.9% | 91.8% | 94.6% | 96.4% |
| J Clin Oncol 28:4581-4586 | 0.6 [0.35;1.03] | 85.2% | 93% | 95.3% | 96.9% |
| N Engl J Med 2017; 376:417-428 | 0.79 [0.61;1.01] | 55.8% | 85.9% | 93.4% | 97.2% |
| Lancet Oncol 2018; 19: 1437–48 | 0.7 [0.57;0.87] | 89% | 99.1% | 99.8% | 100% |
| JAMA Oncol. 2018;4(4):506-513 | 0.83 [0.69;0.99] | 36% | 82.4% | 93.9% | 98.2% |
| N Engl J Med 2011; 365:1273-1283 | 0.66 [0.51;0.85] | 93.6% | 99.3% | 99.8% | 99.9% |
| N Engl J Med 2011; 365:1273-1283 | 0.79 [0.62;0.99] | 56.2% | 86.9% | 94.3% | 97.8% |
| J Clin Oncol 30:2327-2333 | 0.77 [0.64;0.93] | 66.9% | 95.1% | 98.7% | 99.7% |
| N Engl J Med 2018; 378:2288-2301 | 0.79 [0.65;0.96] | 54.9% | 90.2% | 96.6% | 99.1% |
| Lancet Oncol 2015; 16: 897–907 | 0.81 [0.7;0.95] | 40.7% | 89.2% | 97.1% | 99.4% |
| N Engl J Med 2018; 378:113-125 | 0.67 [0.49;0.91] | 87.1% | 96.9% | 98.6% | 99.4% |
| J Clin Oncol 27:1788-1793 | 0.55 [0.33;0.91] | 92.7% | 97.1% | 98.3% | 99% |
| N Engl J Med 2015; 372:142-152 | 0.8 [0.64;1] | 48.8% | 84.8% | 93.4% | 97.5% |
| J Clin Oncol 29:4498-4504 | 0.74 [0.57;0.96] | 72.6% | 93.3% | 97.2% | 98.9% |
| N Engl J Med 2017; 377:454-464 | 0.79 [0.64;0.97] | 54.3% | 89.2% | 96% | 98.7% |
| Lancet Oncol 2009; 10: 459–66 | 0.56 [0.38;0.83] | 96.1% | 99.1% | 99.6% | 99.8% |
| JAMA. 2015;314(23):2535-2543 | 0.76 [0.58;0.99] | 64.7% | 89.3% | 94.9% | 97.7% |
| N Engl J Med 2015; 373:737-746 | 0.64 [0.49;0.82] | 96.3% | 99.6% | 99.9% | 100% |
| Lancet Oncol 2015; 16: 499–508 | 0.85 [0.74;0.98] | 21.2% | 79.3% | 94.1% | 98.9% |
| Lancet 2016; 388: 488–97 | 0.53 [0.36;0.79] | 98% | 99.6% | 99.8% | 99.9% |
| N Engl J Med 2014;370:734-43 | 0.73 [0.56;0.96] | 73.6% | 93.4% | 97.2% | 98.8% |
| Lancet Oncol 2015; 16: 763–74 | 0.84 [0.74;0.96] | 21.1% | 84% | 96.6% | 99.5% |
| Lancet Oncol 2015; 16: 1493–1505 | 0.8 [0.64;1] | 49.2% | 85% | 93.5% | 97.5% |
| J Clin Oncol 27:2938-2945 | 0.84 [0.7;1] | 31.1% | 79.2% | 92.3% | 97.6% |
| J Clin Oncol 35:3030-3038 | 0.7 [0.54;0.92] | 82% | 96.3% | 98.5% | 99.5% |
| N Engl J Med 2010; 362:1273-1281 | 0.66 [0.53;0.81] | 96.6% | 99.8% | 100% | 100% |
| J Clin Oncol 29:2011-2019 | 0.88 [0.78;1] | 6.6% | 63.4% | 88.4% | 97.7% |
| J Clin Oncol 30:3499-3506 | 0.82 [0.72;0.94] | 35.1% | 91% | 98.3% | 99.8% |
| N Engl J Med 2012; 366:2074-2084 | 0.68 [0.52;0.89] | 87.1% | 97.7% | 99.2% | 99.7% |
| N Engl J Med 2018; 378:230-240 | 0.7 [0.52;0.97] | 78.4% | 93.6% | 96.8% | 98.5% |
| N Engl J Med 2012; 367:1783-1791 | 0.7 [0.56;0.86] | 90.4% | 99.2% | 99.8% | 100% |
| N Engl J Med 2012; 366:799-807 | 0.65 [0.38;1.11] | 77.5% | 88.3% | 91.8% | 94.2% |
| N Engl J Med 2013;369:1691-703 | 0.73 [0.63;0.84] | 90.4% | 99.8% | 100% | 100% |
| Lancet 2016; 387: 545–57 | 0.7 [0.52;0.94] | 81.1% | 95% | 97.8% | 99.1% |
| Lancet 2011; 378: 2104–11 | 0.78 [0.62;0.98] | 57.4% | 88.5% | 95.1% | 98.3% |
| Lancet Oncol 2016; 17: 943–55 | 0.58 [0.37;0.9] | 92.9% | 97.6% | 98.6% | 99.3% |
| J Clin Oncol 36:3110-3119 | 0.69 [0.47;1.01] | 78.3% | 91.6% | 95.1% | 97.3% |
| Lancet 2009; 373: 301–08 | 0.7 [0.54;0.91] | 83.7% | 97.1% | 98.9% | 99.7% |
| Lancet Oncol 2014; 15: 1224–35 | 0.81 [0.69;0.97] | 41.9% | 87.4% | 96.1% | 99.1% |
| J Clin Oncol 36:2926-2934 | 0.69 [0.47;1.01] | 77.2% | 91% | 94.7% | 97% |
| Lancet Oncol 2013; 14: 777–86 | 0.8 [0.65;0.99] | 49.4% | 85.8% | 94.3% | 98% |
| J Clin Oncol 36:350-358 | 0.8 [0.64;1.01] | 49.4% | 84.4% | 92.9% | 97.2% |
| J Clin Oncol 36:2796-2803 | 0.74 [0.55;0.98] | 71.9% | 91.8% | 96.2% | 98.4% |
| J Clin Oncol 31:2128-2135 | 0.67 [0.44;1.04] | 78.8% | 90.9% | 94.2% | 96.3% |
| J Clin Oncol 29:1715-1721 | 0.72 [0.53;0.97] | 75.2% | 92.5% | 96.4% | 98.4% |
| J Clin Oncol 31:1931-1938 | 0.63 [0.41;0.96] | 86.7% | 95% | 97.1% | 98.3% |
| JAMA Oncol. 2018;4(5):661-669 | 0.68 [0.45;1.03] | 77.5% | 90.5% | 94.1% | 96.4% |
| Lancet Oncol 2012; 13: 993–1001 | 0.65 [0.42;1.01] | 82.1% | 92.6% | 95.4% | 97.2% |
| Lancet 2016; 387: 1405–14 | 0.78 [0.64;0.96] | 58.7% | 90.9% | 96.6% | 99% |
| Lancet Oncol 2015; 16: 320–27 | 0.55 [0.33;0.93] | 92% | 96.8% | 98% | 98.7% |
| Lancet Oncol 2017; 18: 297–311 | 0.67 [0.47;0.96] | 83.3% | 94.5% | 97.1% | 98.5% |
| Lancet 2016; 388: 1883–92 | 0.65 [0.49;0.88] | 90.8% | 98.4% | 99.3% | 99.8% |
| Lancet Oncol 2017; 18: 371–83 | 0.67 [0.49;0.91] | 86.9% | 96.8% | 98.7% | 99.5% |
| N Engl J Med 2019; 381:1124-1135 | 0.56 [0.34;0.9] | 92.9% | 97.5% | 98.5% | 99.1% |
| J Clin Oncol 33:2197-2204 | 0.71 [0.53;0.95] | 79% | 94.5% | 97.4% | 98.9% |
| Lancet Oncol 2019; 20: 282–96 | 0.74 [0.56;0.96] | 72.7% | 92.5% | 96.7% | 98.7% |
| J Clin Oncol 31:2849-2853 | 0.65 [0.49;0.86] | 92.5% | 98.8% | 99.6% | 99.9% |

# References

1.	Perneger, T. & Gayet-Ageron, A. Evidence of Lack of Treatment Efficacy Derived From Statistically Nonsignificant Results of Randomized Clinical Trials. *JAMA* **329**, 2050–2056 (2023).

2.	Perneger, T. V., Brindel, P., Combescure, C. & Gayet-Ageron, A. Evidence of survival benefit was often ambiguous in randomized trials of cancer treatments. *J. Clin. Epidemiol.* **127**, 1–8 (2020).

3.	Plummer, M., Stukalov, A. & Denwood, M. rjags: Bayesian Graphical Models using MCMC. (2024).

4.	R Core Team. R: A Language and Environment for Statistical Computing. R Foundation for Statistical Computing, Vienna, Austria. http://www.R-project.org (2014).

5.	Azzolina, D., Berchialla, P., Gregori, D. & Baldi, I. Prior Elicitation for Use in Clinical Trial Design and Analysis: A Literature Review. *Int. J. Environ. Res. Public. Health* **18**, 1833 (2021).

6.	Oakley, J. SHELF: Tools to Support the Sheffield Elicitation Framework. (2026).

7.	Morris, D. E., Oakley, J. E. & Crowe, J. A. A web-based tool for eliciting probability distributions from experts. *Environ. Model. Softw.* **52**, 1–4 (2014).

8.	Efthimiou, O. *et al.* An approach for modelling multiple correlated outcomes in a network of interventions using odds ratios. *Stat. Med.* **33**, 2275–2287 (2014).

9.	Grekov, P. & Pustejovsky, J. E. A Gentle Introduction to Bayesian Posterior Predictive Checking for Single-Case Researchers. *J. Behav. Educ.* https://doi.org/10.1007/s10864-025-09613-8 (2026) doi:10.1007/s10864-025-09613-8.

10.	Chang, W. *et al.* shiny: Web Application Framework for R. R package version 1.8.0. https://CRAN.R-project.org/package=shiny. (2021).
